# AnnoTALE: bioinformatics tools for identification, annotation, and nomenclature of TALEs from *Xanthomonas* genomic sequences

**DOI:** 10.1038/srep21077

**Published:** 2016-02-15

**Authors:** Jan Grau, Maik Reschke, Annett Erkes, Jana Streubel, Richard D. Morgan, Geoffrey G. Wilson, Ralf Koebnik, Jens Boch

**Affiliations:** 1 Institute of Computer Science, Martin Luther University Halle-Wittenberg, D-06120 Halle (Saale), Germany; 2 Department of Genetics, Martin Luther University Halle-Wittenberg, Weinbergweg 10, D-06120 Halle (Saale), Germany; 3 Department of Plant Biotechnology, Leibniz University Hannover, Herrenhäuser Str. 2, D-30419 Hannover, Germany; 4 New England Biolabs Inc., 240 Country Road, Ipswich, MA 01938, USA; 5 IRD, Cirad, Univ. Montpellier, Interactions Plantes Microorganismes Environnement (IPME) 34394 Montpellier France

## Abstract

Transcription activator-like effectors (TALEs) are virulence factors, produced by the bacterial plant-pathogen *Xanthomonas*, that function as gene activators inside plant cells. Although the contribution of individual TALEs to infectivity has been shown, the specific roles of most TALEs, and the overall TALE diversity in *Xanthomonas* spp. is not known. TALEs possess a highly repetitive DNA-binding domain, which is notoriously difficult to sequence. Here, we describe an improved method for characterizing *TALE* genes by the use of PacBio sequencing. We present ‘AnnoTALE’, a suite of applications for the analysis and annotation of *TALE* genes from *Xanthomonas* genomes, and for grouping similar TALEs into classes. Based on these classes, we propose a unified nomenclature for *Xanthomonas* TALEs that reveals similarities pointing to related functionalities. This new classification enables us to compare related TALEs and to identify base substitutions responsible for the evolution of TALE specificities.

*Xanthomonas* transcription activator-like effectors (TALEs) enhance bacterial virulence by acting as transcription factors within plant cells. TALEs contain a modular DNA-binding domain with a predictable and designable specificity^1^,^2^. The DNA-binding specificity of TALEs is determined by a central domain of near-perfect 33 to 35-amino acid-repeats. Each repeat aligns with one base pair of the bound DNA sequence, with two variable amino acids at position 12 and 13 (termed the ‘RVD’, for repeat-variable diresidue) acting as the determinants of base-recognition specificity^1^,^2^. X-ray crystallography of TALE-DNA complexes has revealed that only amino acid 13 actually contacts the DNA base and is the main determinant of specificity^3^,^4^, while the amino acid 12 forms inter-repeat contacts that contribute indirectly to the binding efficiency^3^,^4^. Certain RVDs (NI for A, HD for C, NG for T, and NN for G or A) are predominantly used in nature, but several others occur less frequently^5^. The DNA-specificities of all 400 theoretically possible RVD di-amino acid combinations have been determined experimentally^6^,^7^,^8^,^9^,^10^ and have confirmed that position 13 controls DNA recognition specificity. In addition, the so-called ‘strong RVDs’, HD and NN, form the strongest interaction with the DNA bases and are required for an overall efficient binding of TALEs to DNA^9^. The one base-one repeat specificity of TALEs is extended by one 5′ thymine^1^,^2^ that is specified by the N-terminal domain of TALEs. This domain also contributes to efficient DNA interaction^11^,^12^,^13^ and likely facilitates initial DNA contact. The unique modularity of TALEs has led to their widespread use as laboratory reagents for genome engineering and transcriptional reprogramming^14^,^15^. By choosing the appropriate number of repeats and RVD-types, practically any desired DNA-binding specificity can be engineered. In contrast to the well-documented use of TALEs in biotechnology, their natural diversity in plant-pathogenic *Xanthomonas* spp. bacteria is not well understood.

TALEs and related proteins are bacterial virulence factors found primarily in plant-pathogenic *Xanthomonas* spp. and *Ralstonia solanacearum* bacteria^5^. TALE proteins are secreted by the bacterial type III secretion system into plant cells where they localize to the plant nucleus and activate the expression of specific target genes. The N-terminal portion contains the type III secretion signal, and the C-terminal portion contains the nuclear localization signals and an acidic activation domain; all three are highly conserved among TALEs^5^. In contrast, the number of DNA-binding repeats and their RVDs vary greatly, suggesting that TALEs can activate a wide variety of different plant target genes.

Rice-pathogenic *Xanthomonas oryzae* pv. *oryzae* (hereafter termed ‘*Xoo*’) and *Xanthomonas oryzae* pv. *oryzicola* (*Xoc*) are serious threats to the agricultural production of rice^16^. Both pathogens typically harbor large TALE repertoires which, in case of *Xoo*, differ in number and nature between Asian (15 to 26 TALEs), African (8 to 10 TALEs), and North-American (no TALEs) strains^17^,^18^,^19^,^20^. Although some TALEs contribute significantly to pathogen virulence, the physiological role of most remains unclear^21^. Computational algorithms have used the RVD-based target specificity to predict possible TALE target sequences in rice promoters^22^,^23^,^24^, but biological confirmations of these predictions are for the most part lacking.

Three complete *Xoo* and one *Xoc* genomic sequences including *TALE* genes have been generated by classical Sanger sequencing^25^,^26^,^27^,^28^ and two draft genomes by Illumina sequencing^19^. Next-generation sequencing (NGS) methods like Illumina yield short reads unsuitable for the assembly of the highly repetitive *TALE* sequences. Recently, two genomic *TALE* genes were correctly assembled following Illumina sequencing of a *Xanthomonas translucens* pv. *cerealis* strain^29^, but the large number of *TALE* genes present in most *X. oryzae* strains precludes a similar strategy. The TALE repertoires in *Xoo* and *Xoc* are highly variable. At present we are unable to establish their diversity although this is essential for understanding the contribution of these potent virulence factors to pathogen performance.

The nomenclature of *Xanthomonas* TALEs has been non-uniform. Historically, individual *TALE* genes have been sub-cloned and assigned TALE names based on their reaction in resistant hosts (names starting with “Avr” for avirulence), their contribution to pathogenicity (names starting with “Pth” for pathogenicity), or their sequential location in the genome (e.g. Tal1, Tal2, Tal3, etc. or Tal9a, Tal9b, etc. if part of the same genomic locus). Because of frequent genomic rearrangements in different strains of *Xoo*^28^, the order of genomic loci, and thus TAL names, is not consistent. Consequently, the current TALE nomenclature fails to reveal similarities between TALEs that could indicate common host targets, and is profoundly confusing, because completely different TALEs can have identical names, and near-identical TALEs can have different names (e.g. Tal9c of strain *Xoo* PXO99^A^ and AvrXa27 are synonyms and 99.4% identical on DNA-level to Tal1c of strain *Xoo* MAFF3113018^28^).

Here, we demonstrate that the PacBio NGS method^30^ is uniquely suitable for sequencing *Xanthomonas* genomes that harbor a large number of *TALE* genes. This new approach enables rapid characterization of TALE repertoires revealing a pathogen’s complete virulence arsenal. To assist in our analysis, we have developed a suite of applications for predicting *TALE* genes in sequenced genomes, and for grouping TALEs into classes that indicate possible functional relationships to known TALEs. We envisage that a large number of *TALE* sequences will emerge from PacBio sequencing in the near future, and we propose a unified TALE nomenclature based on this classification. Our work contributes to a deeper understanding of plant-pathogen interactions, and could help reduce the damage wrought by Xanthomonads on domestic crops.

## Results and Discussion

### Selection of rice-pathogenic *Xanthomonas oryzae* pv. *oryzae* strains from the Philippines

Rice is grown in many areas of the world and is the predominant food crop of Asia. Accordingly, *Xanthomonas oryzae* pv. *oryzae* (*Xoo*) is a worldwide scourge, and different lineages exist in Africa, Asia, and North-America^18^. Rice lines resistant to specific isolates of *Xoo* have been cultivated, and several of these (e.g. Xa3, Xa7, Xa10, Xa23) stem from altered responses to TALEs. This, in turn, has led to selective pressure on *Xoo* to diversify its repertoire of TALEs.

**Figure 1:**
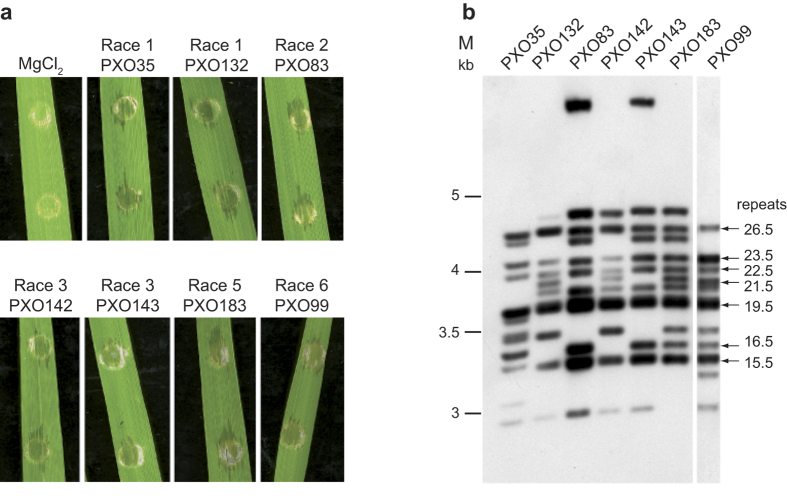
Phenotypes and race-specific *TALE* gene pattern of Philippine *Xoo* strains. (**a**) Leaves of 3- to 4-week old rice cultivar Nipponbare plants were infiltrated with 10 mM MgCl_2_ or Philippine *Xoo* strains PXO35 (race 1), PXO132 (race 1), PXO83 (race 2), PXO142 (race 3), PXO143 (race 3), PXO183 (race 5) and PXO99 (race 6), respectively. Plant reactions were documented five days post inoculation. (**b**) Southern blot analysis. Genomic DNA of the Philippine *Xoo* strains PXO35 (race 1), PXO132 (race 1), PXO83 (race 2), PXO142 (race 3), PXO143 (race 3), PXO183 (race 5) and the reference strain PXO99 (race 6) was digested with *Bam*HI, separated on an agarose gel, and transferred to a nylon membrane. *TALE*-containing fragments were detected using a DIG-labelled probe corresponding to 500 bp of the 3′ part of *talC*. Known repeat numbers of TALEs and their corresponding fragments in the sequenced *Xoo* strain PXO99 are indicated at the right side. The approximate sizes of marker fragments estimated from the ethidium bromide-stained gel are indicated at the left side. The large fragments in lanes 3 and 5 (PXO83 and PXO143) are due to an absence of the typically conserved *Bam*HI sites in certain *TALEs*.

For the study reported here, we focused on *Xoo* strains from the Philippines, a relatively confined region with a long-standing history of *Xoo*-rice interaction research^31^,^32^. Philippine *Xoo* strains have been classified into eleven races based on their pathogenicities on near-isogenic rice lines harboring individual resistance loci^18^,^31^. We chose six strains belonging to four races that are distantly related to each other as well as to the archetype sequenced strain, *Xoo* PXO99^A^ (Table 1^31^). All six strains cause disease in rice (*Oryza sativa* ssp. *japonica* cv. Nipponbare) (Fig. 1a). The *TALE* genes in these strains were initially analyzed by Southern blots of *Bam*HI-digested genomic DNA (Fig. 1b). *Bam*HI digestion distinguishes *TALEs* by size, mainly reflecting the varying number of repeats. This analysis revealed a non-identical, but apparently partially overlapping TALE repertoire.

### Decoding genomic *TALEs* using PacBio

*TALE* genes have traditionally been cloned *via* cosmid libraries, but this is a time-consuming method and does not guarantee that all possible *TALE* genes are identified. It is possible for *TALE* genes to be correctly assembled following Illumina sequencing, provided the number of *TALE* genes is small and the inter-repeat differences are substantial^29^, but this is not feasible for *Xoo* harboring numerous similar *TALE* genes. PCR-amplification and sub-cloning of individual *TALE* open reading frames is possible^29^, but encounters two problems. First, it cannot be excluded that truncated amplification products mis-prime on different *TALE* templates and in different repeat regions, generating artificial chimeric products. And second, PCR-amplification requires the primers to be complementary to conserved regions - typically the 5′ and 3′ regions of *TALE* genes - where they cannot amplify the flanking genomic context. Therefore, this method does not reveal genomic *TALE* loci, omitting an interesting piece of information to unravel evolutionary events during *TALE* genesis.

We chose PacBio sequencing to characterize and compare the *TALE* repertoire of *Xoo* strains, because this method is uniquely able to produce long reads from singular templates^30^. As proof of concept, we sequenced the genome of *Xoo* PXO83, a race 2 strain that clusters with race 3 and race 5 strains in RFLP analysis^31^ and which differs from the sequenced race 6 *Xoo* strain PXO99^A^. PacBio sequencing of approximately 170x coverage resulted in a single contig despite the presence of numerous repetitive elements (Table 2). Trimming contig ends and circularization yielded a final PXO83 chromosome of 5,025,428 bp (Genbank accession no. CP012947).

To assess the quality of the final PXO83 assembly, we performed a re-sequencing experiment using the same PacBio reads that were used for the assembly. We found a generally homogeneous coverage of the assembled chromosome (Supplementary Fig. S1), with a mean coverage of approximately 182. We investigated the stability of the results by artificially reducing the set of reads by sub-sampling (Supplementary Fig. S2). We found that almost the complete chromosome is covered by at least one read using only 5% of the PacBio data, while 99.89% of the chromosome is covered by at least 100 reads using all of the data (Supplementary Fig. S3). We assessed the concordance of base calls for these reduced sets (Supplementary Fig. S4) and the potential ambiguity of base calls (Supplementary Fig. S5) and found that 30% of the PacBio reads would have been sufficient to yield 100% concordance to the final chromosome with low ambiguity.

As we were especially interested in the *TALE* genes of PXO83, we additionally considered the mean (Supplementary Fig. S6) and position-specific (Supplementary Fig. S7) coverage of TALE repeats, and found that all TALEs are sufficiently covered, that coverage is largely uniform in successive repeats (Supplementary Fig. S8) and, importantly, that no divergent patterns are present for RVD-coding codon pairs. We did not observe a divergent pattern for RVD-coding positions regarding the ambiguity of base calls (Supplementary Fig. S9). Finally, we asked whether a sufficient number of PacBio reads completely spanned each TALE - important because of their repetitive nature - and found that each is spanned by at least 10 continuous reads (Supplementary Fig. S10).

**Figure 2:**
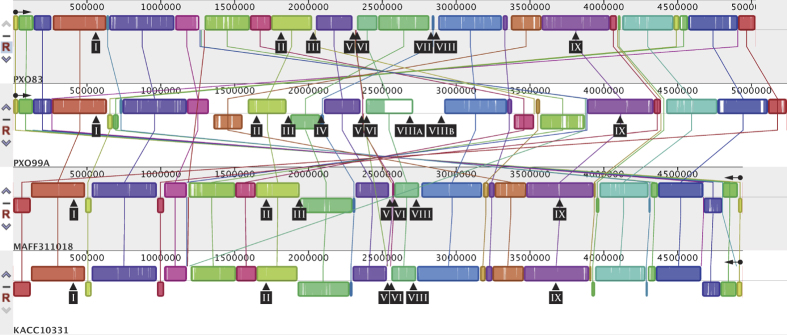
Comparison of *Xanthomonas oryzae* pv. *oryzae* genomic regions. ProgressiveMauve alignment of four fully sequenced genomes of *Xanthomonas oryzae* pv. *oryzae* (*Xoo*) strains. Similarly coloured areas represent genomic regions with significant synteny. The genomes of *Xoo* KACC10331 and *Xoo* MAFF311018 are shown in reverse complement to simplify the view, because of extensive genomic rearrangements. To indicate this, the origin and the orientation of *dnaA* is indicated by a black dot with horizontal arrow. Black arrowheads point to the positions of the genomic TALE clusters. The numbering is according to Fig. 5.

A progressiveMauve^33^ alignment of the *Xoo* PXO83 genome with the *Xoo* strains PXO99^A^, MAFF311018, and KACC10331 demonstrated that PXO83 is most closely related to PXO99^A^ with distinct rearranged regions (Fig. 2).

### Computational extraction and annotation of the genomic *TALE* repertoire

**Figure 3:**
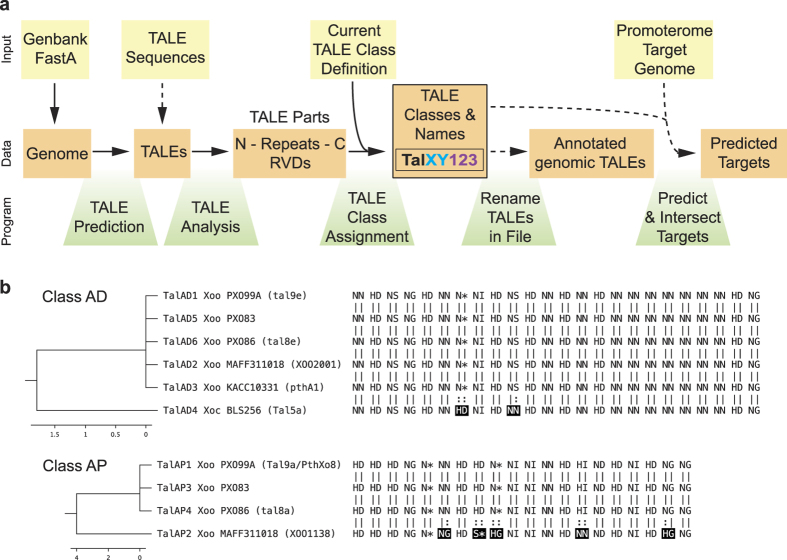
Workflow for AnnoTALE. (**a**) Diagram of input parameters, workflow, and program parts. The newly assigned names of TALEs consist of Tal (black), TALE class (XY, light blue), and allele number of TALE within a class (123, purple). Yellow boxes represent input parameter, orange boxes show generated data, and green boxes indicate the program parts that perform the individual job. To assign TALEs to existing classes the current class definition can be downloaded and imported into the program. Besides genomic sequences, individual *TALE* DNA sequences can optionally be loaded into the program. N, N-terminal region; C, C-terminal region; RVD, repeat variable di-residue. (**b**) Examples of the TALE Class Assignment tool. Two representative TALE classes, AD and AP, are displayed. RVD changes between TALEs are marked in black.

We developed a novel suite of applications, called “AnnoTALE”, for 1) identifying and analyzing TALEs in *Xanthomonas* genomes; 2) clustering TALEs into classes by their RVD sequences; 3) assigning novel TALEs to existing classes; 4) proposing TALE names using a unified nomenclature; and 5) predicting targets of individual TALEs and TALE classes (Fig. 3a). The suite of applications is available at “ http://www.jstacs.de/index.php/AnnoTALE” as a JavaFX-based stand-alone application with graphical user interface for interactive analysis sessions. In addition, we provide a command line application that may be integrated into other pipelines. Both are based on the open-source Java library, Jstacs^34^, and use identical code for the actual analysis, ensuring consistent results between both versions. In the following section, we use the newly sequenced genome of race 2 *Xoo* strain PXO83 and its TALE repertoire to exemplify the steps of the AnnoTALE pipeline (Fig. 3a).

### Predicting and analyzing *TALE* genes

AnnoTALE identifies *TALE* genes in *Xanthomonas* genomes based on the DNA sequence homology of individual TALE domains. The most prominent feature of TALEs is their DNA-binding domain, which comprises an array of highly conserved tandem repeats. Since the number of repeats is not known beforehand, we first scan the genome for individual repeats, which are then joined into contiguous stretches. At the 5′ and 3′ ends of these stretches, we search for occurrences of the TALE N- and C-domains, yielding a TALE open reading frame (ORF). For all three domains, we consider the homology on the DNA level to identify putative pseudogenes, which might be missed by comparing only amino acid sequences.

Finally, we refine the ORF so that the reported *TALE* gene starts with an ATG and ends with a stop codon. If this refined region is substantially shorter than the initial match, i.e., more than one third of the initial C- or N-terminal match is missing, we label it as a putative *TALE* pseudogene. Applying the “TALE Prediction” tool of AnnoTALE to the *Xoo* PXO83 genome, we found 16 full-length *TALE* genes and 2 pseudogenes (Table 3, Supplementary Table S1). Using the “TALE Analysis” tool, we split TALEs into their individual N- and C-terminal domains and repeats. This allows RVD sequences to be extracted and repeats of aberrant length to be identified^35^. Two of the 16 identified **TALE** genes of *Xoo* PXO83 have an aberrant long repeat, and the two pseudogenes have an aberrant short repeat (Supplementary Fig. S11b,d).

Recently, the genome sequence of *Xoo* strain PXO86, a close relative of strain PXO83, was published^36^. The authors used command line programs termed PBX toolkit to perform first a local assembly of *TALE* gene reads from PacBio raw data and to extract RVD sequences prior to assembly of the whole genome^36^. In contrast, AnnoTALE is a user-friendly tool collection with graphical user interface for characterizing TALEs in already assembled genomes.

### Building classes of known TALEs

To compare the *Xoo* PXO83 TALEs to those of the characterized *Xoo* and *Xoc* strains, and to individually sequenced TALEs (Supplementary Table S2), we developed a pairwise measure of TALE divergence. The basic intent is to group TALEs with related target specificity together, since this could signify related functionality. Because TALE-specificity primarily depends on the sequence of RVDs, we chose a measure based on the alignment of RVD sequences. Within the RVD alignment, mismatching amino acids at positions 12 and 13 are given penalties of 0.2 and 0.8, respectively, reflecting the importance of amino acid 13 for target specificity, and the minor, but measurable influence of amino acid 12 for activation efficiency^3^,^4^. Hence, if both amino acids of an RVD mismatch, we score this with a penalty of 1.0. Internal gaps are strongly penalized (gap opening penalty 5.0, extension 1.0), because these have a strong impact on target specificity. Comparing TALEs with differing numbers of repeats requires gaps at either side of the shorter TALE, which are allowed but also slightly penalized (opening 1.0, extension 0.1), because i) the additional RVDs of the longer TALE narrow target specificity and ii) very short TALEs might otherwise align to portions of very long TALEs with a good score just by chance. Using this measure of TALE divergence, we can now assign a divergence score to each pair of TALEs.

Alternatively, we could have compared TALEs by means of their predicted targets according to computational approaches^22^,^23^,^24^, which, however, will change with refined algorithms and compromise a stable TALE classification. In addition, a classification by RVDs has the advantage that to some extent it reflects evolutionary relationships. In contrast, two TALEs with similar target specificity can theoretically consist of different RVDs (signifying different evolutionary origin) due to the ambiguous specificities of certain RVDs^1^,^2^,^5^. In addition, we did not compare TALEs based on their full-length amino acid or nucleotide sequences, because it is one of the hallmarks of TALEs that novel specificities, and thereby novel physiological functions, arise frequently by recombination and repeat diversification. Therefore, a phylogenetic classification^37^ might not necessarily reflect TALE functionality.

Based on this pairwise score of TALE divergence, we grouped TALEs into different classes. Technically, we use a hierarchical, agglomerative clustering approach. By this means, we group TALEs together such that the average divergence score of TALEs in a common class does not exceed a threshold of T = 5.0 (see Methods). Instead of the average divergence, we could have used the minimum or maximum divergence score of TALEs in a common class. However, the former has the disadvantage of long “chains”, where finally two TALEs in a common class might only be linked by several TALEs with low pairwise divergence, whereas these very two TALEs themselves are highly divergent. The maximum divergence, in turn, might lead to a situation where a new TALE is not assigned to the class containing its least divergent peer, because other TALEs in the same class show a greater divergence. The choice of the average divergence balances between these two extremes, and often gives results that conform to intuition. The above procedure is implemented in the “TALE class builder” tool of AnnoTALE, which can be applied to cluster custom sets of TALEs into classes (Fig. 3).

### A universal TALE nomenclature

The first TALEs identified were named according to the resistance reaction they induce (e.g., AvrBs3, AvrXa7, AvrXa10), or to their contribution to pathogenicity (e.g., PthA, PthB, etc.). Because in principle the same resistance reaction can be triggered by different TALEs, or by TALEs that are related but carry non-identical repeat regions (e.g., different *pthXo2* alleles exist^38^), this naming scheme is not unambiguous. Subsequently, *TALEs* identified in whole genome sequences were named sequentially starting from the origin (*dnaA*) (e.g., *tal1*, *tal2a*, *tal2b*, etc.^28^), or according to size (*talA*, *talB*, *talC*, etc.^39^). Because large genomic rearrangements are frequent in *Xoo*^28^,^40^, and different *TALEs* can cluster at similar positions, this naming scheme can, and indeed already has, given the same name to different TALEs in different *Xoo* genomes. To rectify this situation, and to assign unambiguous names to the large number of *TALE* sequences we expect to be discovered in the future, we have developed rules for a unified nomenclature of TALEs.

This nomenclature is based on the assignment of TALEs into classes derived in the previous step. The name of each TALE starts with “Tal”, followed by a two-character identifier of its class (e.g., XY), and the number of the TALE within its class (Fig. 3a). While this nomenclature already uniquely identifies each TALE by its name, AnnoTALE also reports the strain origin of the TALEs within a class. Using this nomenclature, Tal9e from *Xoo* strain PXO99^A^ is re-named TalAD1, pthA1 from *Xoo* KACC10331 is re-named TalAD3, and XOO2001 from MAFF311018 is re-named TalAD2 (Fig. 3b). Using this new nomenclature, it becomes instantly clear that these three TALEs, whose previous names were completely different, are in fact closely related. In this particular example, the RVD sequences of all three TALEs are identical (Fig. 3b), suggesting that they recognize the same target sequence in the host and trigger the same reaction.

### Classification of PXO83 TALEs

The assignment of novel TALEs to existing classes is based on the same divergence score that was used to build these classes initially (see above). We first compute the average divergence between the novel TALE and each of the existing classes. If the average divergence score of the TALE and the most similar class does not exceed the threshold, the TALE is added to that class. Otherwise, it becomes the first member of a new class. This assignment is implemented by the “TALE Class Assignment” tool of AnnoTALE. Considering the TALEs of PXO83 identified in the work reported here, one TALE could not be assigned to any existing class and thus became the founding member of a new class, and was assigned the name TalCA1 (Supplementary Fig. S11f). All of the other TALEs show sufficient similarity to previously identified TALEs and were assigned to one or the other of the existing classes (Table 3; Supplementary Sequences).

While this manuscript was in preparation, two additional complete *Xanthomonas* ssp. genomes were published: *Xoo* PXO86 and *Xoc* CFBP7342 (Genbank accession nos. CP007166.1 and CP007221.1). We applied the AnnoTALE pipeline to these genomes as well, yielding a total of 62 classes for 143 TALEs. Two examples of these classes are shown in Fig. 3b. Five of the TALEs of class TalAD are completely identical and cluster with a slightly divergent TALE from *Xoc* (Fig. 3b). The relatedness suggests that these TALEs target the same plant genes, making it one of the few examples in which *Xoo* and *Xoc* share similar TALEs. In the second example, class TalAP TALEs from *Xoo* PXO99^A^, PXO83, and PXO86 have identical RVD sequences, while TalAP2 from MAFF311018 shows amino acid substitutions at RVDs 6, 8, 9, 14, and 19 (Fig. 3b) resulting in a divergence score of 4.0, close to the threshold of 5.0. This example demonstrates the utility of our classification to reveal similarities that are not immediately apparent.

### Predicting targets of TALE classes

Ultimately, we are interested in the function of *TALE* genes as virulence factors. To facilitate initial analyses of putative target genes of individual TALEs and common target genes of all members of a class, we incorporated target prediction into AnnoTALE. The “Predict and Intersect Targets” tool of AnnoTALE is based on the statistical model of TALgetter^23^ and uses speed-up techniques of TALENoffer^41^. It allows us to scan the rice promoterome for target sites of all 143 TALEs in less than five minutes on a standard PC. In contrast to TALgetter, however, our scan is limited to the top 100 target sites and does not include the computation of p-values of individual target sites. For a complete overview we used the full-scale option of the “Predict and Intersect Targets” tool and analyzed the rice promoterome target of the full “Class builder” (i.e., all TALEs in all classes). The list of targets can be found as Supplementary Table S4. Applying this tool to the TALEs in all 62 classes, we find, for instance, that the four TALEs in class TalAP share 11 target genes, of which the target gene *OsHEN1* of TalAP1 (PthXo8) and TalAP2 (XOO1138)^2^,^23^ is predicted on rank 1 for all four TALEs. Interestingly, *OsHEN1* is also the target gene of TalAK2 (Tal1c) from *Xoc* BLS256, but this TALE targets a different site within the *OsHEN1* promoter using different RVDs.

The current list of TALEs and their classifications can be accessed at www.xanthomonas.org. To reserve a name for a given TALE in the course of manuscript preparation, confidential requests can be sent to the corresponding authors of this paper. Following publication, these new names will be included in our updated web-based class list that is also used by AnnoTALE.

### Conservation and substitutions in RVD codon pairs

**Figure 4:**
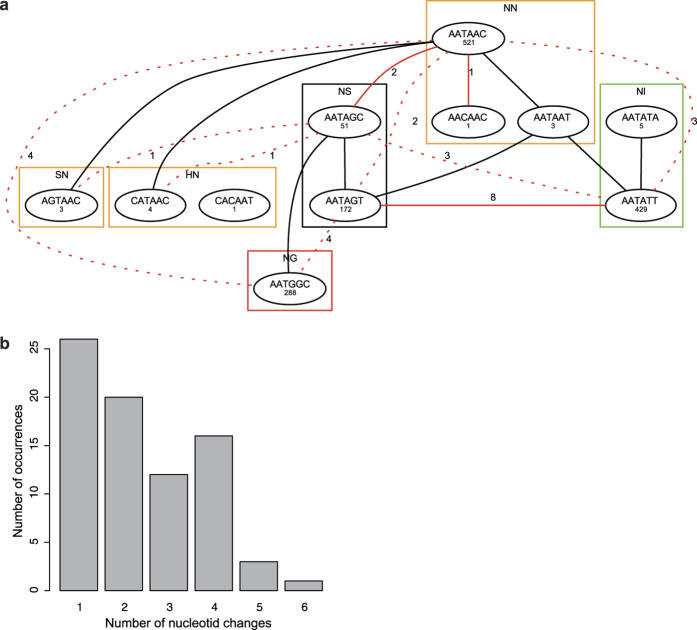
Changes in RVD codons. (**a**) Network of codon pairs of RVDs connected to the RVD NS by single-nucleotide or double-nucleotide substitutions. RVDs are boxed in colors according to their DNA base specificities (green: A, orange: G, red: T) and list the observed codon pairs. Frequency in known TALEs is shown below each codon pair. Lines represent possible single-nucleotide (solid line) or double-nucleotide (dotted line) exchanges. Substitutions observed within classes are in red with labels indicating the number of occurrences. (**b**) Histogram of the number of observed nucleotide substitutions in aligned RVDs of TALEs in a common family.

The clustering of TALEs into high-confidence classes allows new insights into the evolution of *TALE* genes. Considering all repeats of all TALEs, the RVDs are typically perceived as the most variable residues. However, the clustering of TALEs into classes reveals that RVDs of related TALEs are highly conserved between different *Xoo* and *Xoc* strains as well. For example, the RVDs of all TALEs in class AD (Fig. 3b) are completely conserved between the *Xoo* strains PXO99^A^, PXO83, PXO86, MAFF311018, and KACC10331, while we observe only two differing RVDs (HD at position 7 and NN at position 10) for *Xoc* BSL256. Conservation of RVDs is also present at the nucleotide level for the codons of the RVD amino acids. We systematically investigated which codon pairs are used to code for RVDs and found that only a small subset in fact occur. For instance, the RVD NS (Asn-Ser) can be encoded in principle by 12 different codon pairs, but only two of these occur in the TALEs considered (Fig. 4a). We make similar observations for all other RVDs, where only one to three codon pairs are used (Supplementary Table S3).

To trace evolutionary lineage, we used our TALE classification to compare codon pairs of the aligned RVD sequences of TALEs within each class, where the majority of RVDs are completely conserved between all class members, even on the codon level. We plot a histogram of the number of nucleotide substitutions in these RVD codon pairs in Fig. 4b and find that frequency decreases with increasing number of substitutions in RVD codons. In 26 cases, we observe a single-nucleotide substitution; 25 of these lead to a non-synonymous substitution of one of the AAs in an RVD, and one is a synonymous substitution. However, we also observe substitutions of a greater number of nucleotides in RVD codon pairs with surprisingly high frequency (e.g., 16 occurrences of 4 substitutions in RVD-coding nucleotides). As one specific example, we consider the RVDs NS and NN in the following. The network in Fig. 4a represents observed substitutions in the codon pair of RVD NS in all TALE classes. The only synonymous substitution appears in NN-codon pairs of TALE class BF (Supplementary Fig. S11e). Single-nucleotide substitutions are present between NN and NS in two cases, and between NS and NI in eight cases. Substitutions of two nucleotides arise between NS and SN, NS and HN, NS and NI, NS and NN, NS and NG, NN and NG, and NN and NI.

Finally, we examined some substitutions in the corresponding class-contexts. In class AA (Supplementary Fig. S11a), we found several single-nucleotide substitutions, including non-synonymous substitutions at repeat three, where four class-members have an NS repeat and the last two an NI repeat, and in repeat 19, which is an NS repeat only in the last TALE of this class and an NN repeat in the other TALEs. In contrast, class AQ (Supplementary Fig. S11d) displays substitutions of two nucleotides in repeat 5, which is NS in three TALEs and NG in one TALE. For repeat 8, which has an S* repeat in the same three TALEs and an NS repeat in the other, we observe the deletion of a complete codon. These observations suggest that one way in which TALE specificities evolve is by direct base substitutions in RVD codons.

### Allocating genomic TALE cluster

**Figure 5:**
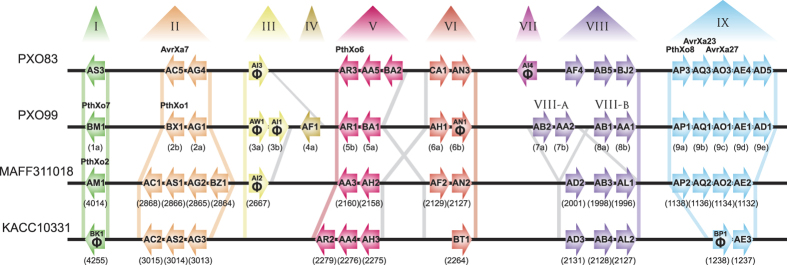
Distribution of *TALE* genes in *Xoo* strains PXO83, PXO99^A^, MAFF311018 and KACC10331. Individual *TALE* genes are represented by arrows, which indicate the orientation of the gene. TALEs are labelled with their new name inside the arrow. Below the arrows, former names or gene numbers are shown in brackets. TALEs with prominent TALEs in their class are labelled in bold above the arrows. Pseudogenes are indicated with the Greek letter phi (Φ). Novel assigned *TALE* clusters (T-I to T-IX) are depicted above the scheme in roman numbers. Similar colors indicate which cluster the TALEs belong to. Colored bars between the genomes represent the regions on which basis the clusters are defined. *TALE* clusters T-IV and T-VII are unique for their respective genome. Grey bars represent chosen similar genomic genes in different genomes that were not used to discriminate *TALE* clusters.

*TALE* genes are not scattered randomly in bacterial genomes, but occur instead at somewhat conserved loci^28^. These loci have previously been numbered, but with a different numbering scheme for different *Xoo* genomes^28^. To simplify phylogenetic comparisons, we propose renaming TALE loci (Fig. 5). Starting with the first sequenced *Xoo* genome of strain MAFF311018, and then with strains KACC10331 and PXO99^A^, we named the TALE loci with “T”-followed by roman numbers (Table 3). The same locus name in different genomes is assigned if at least one of the flanking genes (ignoring mobile elements) is conserved. These flanking genes of *TALE* loci are listed in Table 4. The *TALE* pseudogenes in cluster T-III and T-VII group together into the TALE class AI (Supplementary Fig. S11b) which indicates that they are of common origin, and that genomic rearrangements have re-located them to different sites in the *Xoo* genome. The TALE cluster comparisons (Fig. 5) reveal that some clusters contain different TALEs at the same conserved location in the genome (e.g. cluster T-I; TALE PthXo7 in strain PXO99^A^ and TALE PthXo2 in MAFF31101; Fig. 5 and Table 4) a feature that has been noted earlier^28^. The order of *TALE* genes in other loci (e.g. cluster T-IX) is highly similar, but the number of *TALE* genes differs due to deletions or insertions.

The contribution of mobile elements to type III-effector mobility and recombination is well known^42^,^43^,^44^,^45^,^46^ and apparently also contributes to TALE diversification^40^. Future work will reveal the molecular mechanisms and selective pressures that shape TALE repertoires in *Xanthomonas* spp. genomes.

## Conclusion

Here, we describe an improved way to identify *TALE* genes in genome sequences, and to group TALE proteins into classes. The classification is based on the TALE repeat region, and reflects similar DNA-binding specificities. TALEs within the same class are likely to induce expression of the same target genes in the host plant. The classification provides a quick and convenient way to assess possible functions of TALEs in novel genomes. In parallel, we propose an urgently needed nomenclature for TALEs that reflects these functional relationships. This will prove useful for classifying the large number of TALEs^47^ in *Xanthomonas* spp. genomes that we envision will be published in the future.

## Methods

### Bacterial growth and inoculations

*Xanthomonas oryzae* pv. *oryzae* (*Xoo*) strains were cultivated in PSA medium (10 g peptone, 10 g sucrose, 1 g glutamic acid, 16 g agar per liter H_2_O) at 28 °C. *Oryza sativa* subsp. *japonica* cv. Nipponbare was used for virulence assays. Leaves of 3- to 4-weeks-old plants were infiltrated with bacterial suspensions at an optical density of 0.5 (OD_600_) using a needleless syringe. Symptoms were documented five days post inoculation.

### DNA preparation and Southern blot

Genomic DNA from *Xoo* was isolated using Phenol/Chloroform extraction, digested with *Bam*HI and separated on a 0,8% agarose gel at 90V for 24h. The chemiluminescent detection of genomic *TALE* sequences on Southern blot was done using a digoxigenin-labelled (Roche Applied Science, Mannheim, Germany) probe derived from 500bp of the 3′ part of *talC* from *Xoo* BAI3^39^ that hybridizes to *TALE* genes.

### Next generation sequencing

Libraries were sequenced in five Pacific Biosciences SMRT cells, yielding a total of 163,617 reads with a median read length of 9,669 bp. Reads were assembled in the Pacific Biosciences SMRT Portal using the HGAP_Assembly.3 pipeline. This yielded an initial assembly comprising a single contig of 5,029,672 bases with a coverage of 169.77 and an average concordance of 99.95%. Overlapping ends of this contig were trimmed and the contig was closed to a circular chromosome of 5,025,428 bp. Position 1 of the contig was determined using G/C ratio and the location of genes (*dnaA*, *dnaN*, *gyrB*) typically located close to the origin of replication, such that *dnaA* is located at position 45 in forward orientation relative to the origin.

### Automated annotation of *TALE* genes

For the automatic annotation of *TALE* genes, genomic DNA sequences of known TALEs from fully sequenced *Xoo* (PXO99^A^, MAFF311018, KACC10331) and *Xoc* (BLS256) strains were collected and each split into the regions coding for i) N-terminus, ii) tandem repeats that are part of the binding domain, and iii) C-terminus. For each of these regions, separate hidden Markov models were learnt using HMMer^48^ based on their DNA sequences. Domain occurrences were detected using custom Java code to avoid dependencies on HMMer, allowing for downstream analyses in one stand-alone application. Since arrays of tandem repeats are a unique feature of *TALE* genes, genomes were first scanned for contiguous arrays of matches to the HMM for these repeats. For each array detected, upstream and downstream regions were scanned for matches to N-terminus and C-terminus, respectively, yielding an initial region for each putative *TALE* gene. Initial regions were further refined by i) choosing the longest open reading frame within this region and ii) adjusting start and end of the prediction to the closest start and stop codon within the reading frame, respectively. The annotation of truncated *TALE* pseudogenes, e.g., due to premature stop codons or frame shift mutations, is not straightforward. Here, a conservative approach was chosen, reporting the longest reading frame between ATG and a Stop codon in the initial region. For in-depth analyses of pseudogenes, AnnoTALE provides the full-length DNA sequence of the initial region and its translation in all three frames as an additional file.

### Automated domain splitting of TALEs

From a multiple alignment^49^ of the protein sequences of N-terminal and C-terminal regions, the full tandem repeat, and the last half repeat, the corresponding consensus sequences were extracted as the most frequent AA in each column of the alignment. If in one column the gap symbol was more frequent than any of the AAs, this column was removed from the consensus. Extracted genomic TALE DNA sequences (see above) were translated to the corresponding protein sequences *in silico*. As reference, prototypic TALE protein sequences were built by concatenating the consensus of N-terminus, k tandem repeats, the last half repeat and the C-terminus. For each number k of tandem repeats, a pairwise alignment between the protein sequences of the given TALE and the prototypic TALE sequence was performed (BLOSUM62 substitution matrix^50^, affine gap costs: gap open penalty 3, gap extension penalty 2). That k yielding the best alignment score was chosen. The TALE sequence was then split into those parts induced by the alignment, e.g., the DNA sequence coding for the third repeat of a TALE was extracted as those nucleotides that code for the amino acids aligned to the third repeat in the prototypic TALE sequence. The split repeat sequences were the input of the automated extraction of RVDs in the next step.

### Automated extraction of RVDs

Given one repeat sequence extracted in the previous step, the corresponding protein sequence was aligned to the consensus repeat. In the consensus repeat, the RVD comprises AAs 12 and 13. Hence, those AAs in the given repeat sequence that aligned to those AAs were extracted. For this alignment, a BLOSUM62 substitution matrix and affine gap costs with gap open penalty of 3 and gap extension penalty of 1 were used, to account for the existence of repeat of aberrant lengths^35^.

### Computing p-values of pairwise TALE alignments/distances

Conceptually, the p-value of a TALE distance (resulting from pairwise alignment) was determined as the probability of finding the same or a lower distance by comparing a TALE against a database of random TALE sequences. For the random data base, it was assumed that TALEs are generated by independently drawing a sequence of RVDs with probabilities given by the relative frequencies of RVDs in the input data set. Since the number of possible RVD sequences grows exponentially with the length L, a naive enumeration of TALE is intractable, while a random sampling procedure would only yield approximate and varying p-values. Hence, this problem was approached algorithmically by exploiting two specifics of the given setup: Consider one position in the alignment with RVD ‘ab’ in the TALE of interest. For all possible aligned RVDs ‘de’, we can observe only four different alignment scores: 0 in case of a match, i.e., ‘ab’=‘de’, 0.2 in case of a mismatch only at position 12, 0.8 for a mismatch only at position 13, and 1.0 for a mismatch at both positions. Hence, all RVDs can be grouped by their corresponding alignment scores and the relative frequencies of all RVDs with identical alignment scores were summed up. Second different alignments can be joined. For instance, all alignments with one RVD with a mismatch at AA 12 and one RVD with a mismatch at AA 13 (at the same or any other combination of RVDs) and all alignments with 5 RVDs having mismatches only at AA 12 yield the same pairwise distance of 1.0 and their probabilities can be joined. These aggregations on the RVD and the alignment level are combined in a dynamic programming-like approach to yield exact p-values in a runtime roughly linear (instead of exponential) in the number of RVD positions in the alignment.

### Building classes of TALEs

Classes of TALEs were built such that within each class the average pairwise distance between member TALEs did not exceed a given threshold T. More specifically, agglomerative hierarchical clustering with average linkage (UPGMA^51^) was used to obtain a tree of all TALEs. Classes were then determined by cutting the resulting tree at height T, yielding a number of sub-trees, each constituting a TALE class. This process and, hence, the number and composition of resulting TALE classes, depends on the choice of the threshold T.

To find a reliable threshold, we developed a significance measure (see above) that assigns each pairwise alignment and, subsequently, each given class of TALEs a p-value. This p-value reflects the probability that any pair of TALEs is assigned to a common class just by chance. We then test a wide range of thresholds (Supplementary Fig. S12a) for the assignment of TALEs of the initial set (Supplementary Table S2) into classes. We find that all class assignments obtain significant p-values (α = 0.01) up to a threshold of 5.5 (p = 2.9 × 10^−5^), while the number of classes C continuously decreases with increasing threshold. Since the p-value dramatically increases for greater thresholds (p = 0.065 for T = 5.6), we finally chose a threshold of T = 5.0 yielding C = 52 classes for the initial set of 83 TALEs (instead of C = 51 for T = 5.5,). The majority of these classes contains one or two TALEs, but we also observe classes of up to 6 members in the final set of classes (Supplementary Fig. S12b).

## Additional Information

**Accession codes:** The genome sequence of *Xoo* PXO83 can be found at Genbank accession number CP012947. The program suite and a user guide for the suite can be downloaded from http://www.jstacs.de/index.php/AnnoTALE.

**How to cite this article**: Grau, J. *et al.* AnnoTALE: bioinformatics tools for identification, annotation, and nomenclature of TALEs from *Xanthomonas* genomic sequences. *Sci. Rep.* **6**, 21077; doi: 10.1038/srep21077 (2016).

## Supplementary Material

Supplementary Information

## Figures and Tables

**Table 1:** *Xanthomonas oryzae* ov. *oryzae* strains used in this study^a^.

Strain	Race type	RFLP type	Date of sampling	Place of sampling
PXO35	1	3	1972	Culape, Lucban, Quezon
PXO132	1	2	1979	Buguey, Cagayan
PXO83	2	11	1976	Nueva Ecija
PXO142	3	14	1981	Panabo, Davao
PXO143	3	16	1982	Lopez, Bohol
PXO183	5	25	1986	Banaue, Ifugao
PXO99	6	27	1980	Los Baños, Laguna

^a^All strains originate from the Philippines, details from Leach *et al.*^31^

**Table 2:** *Xoo* PXO83 genomic parameters compared to other *Xoo* genomes.

	PXO83	PXO99^A^	MAFF311018	KACC10331
Length (bp)	5,025,428	5,240,074	4,940,217	4,941,439
GC content (%)	63.7	63.6	63.7	63.7
*TALE* genes^a^	18	19	17	13
PacBio sequence	+	–	–	–
PacBio coverage	169.77x	–	–	–

^a^–*TALE* genes, including pseudogenes.

**Table 3:** *Xoo* PXO83 TALEs.

TALE name	repeats	RVDs^a^
TalAS3	26.5	NI-HG-NI-NI-HG-HD-NN-HD-HD-HD-NI-NI-NN-NI-HD-HD-HD-HG-NN-NN-HD-NS-NN-HD-N*-NS-N*
TalAC5	25.5	NI-HG-NI-NI-NS-HD-NN-HD-HD-HD-NS-N*-N*-HD-HD-NS-NS-NN-NN-NI-NG-NN-NI-N*-NS-N*
TalAG4	19.5	NI-NG-NN-NG-NK-NG-NI-NN-NI-NN-NI-NN-NS-NG-NS-NN-NI-N*-NS-NG
ΦTalAI4	17.5	NS-NG-NG-NG-NG-NG-NG-HD-HD-HD-NN-HD-NG-HD-HD-HD-HD-H*
TalAR3	22.5	NI-H*-NI-NN-NN-NN-NN-NN-HD-NI-NN-HG-HD-NI-N*-NS-NI-NI-HD-N*-NS-NI-NG
TalAA5	19.5	NI-HG-NS-HG-HG-HD-NS-NG-HD-NN-NG-HG-NG-HD-HG-HD-HD-NI-NN-NG
TalBA2	15.5	NI-NS-HD-HG-NS-NN-HD-H*-NG-NN-NN-HD-HD-NG-HD-NG
TalCA1	16.5	NI-N*-NI-NS-NN-NG-NN-HD-HD-HD-NG-HD-NS-HD-N*-NS-NG
TalAN3	20.5	NI-N*-NS-HG-NI-NI-NS-HD-NN-HD-NS-NG-SS-HD-NI-NI-NN-NI-NN-NI-NG
ΦTalAI3	17.5	NS-HD-NG-NG-NG-NG-NG-HD-HD-HD-NN-HD-NG-HD-NI-HD-NN-N*
TalAF4	15.5	NI-NN-NN-NI-NI-NI-HD-NS-HG-NN-NN-NN-NI-NI-NG-HD
TalAB5	17.5	NI-HG-NI-NI-NI-NN-HD-NS-NN-NS-NN-HD-NN-NI-HD-NN-NS-NG
TalBJ2	15.5	NI-H*-NI-HG-NI-NI-NN-HD-NI-HD-NN-HG-NS-N*-HD-N*
TalAP3	19.5	HD-HD-HD-NG-N*-NN-HD-HD-N*-NI-NI-NN-HD-HI-ND-HD-NI-HD-NG-NG
TalAQ3	26.5	HD-HD-NN-NN-NS-NG-HD-S*-HG-HD-NG-N*-HD-HD-HD-N*-NN-NI-NN-HD-HI-ND-HD-HG-NN-HG-N*
TalAO3	16.5	NI-NN-N*-NG-NS-NN-NN-NN-NI-NN-NI-N*-HD-HD-NI-NG-NG
TalAE4	12.5	NI-NN-NI-HG-HG-NV-HG-HD-HG-HD-HD-HD-NG
TalAD5	23.5	NN-HD-NS-NG-HD-NN-N*-NI-HD-NS-HD-NN-HD-NN-HD-NN-NN-NN-NN-NN-NN-NN-HD-NG

^a^RVD: repeat variable di-residue.

**Table 4:** Definition of genomic *TALE* loci.

TALE loci	flanking gene 5′^a^	flanking gene 3′^b^
T-I	lipase family CDS (protein ID: ACD57201.1)	orfB CDS (protein ID: ACD57196.1)
T-II	tetratricopeptide repeat domain protein CDS (protein ID: ACD58247.1)	RND superfamily protein CDS (protein ID: ACD58240.1)
T-III	TonB-dependent receptor CDS (protein ID: ACD58443.1)	–c
T-IV	–d	–d
T-V	–e	glyoxalase family protein CDS (protein ID: ACD58912.1)
T-VI	–e	phosphoribosyl-AMP cyclohydrolase domain protein CDS (protein ID: ACD58954.1)
T-VII	–d	–d
T-VIII	–f	serS CDS (protein ID: ACD59234.1)
T-IX	Uroporphyrinogen decarboxylase HemE (protein ID:ACD60555.1)	TonB-dependent outer membrane Receptor (protein ID: ACD60577.1)

^a^next gene, which is not a mobile element or transposon, 5′ of the ATG of the *TALE* gene.

^b^next gene, which is not a mobile element or transposon, 3′ of the stop codon of the *TALE* gene.

^c^not the same gene, in different *Xoo* strains, is flanking the cluster at the 3′ site.

^d^Through recombination between genomic regions not possible to define the cluster by flanking genes.

^e^Because of an inversion, between PXO-strains and MAFF311018/KACC10331 strains, the flanking genes at the 5′ site can be different.

^f^not the same gene, in different *Xoo* strains, is flanking the cluster at the 5′ site.

## References

[b1] Boch J. *et al.* Breaking the code of DNA binding specificity of TAL-type III effectors. Science 326, 1509–1512 (2009). 19933107 10.1126/science.1178811

[b2] Moscou M. J. & Bogdanove A. J. A simple cipher governs DNA recognition by TAL effectors. Science 326, 1501 (2009). 19933106 10.1126/science.1178817

[b3] Deng D. *et al.* Structural basis for sequence-specific recognition of DNA by TAL effectors. Science 335, 720–723 (2012). 22223738 10.1126/science.1215670 PMC3586824

[b4] Mak A. N., Bradley P., Cernadas R. A., Bogdanove A. J. & Stoddard B. L. The crystal structure of TAL effector PthXo1 bound to its DNA target. Science 335, 716–719 (2012). 22223736 10.1126/science.1216211 PMC3427646

[b5] Boch J. & Bonas U. *Xanthomonas* AvrBs3 family-type III effectors: discovery and function. Annu Rev Phytopathol 48, 419–436 (2010). 19400638 10.1146/annurev-phyto-080508-081936

[b6] Cong L., Zhou R., Kuo Y. C., Cunniff M. & Zhang F. Comprehensive interrogation of natural TALE DNA-binding modules and transcriptional repressor domains. Nat Commun 3, 968 (2012). 22828628 10.1038/ncomms1962 PMC3556390

[b7] Juillerat A. *et al.* Optimized tuning of TALEN specificity using non-conventional RVDs. Sci Rep 5, 8150 (2015). 25632877 10.1038/srep08150 PMC4311247

[b8] Miller J. C. *et al.* Improved specificity of TALE-based genome editing using an expanded RVD repertoire. Nat Methods 12, 465–471 (2015). 25799440 10.1038/nmeth.3330

[b9] Streubel J., Blücher C., Landgraf A. & Boch J. TAL effector RVD specificities and efficiencies. Nat Biotechnol 30, 593–595 (2012). 22781676 10.1038/nbt.2304

[b10] Yang J. *et al.* Complete decoding of TAL effectors for DNA recognition. Cell Res 24, 628–631 (2014). 24513857 10.1038/cr.2014.19 PMC4011339

[b11] Cuculis L., Abil Z., Zhao H. & Schroeder C. M. Direct observation of TALE protein dynamics reveals a two-state search mechanism. Nat Commun 6, 7277 (2015). 26027871 10.1038/ncomms8277 PMC4458887

[b12] Gao H., Wu X., Chai J. & Han Z. Crystal structure of a TALE protein reveals an extended N-terminal DNA binding region. Cell Res 22, 1716–1720 (2012). 23147789 10.1038/cr.2012.156 PMC3515758

[b13] Schreiber T. & Bonas U. Repeat 1 of TAL effectors affects target specificity for the base at position zero. Nucleic Acids Res 42, 7160–7169 (2014). 24792160 10.1093/nar/gku341 PMC4066769

[b14] Bogdanove A. J. & Voytas D. F. TAL effectors: customizable proteins for DNA targeting. Science 333, 1843–1846 (2011). 21960622 10.1126/science.1204094

[b15] de Lange O., Binder A. & Lahaye T. From dead leaf, to new life: TAL effectors as tools for synthetic biology. Plant J 78, 753–771 (2014). 24602153 10.1111/tpj.12431

[b16] Zhang H. & Wang S. Rice versus *Xanthomonas oryzae* pv. *oryzae*: a unique pathosystem. Curr Opin Plant Biol 16, 188–195 (2013). 23466254 10.1016/j.pbi.2013.02.008

[b17] Gonzalez C. *et al.* Molecular and pathotypic characterization of new *Xanthomonas oryzae* strains from West Africa. Mol Plant-Microbe Interact 20, 534–546 (2007). 17506331 10.1094/MPMI-20-5-0534

[b18] Triplett L., Koebnik R., Verdier V. & Leach J. E. In Genomics of plant-associated bacteria. (ed. Gross D. C., Ann Lichens-Park, Chittaranjan Kole ) 127–150 (Springer-Verlag, Berlin Heidelberg; 2014).

[b19] Triplett L. R. *et al.* Genomic analysis of *Xanthomonas oryzae* isolates from rice grown in the United States reveals substantial divergence from known *X. oryzae* pathovars. Appl Environ Microbiol 77, 3930–3937 (2011). 21515727 10.1128/AEM.00028-11 PMC3131649

[b20] Yang B. & White F. F. Diverse members of the AvrBs3/PthA family of type III effectors are major virulence determinants in bacterial blight disease of rice. Mol Plant-Microbe Interact 17, 1192–1200 (2004). 15553245 10.1094/MPMI.2004.17.11.1192

[b21] Boch J., Bonas U. & Lahaye T. TAL effectors - pathogen strategies and plant resistance engineering. New Phytol 204, 823–832 (2014). 25539004 10.1111/nph.13015

[b22] Doyle E. L. *et al.* TAL Effector-Nucleotide Targeter (TALE-NT) 2.0: tools for TAL effector design and target prediction. Nucleic Acids Res 40, W117–122 (2012). 22693217 10.1093/nar/gks608 PMC3394250

[b23] Grau J. *et al.* Computational predictions provide insights into the biology of TAL effector target sites. PLoS Comput Biol 9, e1002962 (2013). 23526890 10.1371/journal.pcbi.1002962 PMC3597551

[b24] Pérez-Quintero A. L. *et al.* An improved method for TAL effectors DNA-binding sites prediction reveals functional convergence in TAL repertoires of *Xanthomonas oryzae* strains. PLoS ONE 8, e68464 (2013). 23869221 10.1371/journal.pone.0068464 PMC3711819

[b25] Bogdanove A. J. *et al.* Two new complete genome sequences offer insight into host and tissue specificity of plant pathogenic Xanthomonas spp. J Bacteriol 193, 5450–5464 (2011). 21784931 10.1128/JB.05262-11 PMC3187462

[b26] Lee B. M. *et al.* The genome sequence of *Xanthomonas oryzae* pathovar *oryzae* KACC10331, the bacterial blight pathogen of rice. Nucleic Acids Res 33, 577–586 (2005). 15673718 10.1093/nar/gki206 PMC548351

[b27] Ochiai H., Inoue Y., Takeya M., Sasaki A. & Kaku H. Genome sequence of *Xanthomonas oryzae* pv. *oryzae* suggests contribution of large numbers of effector genes and insertion sequences to its race diversity. Jpn Agr Res Q 39, 275–287 (2005).

[b28] Salzberg S. L. *et al.* Genome sequence and rapid evolution of the rice pathogen *Xanthomonas oryzae* pv. *oryzae* PXO99A. BMC Genomics 9, 204 (2008). 18452608 10.1186/1471-2164-9-204 PMC2432079

[b29] Pesce C. *et al.* High-Quality Draft Genome Sequence of the Xanthomonas translucens pv. cerealis Pathotype Strain CFBP 2541. Genome Announcements 3 (2015). 10.1128/genomeA.01574-14 PMC4333671 25676771

[b30] Eid J. *et al.* Real-time DNA sequencing from single polymerase molecules. Science 323, 133–138 (2009). 19023044 10.1126/science.1162986

[b31] Leach J. E. *et al.* Assessment of genetic diversity and population structure of *Xanthomonas oryzae* pv. *oryzae* with a repetitive DNA element. Appl Environ Microbiol 58, 2188–2195 (1992). 1353345 10.1128/aem.58.7.2188-2195.1992 PMC195754

[b32] Mew T. W. & Vera Cruz C. M. Variability of *Xanthomonas oryzae*: specificity in infection of rice differentials. Phytopathology 69, 152–155 (1979).

[b33] Darling A. E., Mau B. & Perna N. T. progressiveMauve: multiple genome alignment with gene gain, loss and rearrangement. PLoS ONE 5, e11147 (2010). 20593022 10.1371/journal.pone.0011147 PMC2892488

[b34] Grau J. *et al.* Jstacs: a Java framework for statistical analysis and classification of biological sequences. Journal of Machine Learning Research 13, 1967–1971 (2012).

[b35] Richter A. *et al.* A TAL effector repeat architecture for frame shift binding. Nat Commun 5, 3447 (2014). 24614980 10.1038/ncomms4447

[b36] Booher N. J. *et al.* Single molecule real-time sequencing of Xanthomonas oryzae genomes reveals a dynamic structure and complex TAL (transcription activator-like) effector gene relationships. Microb Genom 1, 10.1099/mgen.0.000032 (2015). PMC4853030 27148456

[b37] Perez-Quintero A. L. *et al.* QueTAL: a suite of tools to classify and compare TAL effectors functionally and phylogenetically. Front Plant Sci 6, 545 (2015). 26284082 10.3389/fpls.2015.00545 PMC4522561

[b38] Zhou J. *et al.* Gene targeting by the TAL effector PthXo2 reveals cryptic resistance gene for bacterial blight of rice. Plant J 82, 632–643 (2015). 25824104 10.1111/tpj.12838

[b39] Yu Y. *et al.* Colonization of rice leaf blades by an african strain of *Xanthomonas oryzae* pv. *oryzae* depends on a new TAL effector that induces the rice Nodulin-3 *Os11N3* Gene. Mol Plant-Microbe Interact 24, 1102–1113 (2011). 21679014 10.1094/MPMI-11-10-0254

[b40] Ferreira R. M. *et al.* A TALE of transposition: Tn3-like transposons play a major role in the spread of pathogenicity determinants of *Xanthomonas citri* and other xanthomonads. mBio 6, e02505–02514 (2015). 25691597 10.1128/mBio.02505-14 PMC4337579

[b41] Grau J., Boch J. & Posch S. TALEN offer: genome-wide TALEN off-target prediction. Bioinformatics 29, 2931–2932 (2013). 23995255 10.1093/bioinformatics/btt501

[b42] Landgraf A., Weingart H., Tsiamis G. & Boch J. Different versions of *Pseudomonas syringae* pv. *tomato* DC3000 exist due to the activity of an effector transposon. Mol Plant Pathol 7, 355–364 (2006). 20507452 10.1111/j.1364-3703.2006.00343.x

[b43] Noël L., Thieme F., Gäbler J., Büttner D. & Bonas U. XopC and XopJ, two novel type III effector proteins from *Xanthomonas campestris* pv. vesicatoria. J Bacteriol 185, 7092–7102 (2003). 14645268 10.1128/JB.185.24.7092-7102.2003 PMC296255

[b44] Rivas L. A., Mansfield J., Tsiamis G., Jackson R. W. & Murillo J. Changes in race-specific virulence in *Pseudomonas syringae* pv. *phaseolicola* are associated with a chimeric transposable element and rare deletion events in a plasmid-borne pathogenicity island. Appl Environ Microbiol 71, 3778–3785 (2005). 16000789 10.1128/AEM.71.7.3778-3785.2005 PMC1169007

[b45] Rohmer L., Guttman D. S. & Dangl J. L. Diverse evolutionary mechanisms shape the type III effector virulence factor repertoire in the plant pathogen *Pseudomonas syringae*. Genetics 167, 1341–1360 (2004). 15280247 10.1534/genetics.103.019638 PMC1470954

[b46] Stavrinides J., Ma W. & Guttman D. S. Terminal reassortment drives the quantum evolution of type III effectors in bacterial pathogens. PLoS Pathog 2, e104 (2006). 17040127 10.1371/journal.ppat.0020104 PMC1599762

[b47] Wilkins K. E., Booher N. J., Wang L. & Bogdanove A. J. TAL effectors and activation of predicted host targets distinguish Asian from African strains of the rice pathogen *Xanthomonas oryzae* pv. *oryzicola* while strict conservation suggests universal importance of five TAL effectors. *Front* Plant Sci 6, 536 (2015). 10.3389/fpls.2015.00536 PMC4508525 26257749

[b48] Wheeler T. J. & Eddy S. R. nhmmer: DNA homology search with profile HMMs. Bioinformatics 29, 2487–2489 (2013). 23842809 10.1093/bioinformatics/btt403 PMC3777106

[b49] Sievers F. *et al.* Fast, scalable generation of high-quality protein multiple sequence alignments using Clustal Omega. Mol Syst Biol 7, 539 (2011). 21988835 10.1038/msb.2011.75 PMC3261699

[b50] Henikoff S. & Henikoff J. G. Amino acid substitution matrices from protein blocks. Proc Natl Acad Sci USA 89, 10915–10919. 1438297 10.1073/pnas.89.22.10915 PMC50453

[b51] Sokal R. R. & Michener C. D. A statistical method of evaluating systematic relationships. U Kansas Sci Bull 28, 1409–1438 (1958).

