# Supplementary material for: AnnoTALE: bioinformatics tools for identification, annotation, and nomenclature of TALEs from Xanthomonas genomic sequences
Source: Sci Rep. 2016 Feb 15;6:21077. doi: 10.1038/srep21077 (PMC4753510; doi:10.1038/srep21077)
Supplement: Supplementary Information [file srep21077-s1.pdf]

Supplementary information for:

AnnoTALE: bioinformatics tools for identification, annotation, and nomenclature of TALEs from *Xanthomonas* genomic sequences

Jan Grau[1]*, Maik Reschke[2, 3], Annett Erkes[1], Jana Streubel[2, 3], Richard D. Morgan[4], Geoffrey G. Wilson[4], Ralf Koebnik[5], Jens Boch[2, 3]*

Affiliations:

[1]Institute of Computer Science, Martin Luther University Halle-Wittenberg, Halle (Saale), Germany.

[2]Department of Genetics, Martin Luther University Halle-Wittenberg, Weinbergweg 10, D-06120 Halle (Saale), Germany.

[3]Department of Plant Biotechnology, Leibniz University Hannover, Herrenhäuser Str. 2, D-30419 Hannover, Germany.

[4]New England Biolabs Inc., 240 Country Road, Ipswich, MA 01938, USA.

[5]UMR 186 IRD-UM2-Cirad "Résistance des Plantes aux Bioagresseurs", BP 64501, 34394 Montpellier cedex 5, France.

*Correspondence to: jens.boch@genetik.uni-hannover.de
or
grau@informatik.uni-halle.de

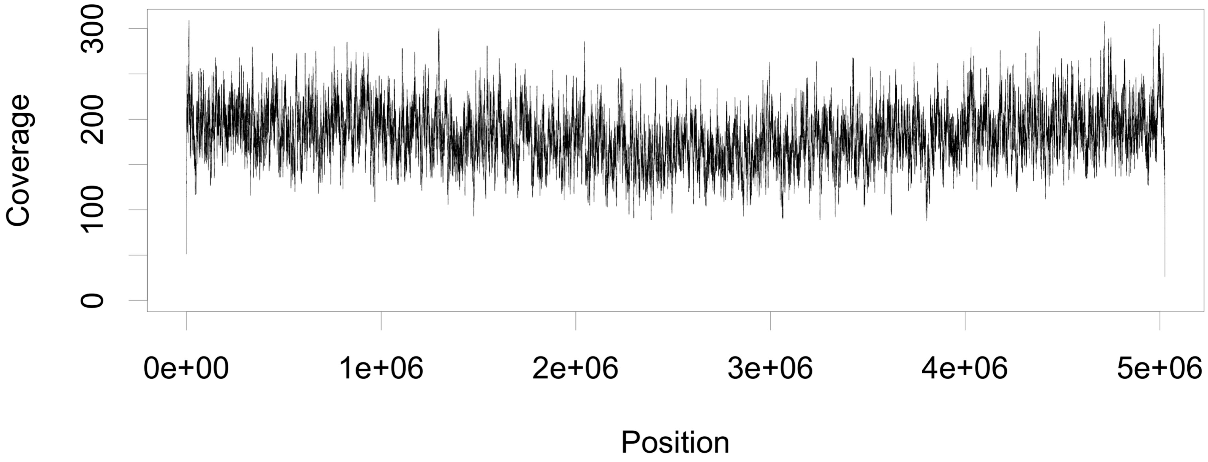

Supplementary Figure S1 | Coverage profile of *Xoo* PXO83 chromosome. We map PacBio reads against the final *Xoo* PXO83 chromosome sequence in a resequencing experiment using the PacBio SMRT Portal software. We plot the coverage with PacBio reads against the genomic positions ignoring reads with a mapping quality of zero. Except for bordering positions, we find a stable coverage varying around the mean resequencing coverage of approximately 182.

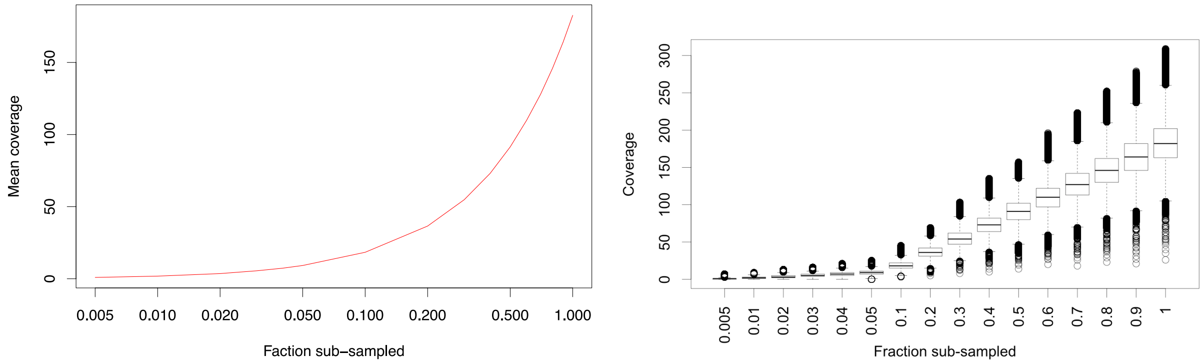

Supplementary Figure S2 | Coverage for different sub-samples of PacBio reads. We map PacBio reads against the final *Xoo* PXO83 chromosome sequence in a resequencing experiment using the PacBio SMRT Portal software. We then sub-sample fractions of 0.005 to 1.0 of the mapped PacBio reads and compute the coverage values for all genomic positions using only the sub-sampled reads. Left: We plot the mean coverage against the fraction of sub-sampled reads. As expected, the mean coverage scales approximately linearly with the fraction of sub-sampled reads. For instance, we obtain a mean coverage of 18.3 for a sub-sample containing 10% of the original PacBio reads compared to the original mean coverage of approximately 182.7 using all reads. Right: We also create boxplots of the corresponding coverage values for each of the sub-sampled sets of PacBio reads. As expected, the median coverage scales approximately linearly with the fraction of sub-sampled reads. For instance, we obtain a median coverage of 18 for a sub-sample containing 10% of the original PacBio reads compared to the original median coverage of 182 using all reads.

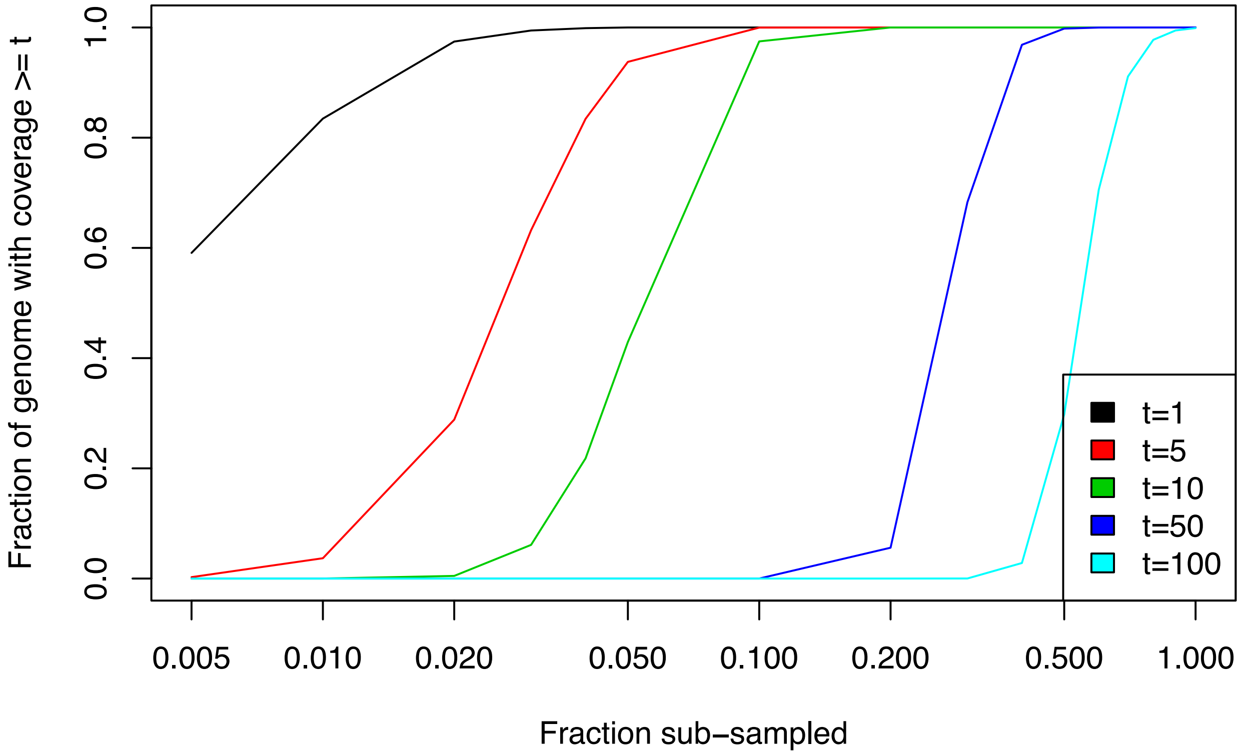

Supplementary Figure S3 | Minimum coverage of genomic regions for different sub-samples of PacBio reads. We map PacBio reads against the final *Xoo* PXO83 chromosome sequence in a resequencing experiment using the PacBio SMRT Portal software. We then sub-sample fractions of 0.005 to 1.0 of the mapped PacBio reads and compute the coverage values for all genomic positions using only the sub-sampled reads. We then compute, for each of the sub-sampled sets of PacBio reads, the fraction of the genome that is covered by at least t reads. We find that for sub-samples containing at least 5% of the PacBio reads, almost all genomic positions (99.998%) are already covered by at least one read. For sub-samples containing at least 20% of the reads, the genome is covered by at least 5 reads. Using all PacBio reads, almost the complete genome (99.89%) is covered by at least 100 PacBio reads.

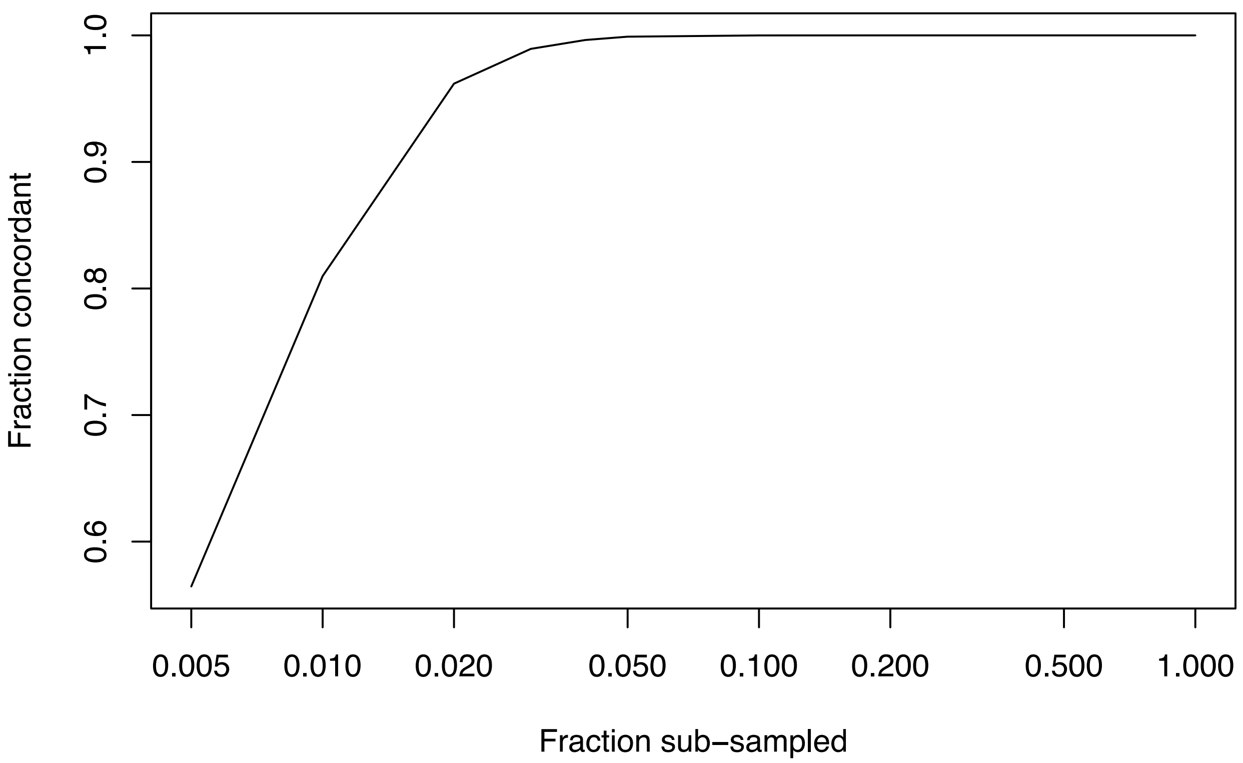

Supplementary Figure S4 | Concordance of base calls for different sub-samples of PacBio reads. We map PacBio reads against the final *Xoo* PXO83 chromosome sequence in a resequencing experiment using the PacBio SMRT Portal software. We then sub-sample fractions of 0.005 to 1.0 of the mapped PacBio reads and compute the coverage values for all genomic positions using only the sub-sampled reads. We then call for each genomic position the corresponding base, an insertion or a deletion, depending on the most frequent event in the covering PacBio reads. We then compare this call to the base on the assembled *Xoo* PXO83 chromosome.

For lower coverages due to sub-sampling, we find substantial deviations from the final *Xoo* PXO83 chromosome. For all sub-samples containing at least 10% of the PacBio reads (concordance of 99.995%), however, we find an almost perfect concordance between these calls and the assembled chromosome, reaching 100% using 30% of the reads. This indicates that a local coverage of at least 20 should be sufficient to make high-confidence base calls, although this coverage might not have been sufficient to yield one closed contig in a de-novo assembly. However, the complete set of PacBio reads corresponds to a coverage of at least 80, except for at most 20 positions at each of the chromosome borders.

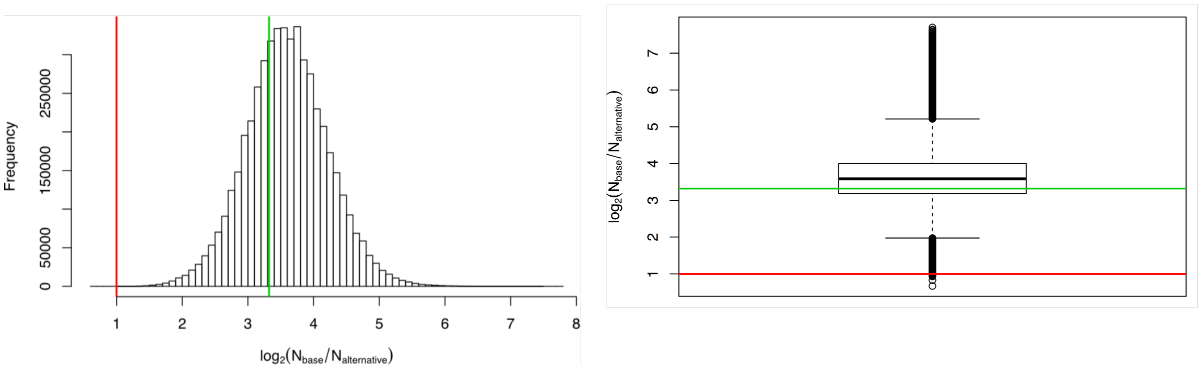

Supplementary Figure S5 | Ambiguity of base calls. We map PacBio reads against the final *Xoo* PXO83 chromosome sequence in a resequencing experiment using the PacBio SMRT Portal software. For each genomic position, we then determine the number of reads supporting the base called in the final assembly of the *Xoo* PXO83 chromosome and compare it to the number of reads supporting the best alternative, i.e., the best alternative base, or an insertion or a deletion at that genomic position. We create a logarithmic histogram (left) and boxplot (right) of the ratios of the number of reads supporting the called base (N_{base}) and the number of reads supporting the best alternative ($N_{alternative}$).

We find that for the large majority (99.9998%) of positions, the called base is supported by at least twice the number of reads as the best alternative, corresponding to a log_2 ratio of 1 (red line). For more than two thirds of the positions, the called base is supported by at least 10-fold the reads compared with the best alternative (green line). This is an additional indication that we may yield a high-confidence genome with the given coverage with PacBio reads.

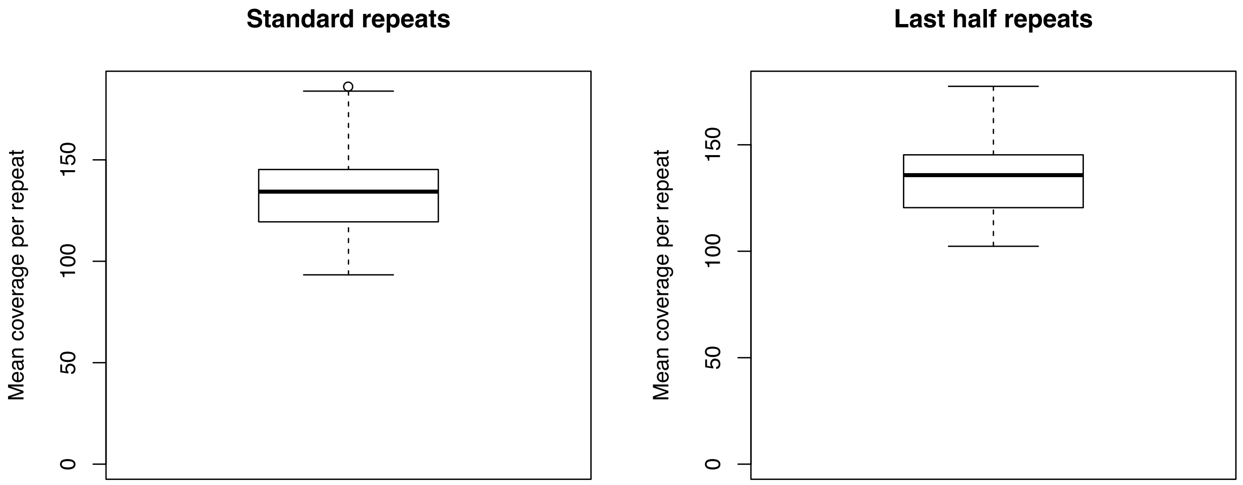

Supplementary Figure S6 | Coverage of TALE repeats. We map PacBio reads against the final *Xoo* PXO83 chromosome sequence in a resequencing experiment using the PacBio SMRT Portal software. We then record the mean coverage values for the genomic locations of each full TALE repeat (left) and each last half repeat (right) of all TALEs predicted on the *Xoo* PXO83 chromosome. For full and last half TALE repeats, we find a median coverage of approximately 134 and 135, respectively, and a minimum coverage of approximately 93 and 102, respectively. Hence, all TALE repeats yield a sufficient coverage to obtain high-confidence base calls (see above).

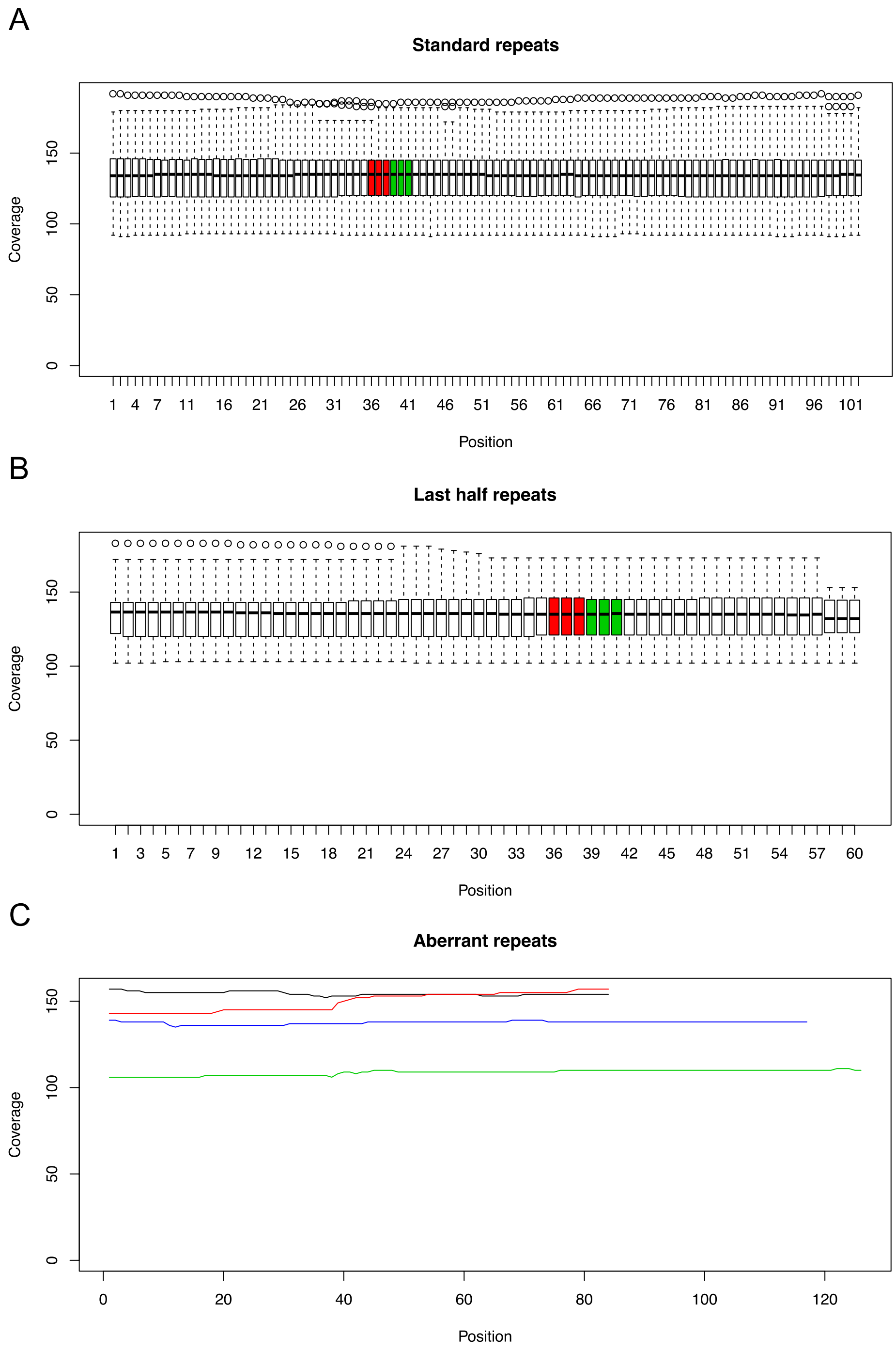

Supplementary Figure S7 | Coverage of TALE repeats. We map PacBio reads against the final *Xoo* PXO83 chromosome sequence in a resequencing experiment using the PacBio SMRT Portal software. We then record the coverage profile for the genomic locations of each TALE repeat of all TALEs predicted on the *Xoo* PXO83 chromosome. For each position in the standard and last half TALE repeats, we create a boxplot of the corresponding coverage values across all repeats (panel A and B). We highlight codons 12 (red) and 13 (green) that code for RVDs. In N*, H*, and S* repeats, the RVD comprises only the AA encoded by codon 12 and positions 100 to 102 (positions 58 to 60 for half repeats) are missing. For each position in the aberrant TALE repeats (panel C), we plot the corresponding coverage profiles. We find a largely uniform coverage across all repeat positions.

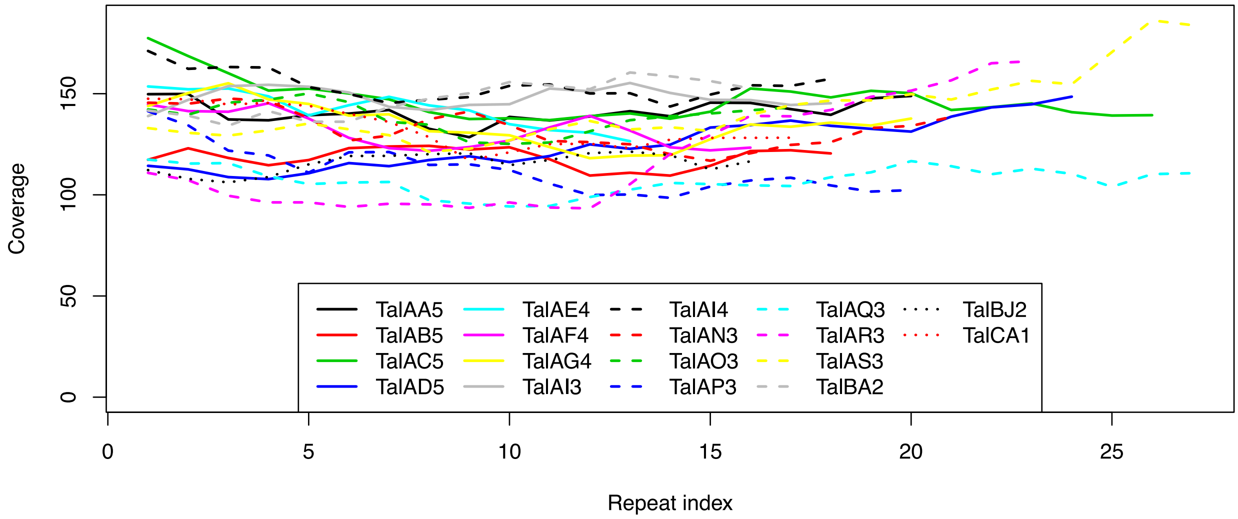

Supplementary Figure S8 | Coverage of TALEs is rather uniform. We map PacBio reads against the final *Xoo* PXO83 chromosome sequence in a resequencing experiment using the PacBio SMRT Portal software. We then record the average coverage for each TALE repeat and plot the mean coverage values of the individual repeats of each TALE in chromosomal order. We find that the coverage differs between different TALEs due to general fluctuations of coverage along the chromosome. However, for each individual TALE, the mean coverage values of the contained repeats are similar and larger fluctuations (e.g., for TalAR3) are not limited to individual repeats but follow general trends. These findings indicate that reads of two different repeats are not erroneously mapped to the same repeat, which would result in approximately a doubling of coverage. They also indicate that reads belonging to a single TALE are not erroneously divided into two repeats, which would result in an abrupt, substantial drop of coverage.

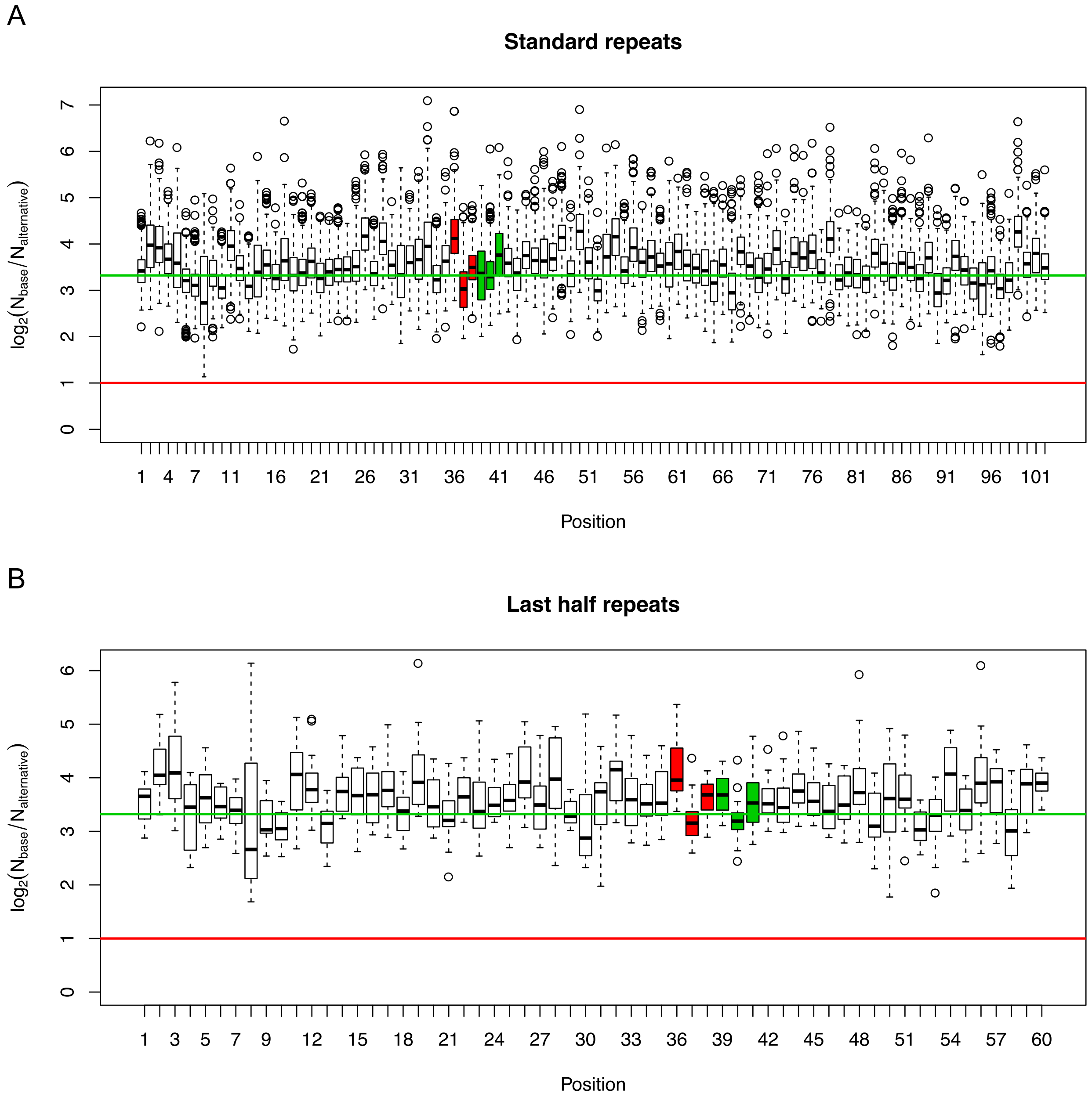

Supplementary Figure S9 | Ambiguity of base calls in TALE repeats. We map PacBio reads against the final *Xoo* PXO83 chromosome sequence in a resequencing experiment using the PacBio SMRT Portal software. For each position of each full, non-aberrant (A) and last half (B) TALE repeat of all TALEs predicted on the *Xoo* PXO83 chromosome, we then determine the number of reads supporting the base called in the final assembly of the *Xoo* PXO83 chromosome and compare it to the number of reads supporting the best alternative, i.e., the best alternative base, or an insertion or a deletion at that genomic position. We create a logarithmic boxplot of the ratios of the number of reads supporting the called base (N_{base}) and the number of reads supporting the best alternative ($N_{alternative}$) for each repeat position. Since TALE repeats are highly conserved on the DNA level and mainly differ in the codon pair coding for the RVD, we would expect a different ratio for these codon pairs (highlighted in red and green) in case of erroneous mappings. However, we do not observe a deviating pattern for the RVD-coding positions, which indicates that the base calls in those codon pairs are reliable.

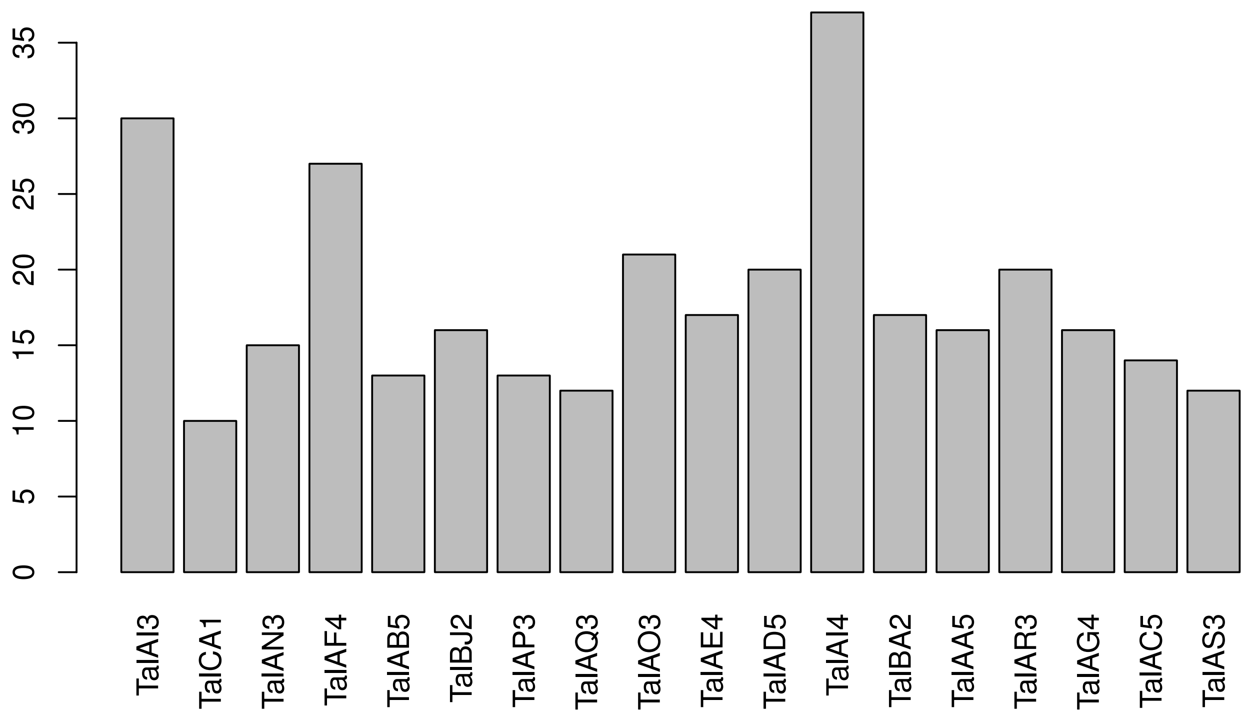

Supplementary Figure S10 | PacBio reads spanning TALEs. We map PacBio reads against the final *Xoo* PXO83 chromosome sequence in a resequencing experiment using the PacBio SMRT Portal software. For a correct assembly of TALE genes, it is important that a sufficient number of PacBio reads spans the complete TALE including some upstream and downstream genomic sequence, because of the repetitive TALE DNA sequence and the high conservation of N-terminal and C-terminal regions. For each of the PXO83 TALEs, we count the number of PacBio reads that span the TALE and additionally at least 100 bp upstream and 100 bp downstream of the TALE sequence. We find that all TALEs are spanned in this manner by at least 10 PacBio reads, which should be sufficient to place shorter or partially overlapping PacBio reads on the TALEs and to correctly place TALEs in the complete chromosome sequence.

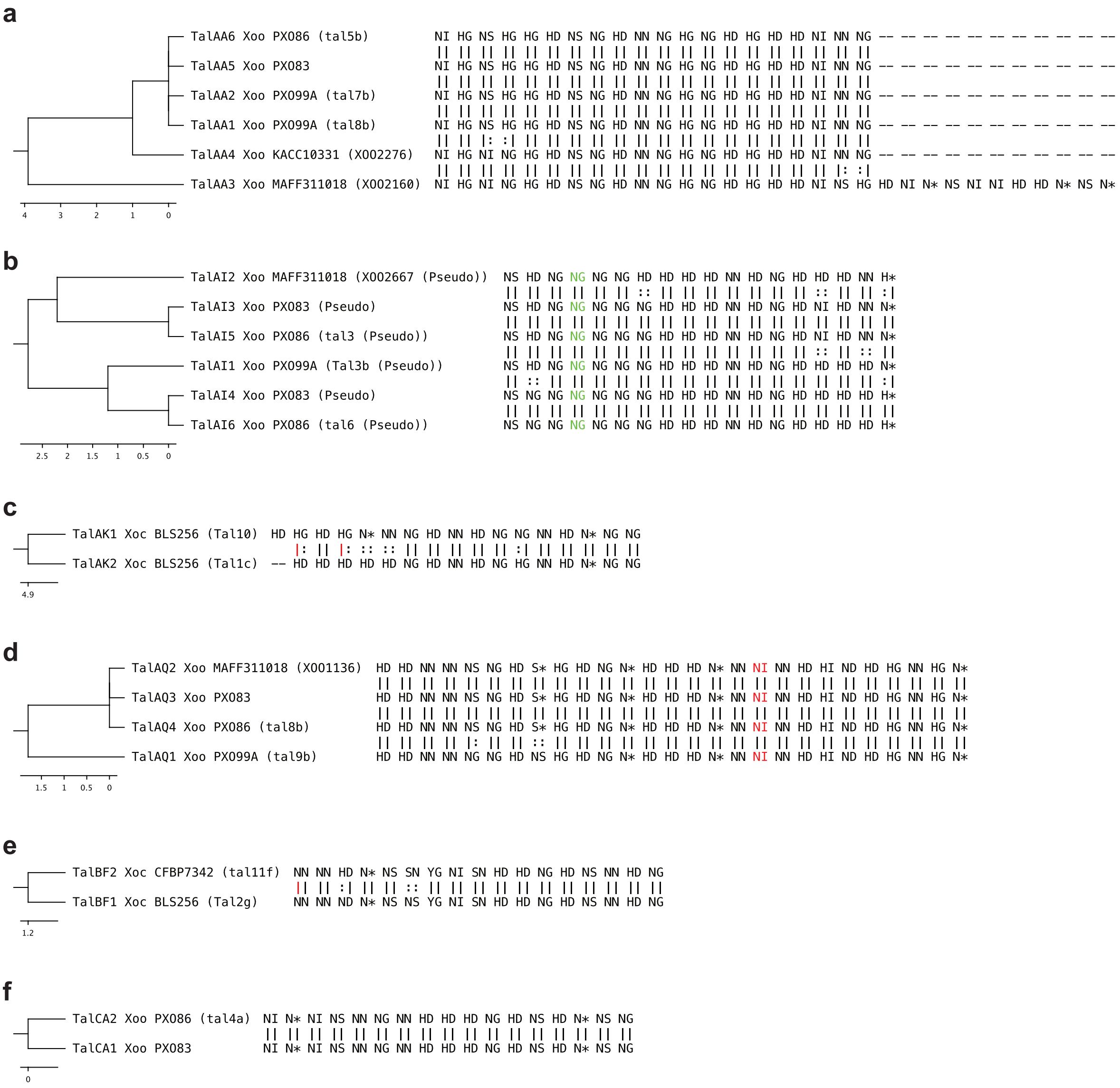

Supplementary Figure S11 | Graphical representation of selected TALE classes. Different TALE classes with aligned RVD sequences are displayed. Identical amino acids (aa) are indicated by black lines. Different aa are indicated by two points between the aa. Red lines between RVDs indicate synonymous substitutions in RVD codons. Abberant short repeats (class AI, NG repeat with 28aa) and abberant long repeats (class AQ, NI repeat with 42aa) are shown in green and red, respectively. (a) Class AA. (b) Class AI. (c) Class AK. (d) Class AQ. (e) Class BF. (f) Class CA.

a

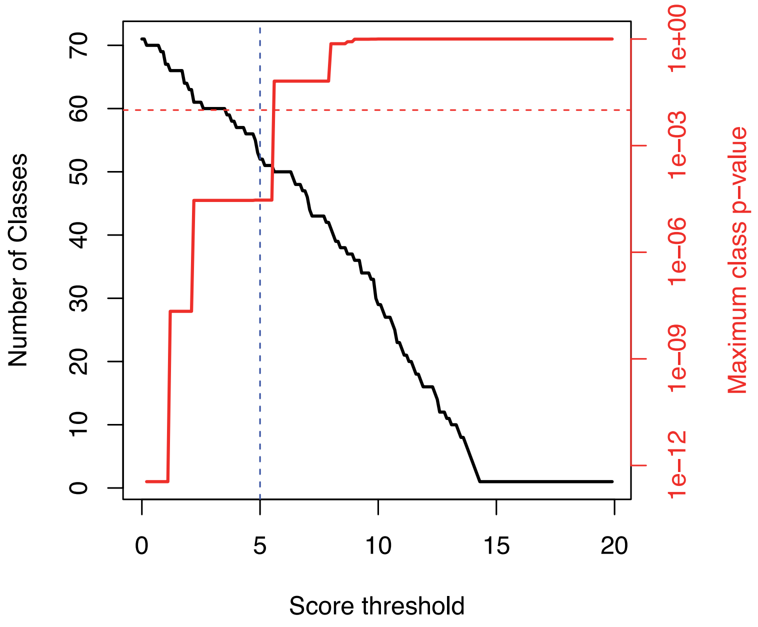

b

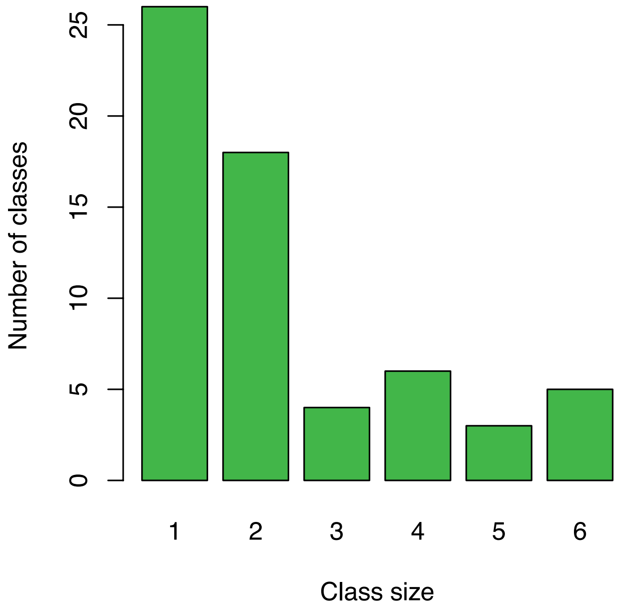

Supplementary Figure S12 | Determination of the threshhold for TALE class assignment and TALE class sizes. (a) Number of classes (black) and maximum p-value of all class assignments (red) plotted against different thresholds on the average distance between TALEs in a common class. Dashed red horizontal line indicates a significance level of α=0.01; dashed blue vertical line indicates the chosen threshold of 5.0. (b) Histogram of the number of classes with one to six class members.

Supplementary Table 1 | *Xoo* PXO83 TALE assignment by AnnoTALE.

TALE name	Type	Start	End	Strand
TalAI3 (putative pseudogene)	initial match	2029504	2032827	+
	coding sequence (ATG to Stop)	2029505	2032519	+
TalCA1	initial match	2322377	2325787	+
	coding sequence (ATG to Stop)	2322377	2325787	+
TalAN3	initial match	2326777	2330631	+
	coding sequence (ATG to Stop)	2326777	2330631	+
TalAF4	initial match	2850017	2853331	+
	coding sequence (ATG to Stop)	2850017	2853331	+
TalAB5	initial match	2856608	2860126	+
	coding sequence (ATG to Stop)	2856608	2860126	+
TalBJ2	initial match	2861116	2864421	+
	coding sequence (ATG to Stop)	2861116	2864421	+
TalAP3	initial match	3796927	3800643	+
	coding sequence (ATG to Stop)	3796927	3800643	+
TalAQ3	initial match	3801633	3806081	+
	coding sequence (ATG to Stop)	3801633	3806081	+
TalAO3	initial match	3807071	3810481	+
	coding sequence (ATG to Stop)	3807071	3810481	+
TalAE4	initial match	3811471	3814479	+
	coding sequence (ATG to Stop)	3811471	3814479	+
TalAD5	initial match	3815469	3819596	+
	coding sequence (ATG to Stop)	3815469	3819596	+
TalAI4 (putative pseudogene)	initial match	2824875	2828207	-
	coding sequence (ATG to Stop)	2825615	2828206	-
TalBA2	initial match	2306051	2309347	-
	coding sequence (ATG to Stop)	2306051	2309347	-
TalAA5	initial match	2301306	2305061	-
	coding sequence (ATG to Stop)	2301306	2305061	-
TalAR3	initial match	2295480	2299499	-
	coding sequence (ATG to Stop)	2295480	2299499	-
TalAG4	initial match	1814083	1817802	-
	coding sequence (ATG to Stop)	1814083	1817802	-
TalAC5	initial match	1808753	1813093	-
	coding sequence (ATG to Stop)	1808753	1813093	-
TalAS3	initial match	555599	560029	-
	coding sequence (ATG to Stop)	555599	560029	-

Supplementary Table 2 | Initial set of TALEs for building TALE classes.

Genomic TALEs				Individual TALEs
***Xoo* PXO99**[A] (CP000967.1)	***Xoo* MAFF311018** (AP008229.1)	***Xoo* KACC10331** (AE013598.1)	***Xoc* BLS256** (CP003057.1)	
tal9c/avrXa27	XOO1132	XOO1237	XOC_0460	***Xoo* PXO86**, avrXa10 (U50552.1)
tal2b/pthXo1	XOO1134	XOO2127	XOC_0463	***Xoo* JXOIII**, avrXa3 (AY129298.1)
tal5b/pthXo6	XOO1136	XOO2128	XOC_0473	***Xoo* JXOIII**, avrXa5 (FJ593881.1)
tal1/pthXo7	XOO1138	XOO2131	XOC_1565	***Xoo* PXO1865**, avrXa7 (AF262933.1)
tal2b	XOO1996	XOO2264	XOC_1566	***Xoo* PXO2684**, avrXa7 (AF275267.1)
tal4	XOO1998	XOO2275	XOC_1567	***Xoo* JX01**, PthXo2 (AY495677.1)
tal5a	XOO2001	XOO2276	XOC_1570	***Xoo* PXO61**, PthXo3 (AY495678.1)
tal6a	XOO2127	XOO2279	XOC_1572	
tal7a	XOO2129	XOO3013	XOC_1583	
tal7b	XOO2158	XOO3014	XOC_1704	
tal8a	XOO2160	XOO3015	XOC_1709	
tal8b	XOO2864	XOO4255	XOC_1716	
tal9a	XOO2865	XOO1238	XOC_2007	
tal9b	XOO2866		XOC_2009	
tal9d	XOO2868		XOC_2010	
tal9e	XOO4014		XOC_2480	
tal3a	XOO2667		XOC_2485	
tal3b			XOC_2857	
tal6b			XOC_2892	
			XOC_4025	
			XOC_4248	
			XOC_4250	
			XOC_4384	
			XOC_4392	
			XOC_4399	
			XOC_4531	

Supplementary Table 3 | RVD codon pairs.

RVD	Codon pairs
NN	AAC AAC, AAT AAC, AAT AAT
ND	AAC GAT, AAT GAC
N*	AAT
NK	AAT AAA
NS	AAT AGC, AAT AGT
NI	AAT ATT, AAT ATA
NH	AAT CAC
NA	AAT GCC
NG	AAT GGC
NC	AAT TGC
SN	AGT AAC
HN	CAC AAT, CAT AAC
HD	CAC GAT, CAT GAT
HA	CAC GCT
HH	CAT CAC
HG	CAT GGC
YG	TAT GGC
S*	AGT
H*	CAT
SS	AGT AGC
HI	CAT ATT
NV	AAT GTT

Supplementary Sequences | Full-length DNA sequences of *Xoo* PXO83 TALEs.

>TalAI3 Xoo PXO83 (Pseudo)
ATGGATCCCATTCGTTCGCGCACGCCAAGTCCTGCCCGCGAGCCTCTGCCCGGACCCCAACCGGATAGGGTTCAGCCGACTGCAGATCGTGGGGT
GTCTGCGCCTGCTGGCAGCCCTCTGGATGGCTTGCCCGCTCGGCGGACGGTGTCCCGGACCCGGCTGCCATCTCCCCCTGCCCCCTTGCCTGCGT
TCTCGGCGGGCAGCTCCACCGATCGGCTCCGTCCGTTCGATCCGTCGCTTCCTGATACATCGCTTTTTGATTCGATGCCTGCCGTCGGCACGCCT
CATACAGAGGCTGCCCCAGCAGACACTTCGCCGGCCGCGCAGGTGGATCTACTCACGCTCGCGACAGTGGCGCAGCACCACGAGGCACTGGTGGG
CCATGGGTTTACACACGCGCACATCGTTGCGCTCAGCCAACACCCGGCAGCGTTAGGGACCGTTGCTGTCACGTATCAAGACATAATCACGGCGT
TGCCAGAGGCGACACACGAAGACATCGTTGGCGTCGGCAAACAGTTGTCCGGCGCACGCGCCCTGGAGGCCTTGCTCACGAAGGCGGGGGAGTTG
AGAGGTCCGCCGTTACAGTTGGACACAGGCCAACTTCTCAAGATTGCAAGACGTGGCGGCGTGACCGCAGTGGAGGCAGTGCATGCATGGCGCAA
TGCACTGACGGGTGCCCCCCTGAACCTGACCCCGGACCAAGTGGTGGCCATCGCCAGCAATAGTGGCGGCAAGCAGGCGCTGGAGACGGTGCAGC
GGCTGTTGCCGGTGCTGTGCCAGGACCATGGCCTGACCCCGGACCAGGTCGTGGCCATCGCCAGCCACGATGGCGGCAAGCAGGCGCTGGAGACG
GTGCAGCGGCTGTTGCCGGTGCTGTGCCAGGACCATGGCCTGACCCCGGACCAGGTCGTGGCCATCGCCAGCAATGGCGGCGGCAAGCAGGCGCT
GGAGACGGTGCAGCGGCTGTTGCCGGTGCTGTGCCAGGACCATGGCCTGACCCCGGACCAGGTGGTGGCCATCGCCAGCAATGGCGGCGGCAAGC
AGGCGCTGGAGACGGTACTGTGCCAGGCCCATGGCCTGACCCCGGCGCAGGTGGTGGCCATCGCCAGCAATGGCGGCGGCAAGCAGGCGCTGGAG
ACGGTGCAGCGGCTGTTGCCGGTGCTGTGCCAGGCCCATGGCCTGACCCTGGACCAGGTCGTGGCCATTGCCAGCAATGGCGGCGGCAAGCAGGC
GCTGGAGACGGTGCAGCGGCTGTTGCCGGTGCTGTGCCAGGCCCATGGCCTGACCCCGGCGCAGGTGGTGGCCATCGCCAGCAATGGCGGCGGCA
AGCAGGCGCTGGAGACGGTGCAGCGGCTGTTGCCGGTGCTGTGCCAGGCCCATGGCCTGACCCCGGCCCAGGTGGTGGCCATCGCCAGCCACGAT
GGCGGCAAGCAGGCGCTGGAGACGGTGCAGCGGCTGTTGCCGGTGCTGTGCCAGGCCCATGGTCTGACCCTGGACCAGGTAGTGGCCATTGCCAG
CCACGATGGCGGCAAGCAGGCGCTGGAGACGGTGCAGCGGCTGTTGCCGGTGCTGTGCCAGGCCCATGGTCTGACCCTGGACCAGGTAGTGGCCA
TTGCCAGCCACGATGGCGGCAAGCAGGCGCTGGAGACGGTGCAGCGGCTGTTGCCGGTGCTGTGCCAGGCCCATGGTCTGACCCCGGCGCAGGTG
GTGGCCATCGCCAGCAATAACGGCGGCAAGCAGGCGCTGGAGACGGTGCAGCGGCTGTTGCCGGTGCTGTGCCAGGACCATGGCCTGACCCCGGA
CCAGGTGGTGGCCATCGCCAGCCACGATGGCGGCAAGCAGGCGCTGGAGACGATGCAGCGGCTGTTGCCGGTGCTGTGCCAGGCCCATGGCCTGA
CCCCGGACCAGGTCGTGGCCATCGCCAGCAATGGCGGCGGCAAGCAGGCGCTGGAGACGGTGCAGCGGCTGTTGCCGGTGCTGTGCCAGGCCCAT
GGCCTGACCCCGGACCAGGTCGTGGCCATCGCCAGCCACGATGGCGGCAAGCAGGCGCTGGAGACGGTGCAGCGGCTGTTGCCGGTGCTGTGCCA
GGACCATGGCCTGACCCCGGACCAGGTCGTGGCCATCGCCAGCAATATTGGCGGCAAGCAGGCGCTGGAGACGGTGCAGCGGCTGTTGCCGGTGC
TGTGCCAGGACCATGGCCTGACCCCGGACCAGGTCGTGGCCATCGCCAGCCACGATGGCGGCAAGCAGGCGCTGGAGACGGTGCAGCGGCTGTTG
CCGGTGCTGTGCCAGGACCATGGCCTGACCCCGGACCAGGTCGTGGCCATCGCCAACAATAACGGCGGCAAGCAGGCGCTGGAGACGGTGCAGCG
GCTGTTGCCGGTGCTGTGCCAGGACCATGGCCTGACCCCGGACCAGGTCGTGGCCATCGCCAGCAATGGCGGCAAGCAGGCGCTGGAGAGCATTG
TTGCCCAGTTATCTCGCCCTGATCCGGCGTTGGCCGCGTTGACCAACGACCACCTCGTCGCCTTGGCCTGCCTCGGCGGACGTCCTGCCCTGGAT
GCAGTGAAAAAGGGATTGCCGCACGCGCCGGAATTGATCAGAAGAGTCAATAGCCGTATTGGCGAACGCACGTCCCATCGCGTTGCCGACCTCGC
GCACGTGGTGCGCGTGCTTGGTTTTTTCCAGAGCCACTCCCACCCAGCGCAAGCATTCGATGACGCCATGACGCAGTTCGGGATGAGCAGGCACG
GGTTGGTACAGCTCTTTCGCAGAGTGGGCGTCACCGAATTCGAAGCCCGCTGCGGAACTATCCCCCCAGCCTCGCAGCGTTGGGACCGTATCCTC
CAGGCATCAGGGACGAAAAGGGCCAAACCGTCCCCTACTTCAGCTCAGACGCCGGATCAGGCGTCTTTGCATGCATTCCCCGACTCGCTGGAGCG
TGACCTTGATGCGCCCAGCCCAATGCACGAGGGAGATCAGACGCGGGCAAGCAGACGTAAACGGTCCTGA
>TalCA1 Xoo PXO83
ATGGATCCCATTCGTTCGCGCACGCCAAGTCCTGCCCGCGAGCTTCTGCCCGGACCCCAACCGGATAGGGTTCAGCCGACTGCAGATCGGGGGGG
GACTCCGCCTGCTGGCGGCCCCCTGGATGGCTTGCCCGTTCGGCGGACGATGTCCCGGACCCGGCTGCCATCTCCCCCTGCACCCTCGCCTGCGT
TCTCGGCGGGCAGCTTCAGCGATCTGCTCCGTCAGTTCGATCCGTCGCTTCTTGATACATCGCTTCTTGATTCGATGCCTGCCGTCGGCACGCCG
CATACAGCGGCTGCCCCAGCAGAGTGGGATGAGGTGCAATCGGGTCTGCGTGCAGCCGATGACCCGCCACCCACTGTGCGTGTCGCTGTCACTGC
CGCGCGGCCGCCGCGCGCCAAGCCGGCCCCGCGACGGCGTGCGGCGCAACCCTCCGACGCTTCGCCGGCCGCGCAGGTGGATCTACGCACGCTCG
GCTACAGTCAGCAGCAGCAAGAGAAGATCAAACCGAAGGTGCGTTCGACAGTGGCGCAGCACCACGAGGCACTGGTGGGCCATGGGTTTACACAC
GCGCACATCGTTGCGCTCAGCCAACACCCGGCAGCGTTAGGGACCGTTGCTGTCACGTATCAGGACATAATCAGGGCGTTGCCAGAGGCGACACA
CGAAGACATCGTTGGCGTCGGCAAACAGTGGTCCGGCGCACGCGCCCTGGAGGCCTTGCTCACGGAGGCGAGGGAGTTGAGAGGTCCGCCGTTAC
AGTTGGACACAGGCCAACTTCTCAAGATTGCAAAACGTGGCGGCGTGACCGCAGTGGAGGCAGTGCATGCATGGCGCAATGCACTGACGGGTGCC
CCCCTGAACCTGACCCCGGACCAAGTGGTGGCCATCGCCAGCAATATTGGCGGCAACCAGGCGCTGGAGACGGTACAGCGGCTGTTGCCGGTGCT
GTGCCAGGCCCATGGCCTGACCCCGGACCAGGTCGTGGCCATCGCCAGCAATGGCGGCAAGCAGGCGCTGGAGACGGTGCAGCGGCTGTTGCCGG
TGCTGTGCCAGGACCATAGCCTGACCCCGGACCAGGTCGTGGCCATCGCCAGCAATATAGGCGGCAAGCAGGCGCTGGAGACGGTGCAGCGGCTG
TTGCCGGTGCTGTGCCAGGACCATGGCCTGACCCCGGACCAGGTGGTGGCCATCGCCAGCAATAGTGGCGGCAAGCAGGCGCTGGAGACGGTGCA
GCGGCTGTTGCCGGTGCTGTGCCAGGACCATGGCCTGACCCCGGACCAGGTGATGACCATCGCCAGCAATAACGGCGGCAAGCAGGCGCTGGAGA
CGGTGCAACGGCTGTTGCCGGTGCTGTGCCAGGACCATGGCCTGACCCCGGACCAGGTGGTGACCATCGCCAGCAATGGCGGCGGCAAGCAGGCG
CTGGAGACGGTGCAGCGGCTGTTGCCGGTGCTGTGCCAGGCCCATGGCCTGACCCCGGACCAGGTGGTGGCCATCGCCAACAATAACGGCGGCAA
GCAGGCGCTGGAGACGGTGCAGCGGCTGTTGCCGGTGCTGTGCCAGGACCATGGCCTGAGTCCGGACCAGGTGGTGGCCATCGCCAGCCACGATG
GCGGCAAGCAGGCGCTGGAGACGGTGCAGCGGCTGTTGCCGGTGCTGTGCCAGGCCCATGGCCTGACCCCGGCCCAAGTGGTGGCCATCGCCAGC
CACGATGGCGGCAAGCAGGCGCTGGAGACGGTGCAGCGGCTGTTGCCGGTGTTGTGCCAGGACCATGGCCTGACCCCGGACCAGGTGGTGGCCAT
CGCCAGCCACGATGGCGGCAAGCAGGCGCTGGAGACGGTGCAGCGGCTGTTGCCGGTGCTGTGCCAGGACCATGGCCTGACCCCGGACCAGGTCG
TGGCCATCGCCAGCAATGGCGGCGGCAAGCAGGCGCTGGAGACGGTGCAGCGGCTGTTGCCGGTGCTGTGCCAGGACCATGGCCTGACCCTGGAC
CAGGTGGTGGCCATCGCCAGCCACGATGGCGGCAAGCAGGCGTTGGAGACGGTGCAGCGGCTGTTGCCGGTGCTGTGCCAGGACCATGGCCTGAC
CCCGAACCAGGTGGTGGCCATCGCCAGCAATAGTGGCGGCAAGCAGGCGCTGGAGACGGTGCAGCGGCTGTTGCCGGTGCTGTGCCAGGACCATG
GCCTGACCCCGGACCAGGTGGTGGCCATCGCCAGCCACGATGGCGGCAAGCAGGCGCTGGAGACGGTGCAGCGGCTGTTGCCGGTGCTGTGCCAG
GACCATGGCCTGACCCCGGTCCAGGTGGTGGCCATCGCCAGCAATGGCGGCAAGCAGGCGCTGGAGACGGTGCAGCGGCTGTTGCCGGTGCTGTG
CCAGGACCATGGCCTGACCCCGGCCCAGGTGGTGGCCATCGCCAGCAATAGTGGCGGCAAGCAGGCGCTGGAGACGGTGCAGCGGCTGTTGCCGG
TGCTGTGCCAGGACCATGGCCTGACCCCGGACCAGGTCGTGGCCATCGCCAGCAATGGCGGCGGCAAGCAGGCGCTGGAGAGCATTGTTGCCCAG
TTATCTCGCCCTGATCCGGCGTTGGCCGCGTTGACCAACGACCACCTCGTCGCCTTGGCCTGCCTCGGCGGACGTCCTGCCCTGGATGCAGTGAA
AAAGGGATTGCCGCACGCGCCGGAATTGATCAGAAGAATCAATCGCCGTATTCCCGAACGCACGTCCCATCGCGTTGCCGACTACGCGCAAGTGG
TTCGCGTGCTGGAGTTTTTCCAGTGCCACTCCCACCCAGCGTACGCATTTGATGAGGCCATGACGCAGTTCGGGATGAGCAGGAACGGGTTGGTA
CAGCTCTTTCGCAGAGTGGGCGTCACCGAACTCGAAGCCCGCGGTGGAACGCTCCCCCCAGCCTCGCAGCGTTGGGACCGTATCCTCCAGGCATC
AGGGATGAAAAGGGCCAAACCGTCCCCTACTTCAGCTCAAACACCGGATCAGGCGTCTTTGCATGCATTCGCCGATTCGCTGGAGCGTGACCTTG
ATGCGCCTAGCCCAATGCACGAGGGAGATCAGACAGGGGCAAGCAGCCGTAAACGGTCCCGATCGGATCGTGCTGTCACCGGCCCCTCCGCACAG
CAATCTTTCGAGGTGCGCGTTCCCGAACAGCACGATGCGCTGCATTTGCCCCTCAGCTGGAGGGTAAAACGCCCGCGTACCAGGATCGGGGGCGG
CCTCCCGGATCCTGGTACGCCCATCGCTGCCGACCTGGCAGCGTCCAGCACCGTGCTGTGGGAACAAGATGCGGCCCCCTTCGCAGGGGCAGCGG
ATGATTTCCCGGCATTCAACGAAGAGGAGCTCGCATGGTTGATGGAGCTATTGCCTCAGTCAGGCTCAGTCGGAGGGACGATC
>TalAN3 Xoo PXO83
ATGGATCCCATTCGTTCGCGCACGCCAAGTCCTGCCCGCGAGCTTCTGCCCGGACCCCAACCGGATAGGGTTCAGCCGACTGCAGATCGGGGGGG
GGCTCCGCCTGCTGGCGGCCCCCTGGATGGCTTGCCCGCTCGGCGGACGATGTCCCGGACCCGGCTGCCATCTCCCCCTGCGCCCTCGCCTGCGT

TCTCGGCGGGCAGCTTCAGCGATCTGCTCCGTCAGTTCGATCCGTCGCTTCTTGATACATCGCTTCTTGATTCGATGCCTGCCGTCGGCACGCCG
CATACAGCGGCTGCCCCAGCAGAGTGGGATGAGGTGCAATCGGGTCTGCGTGCAGCCGATGACCCGCCACCCACCGTGCGTGTCGCTGTCACTGC
CGCGCGGCCGCCGCGCGCCAAGCCGGCCCCGCGACGGCGTGCGGCGCAACCCTCCGACGCTTCGCCGGCCGCGCAGGTGGATCTACGCACGCTCG
GCTACAGTCAGCAGCAGCAAGAGAAGATCAAACCGAAGGTGCGTTCGACAGTGGCGCAGCACCACGAGGCACTGGTGGGCCATGGGTTTACACAC
GCGCACATCGTTGCGCTCAGCCAACACCCGGCAGCGTTAGGGACCGTTGCTGTCACGTATCAGGACATAATCAGGGCGTTGCCAGAGGCGACACA
CGAAGACATCGTTGGCGTCGGCAAACAGTGGTCCGGCGCACGCGCCCTGGAGGCCTTGCTCACGGAGGCGAGGGAGTTGAGAGGTCCGCCGTTAC
AGTTGGACACAGGCCAACTTCTCAAGATTGCAAAACGTGGAGGCGTGACCGCAGTGGAGGCAGTGCATGCATGGCGCAATGCACTGACGGGTGCC
CCCCTGAACCTGACCCCGGACCAAGTGGTGGCCATCGCCAGCAATATTGGCGGCAAGCAGGCGCTGGAGACGGTGCAGCGGCTGTTGCCGGTGCT
GTGCCAGGACCATGGCCTAACCCCGGACCAGGTCGTGGCCATCGCCAGCAATGGCGGCAAGCAGGCGCTGGAGACGGTGCAACGGCTGTTGCCGG
TGCTGTGCCAGGCCCATGGCCTGACCCCGGACCAGGTGGTGGCCATCGCCAGCAATAGTGGCGGCAAGCAGGCGCTGGAGACGGTGCAGCGGCTG
TTGCCGGTGCTGTGCCAGGACCATGGCCTGACCCCGGACCAGGTGGTGGCCATCGCCAGCCATGGCGGCGGCAAGCAGGCGCTGGAGACGGTGCA
GCGGCTGTTGCCGGTGCTGTGCCAGGACCATGGCCTGACCCCGGACCAGGTGGTGGCCATCGCCAGCAATATTGGCGGCAAGCAGGCGCTGGAGA
CGGTGCAGCGGCTGTTGCCGGTGCTGTGCCAGGACCATGGCCTGATCCCGGACCAGGTGGTGGCCATCGCCAGCAATATTGGCGGCAAGCAGGCG
CTGGAGACGGTGCAGCGGCTGTTGCCGGTGCTGTGCCAGGACCATGGCCTGACCCCGGACCAGGTGGTGGCCATCGCCAGCAATAGTGGCGGCAA
GCAGGCGCTGGAGACGGTGCAGCGGCTGTTGCCGGTGCTGTGCCAGGCCCATGGCCTGACCCCGGACCAAGTGGTGGCCATCGCCAGCCACGATG
GCGGCAAGCAGGCGCTGGAGACGGTGCAGCGGCTGTTGCCGGTGCTGTGCCAGGACCATGGCCTGACCCCGGCCCAAGTGGTGGCCATCGCCAGC
AATAACGGCGGCAAGCAGGCGCTGGAGACGGTGCAGCGGCTGTTGCCGGTGCTGTGCCAGGCCCATGGCCTGACCCCAGACCAAGTGGTGGCCAT
CGCCAGCCACGATGGCGGCAAGCAGGCGCTGGAGACGGTGCAGCGGCTGTTGCCGGTGCTGTGCCAGGACCATGGCCTGACCCCGGCCCAAGTGG
TGGCCATCGCCAGCAATAGTGGCGGCAAGCAGGCGCTGGAGACGGTGCAGCGGCTGTTGCCGGTGCTGTGCCAGGACCATGGCCTGACCCCGGAC
CAGGTGGTGGCCATCGCCAGCAATGGCGGCGGCAAGCAGGCGCTGGAGACGGTGCAGCGGCTGTTGCCGGTGCTGTGCCAGGACCATGGCCTGAC
CCCGGACCAGGTCGTGGCCATCGCCAGCAGTAGCGGCGGCAAGCAGGCGCTGGAGACGGTGCAGCGGCTGTTGCCGGTGCTGTGCCAGGCCCATG
GCCTGACCCCGGACCAGGTGGTGGCCATCGCCAGCCACGATGGCGGCAAGCAGGCGCTGGAGACGGTGCAGCGGCTGTTGCCGGTGCTGTGCCAG
GACCATGGCCTGACCCCGGACCAGGTGGTGGCCATCGCCAGCAATATTGGCGGCAAGCAGGCGCTGGAGACGGTGCAGCGGCTGTTGCCGGTGCT
GTGCCAGGACCATGGCCTGACCCCGGACCAGGTGGTGGCCATCGCCAGCAATATTGGCGGCAAGCAGGCGCTGGAGACGGTGCAGCGGCTGTTGC
CGGTGCTGTGCCAGGACCATGGCCTGACCCCGGACCAGGTGGTGGCCATCGCCAACAATAACGGCGGCAAGCAGGCGCTGGAGACGGTGCAGCGG
CTGTTGCCGGTGCTGTGCCAGGACCATGGCCTGACCCCGGACCAGGTGGTGGCCATCGCCAGCAATATTGGCGGCAAGCAGGCGCTGGAGACGGT
GCAGCGGCTGTTGCCGGTGCTGTGCCAGGACCATGGCCTGACCCCGGCCCAGGTGGTGGCCATCGCCAACAATAACGGCGGCAAGCAGGCGCTGG
AGACGGTGCAGCGGCTGTTGCCGGTGCTGTGCCAGGACCATGGCCTGACCCCGGACCAGGTGGTGGCCATCGCCAGCAATATTGGCGGCAAGCAG
GCGCTGGAGACGGTGCAGCGGCTGTTGCCGGTGCTGTGCCAGGACCATGGCCTGACCCCGGACCAGGTGGTGGCCATCGCCAGCAATGGCGGCGG
CAAGCAGGCGCTGGAGAGCATTGTTGCCCAGTTATCTCGCCCTGATCCGGCGTTGGCCGCGTTGACCAACGACCACCTCGTCGCCTTGGCCTGCC
TCGGCGGACGTCCTGCCCTGGATGCAGTGAAAAAGGGATTGCCGCACGCGCCGGAATTGATCAAAAGAATCAATCGCCGCATTCCCGAACGCACG
TCCCATCGCGTTGCCGACCTCCCCGAACGCACGTCCCATCGCGTTGCCGACCTCGCGCACGTGGTGCGCGTGCTTGGTTTTTCCAGAGCCACTC
CCACCCAGCGCAAGCATTCGATGACGCCATGACGCAGTTCGGGATGAGCAGGCACGGGTTGGTACAGCTCTTTCGCAGAGTGGGCGTCACCGAAT
TCGAAGCCCGCTGCGGAACGCTCCCCCCAGCCTCGCAGCGTTGGGACCGTATCCTCCAGGCATCAGGGATGAAAAGGGCCAAACCGTCCCCTACT
TCAGCTCAAACGCCGGATCAGGCGTCTTTGCATGCATTCGCCGATTCGCTGGAGCGTGACCTTGATGCGCCCAGCCCAATGCACGAGGGAGATCA
GACGCGGGCAAGCAGCCGTAAACGGTCCCGATCGGATCGTGCTGTCACCGGCCCCTCCACACAGCAATCTTTCGAGGTGCGCGTTCCCGAACAGC
ACGATGCGCTGCATTTGCCCCTCAGCTGGAGGGTAAAACGCCCGCGTACCAGGATCGGGGGCGGCCTCCCGGATCCTGGTACGCCCATCGCTGCC
GACCTGGCAGCGTCCAGCACCGTGATGTGGGAACAAGATGCGGCCCCCTTCGCAGGGGCAGCGGATGATTTCCCGGCATTCAACGAAGAGGAGCT
CGCATGGTTGATGGAGCTATTGCCTCAGTCAGGCTCAGTCGGAGGGACGATC
>TalAF4 Xoo PXO83
ATGGATCCCATTCGTTCGCGCACGCCAAGTCCTGCCCGCGAGCTTCTGCCCGGACCCCAACCGGATAGGATTCAGCCGACTGCAGATCGGGGGG
GGCTCCGCCTGCTGGCGGCCCCCTGGATGGCTTGCCCGCTCGGCGGACGATGTCCCGGACCCGGCTGCCATCTCCCCCTGCGCCCTCGCCTGCGT
TCTCGGCGGGCAGCTTCAGCGATCTGCTCCGTCAGTTCGATCCGTCGCTTCTTGATACATCGCTTCTTGATTCGATGCCTGCCGTCGGCACGCCG
CATACAGCGGCTGCCCCAGCAGAGTGCGATGAGGTGCAATCGGGTCTGCGTGCAGCCGATGACCCGCCACCCACCGTGCGTGTCGCTGTCACTGC
CGCGCGGCCGCCGCGCGCCAAGCCGGCCCCGCGACGGCGTGCGGCGCAACCCTCCGACGCTTCGCCGGCCGCGCAGGTGGATCTACGCACGCTCG
GCTACAGTCAGCAGCAGCAAGAGAAGATCAAACCGAAGGTGCGTTCGACAGTGGCGCAGCACCACGAGGCACTGGTGGGCCATGGGTTTACACAC
GCGCACATCGTTGCGCTCAGCCAACACCCGGCAGCGTTAGGGACCGTCGCTGTCAAGTATCAGCACATAATCACGGCGTTGCCAGAGGCGACACA
CGAAGACATCGTTGGCGTCGGCAAACAGTGGTCCGGCGCACGCGCCCTGGAGGCCTTGCTCACGAAGGCGGGGGAGTTGAGAGGTCCGCCGTTAC
AGTTGGACACAGGCCAACTTCTCAAGATTGCAAAACGTGGCGGCGTGACCGCAGTGGAGGCAGTGCATGCATGGCGCAATGCACTGACGGGTGCC
CCCCTGAACCTGACCCCGGACCAAGTGGTGGCCATCGCCAGCAATATTGGCGGCAAGCAGGCGCTGGAGACGGTGCAGCGGCTGTTGCCGGTGCT
GTGCCAGGCCCATGGCCTGACCCCGGACCAGGTGGTGGCCATCGCCAACAATAACGGCGGCAAGCAGGCACTGGAGACGGTGCAGCGGCTGTTGC
CGGTGCTGTGCCAGGCCCATGGCCTGACCCCGGCGCAGGTGGTGGCCATCGCCAGCAATAACGGCGGCAAGCAGGCGCTGGAGACGGTGCAGCGG
CTGTTGCCGGTGCTGTGCCAGGACCATGGCCTGACCCCGGACCAAGTGGTGGCCATCGCCAGCAATATTGGCGGCAAGCAGGCGCTGGAGACGAT
GCAACGGCTGTTGCCGGTGCTGTGCCAGGACCATGGCCTGACCCCGGACCAGGTCGTGGCCATCGCCAGCAATATTGGCGGCAAGCAGGCGCTGG
AGACGGTGCAGCGGCTGTTGCCGGTGCTGTGCCAGACCCATGCCCTGACCCCGGACCAGGTGGTGGCCATCGCCAGCAATATTGGCGGCAAGCAG
GCGCTGGAGACGGTGCAGCGGCTGTTGCCGGTGCTGTGCCAGGACCATGGCCTGACCCCGGCGCAGGTGGTGGCCATCGCCAGCCACGATGGCGG
CAAGCAGGCGCTGGAGACGGTGCAGCGGCTGTTGCCGGTGCTGTGCCAGGACCATGGCCTGACCCCGGACCAGGTGGTGGCCATCGCCAGCAATA
GTGGCGGCAAGCAGGCGCTGGAGACGGTGCAGCGGCTGTTGCCGGTGCTGTGCCAGGCCCATGGCCTGACCCTGGACCAGGTGGTGGCCATCGCC
AGCCATGGCGGCGGCAAGCAGGCGCTGGAGACGGTGCAGCGGCTGTTGCCGGTGCTGTGCCAGGACCATGGCCTGACCCCGGACCAGGTGGTGGC
CATCGCCAGCAATAACGGCGGCAAGCAGGCGCTGGAGACGGTGCAGCGGCTGTTGCCGGTGCTGTGCCAGGACCATGGCCTGATCCCGGACCAGG
TGGTGGCCATCGCCAACAATAACGGCGGCAAGCAGGCGCTGGAGACGGTGCAGCGGCTGTTGCCGGTGCTGTGCCAGGCCCATGGCCTGACCACG
GACCAGGTGGTGACCATCGCCAGCAATAACGGCGGCAAGCAGGCGCTGGAGACGGTGCAACGGCTGTTGCCGGTGCTGTGCCAGGACCATGGCCT
GACCCCGGACCAGGTCGTGGCCATCGCCAGCAATATTGGCGGCAAGCAGGCGCTGGAGACGGTGCAGCGGCTGTTGCCGGTGCTGTGCCAGGACC
ATGGCCTGACCCCGGACCAGGTGGTGGCCATCGCCAGCAATATTGGCGGCAAGCAGGCGCTGGAGACGGTGCAGCGGCTGTTGCCGGTGCTGTGC
CAGGACCATGGCCTGACCCCGGACCAGGTCGTGGCCATCGCCAGCAATGGCGGCGGCAAGCAGGCGCTGGAGACGGTGCAGCGGCTGTTGCCGGT
GCTGTGCCAGGACCATGGCCTGACCCCGGACCAGGTGGTGGCCATCGCCAGCCACGATGGTGGCAAGCAGGCGCTGGAGAGCATTGTTGCCCAGT
TATCTCGCCCTGATCCGGCGTTGGCCGCGTTGACCAACGACCACCTCGTCGCCTTGGCCTGCCTCGGCGGACGTCCTGCCCTGGATGCAGTGAAA
AAGGGATTGCCGCACGCGCCGGAATTGATCAGAAGAATCAATCGCCGTATTCCCGAACGCACGTCCCATCGCGTTGCCGACTACGCGCAAGTGGT
TCGCGTGCTGGAGTTTTTCCAGTGCCACTCCCACCCAGCGTACGCATTTGATGAGGCCATGACGCAGTTCGGGATGAGCAGGAACGGGTTGGTAC
AGCTCTTTCGCAGAGTGGGCGTCACCGAACTCGAAGCCCGCGGTGGAACGCTCCCCCCAGCCTCGCAGCGTTGGGACCGTATCCTCCAGGCATCA
GGGATGAAAAGGGCCAAACCGTCCCCTACTTCAGCTCAAACACCGGATCAGGCGTCTTTGCATGCATTCGCCGATTCGCTGGAGCGTGACCTTGA
TGCGCCTAGCCCAATGCACGAGGGAGATCAGACAGGGGCAAGCAGCCGTAAACGGTCCCGATCGGATCGTGCTGTCACCGGCCCCTCCGCACAGC
AATCTTTCGAGGTGCGCGTTCCCGAACAGCACGATGCGCTGCATTTGCCCCTCAGCTGGAGGGTAAAACGCCCGCGTACCAGGATCGGGGGCGGC
CTCCCGGATCCTGGTACGCCCATCGCTGCCGACCTGGCAGCGTCCAGCACCGTGATGTGGGAACAAGATGCGGCCCCCTTCGCAGGGGCAGCGGA
TGATTTCCCGGCATTCAACGAAGAGGAACTCGCATGGTTGATGGAGCTATTGCCTCAGTCAGGCTCAGTCGGAGGGACGATC
>TalAB5 Xoo PXO83

ATGGATCCCATTCGTTCGCGCACGCCAAGTCCTGCCCGCGAGCTTCTGCCCGGACCCCAACCGGATAGGGTTCAGCCGACTGCAGATCGGGGGGG
GGCTCCGCCTGCTGGCGGCCCCCTGGATGGCTTGCCCGCTCGGCGGACGATGTCCCGGACCCGGCTGCCATCTCCCCCTGCGCCCTCGCCTGCGT
TCTCGGCGGGCAGCTTCAGCGATCTGCTCCGTCAGTTCGATCCGTCGCTTCTTGATACATCGCTTCTTGATTCGATGCCTGCCGTCGGCACGCCG
CATACAGCGGCTGCCCCAGCAGAGTGCGATGAGGTGCAATCGGGTCTGCGTGCAGCCGATGACCCGCCACCCACCGTGCGTGTCGCTGTCACTGC
CGCGCGGCCGCCGCGCGCCAAGCCGGCCCCGCGACGGCGTGCGGCGCAACCCTCCGACGCTTCGCCGGCCGCGCAGGTGGATCTACGCACGCTCG
GCTACAGTCAGCAGCAGCAAGAGAAGATCAAACCGAAGGTGCGTTCGACAGTGGCGCAGCACCACGAGGCACTGGTGGGCCATGGGTTTACACAC
GCGCACATCGTTGCGCTCAGCCAACACCCGGCAGCGTTAGGGACCGTTGCTGTCACGTATCAGGACATAATCAGGGCGTTGCCAGAGGCGACACA
CGAAGACATCGTTGGCGTCGGCAAACAGTGGTCCGGCGCACGCGCCCTGGAGGCCTTGCTCACGGAGGCGGGGGAGTTGAGAGGTCCGCCGTTAC
AGTTGGACACAGGCCAACTTCTCAAGATTGCAAAACGTGGCGGCGTGACCGCAGTGGAGGCAGTGCATGCATGGCGCAATGCACTGACGGGTGCC
CCCCTGAACCTGACCCCGGACCAAGTGGTGGCCATCGCCAGCAATATTGGCGGCAACCAGGCGCTGGAGACGGTGCAGCGGCTGTTGCCGGTGCT
GTGCCAGGCCCATGGCCTGACCCCGGACCAGGTCGTGGCCATCGCCAGCCATGGCGGCGGCAAGCAGGCGCTGGAGACGGTGCAGCGGCTGTTGC
CGGTGCTGTGCCAGGACCATGGCCTGACCCCGGACCAGGTGGTGGCCATCGCCAGCAATATTGGCGGCAAGCAGGCGCTGGAGACGGTGCAACGG
CTGTTGCCGGTGCTGTGCCAGGACCATGGCCTGACCCCGGACCAGGTGGTGGCCATCGCCAGCAATATTGGCGGCAAGCAGGCGCTGGAGACGGT
GCAGCGGCTGTTGCCGGTGCTGTGCCAGGACCATGGCCTGACCCCGGACCAGGTGGTGGCCATCGCCAGCAATATTGGCGGCAAGCAGGCGCTGG
AGACGGTGCAGCGGCTGTTGCCGGTGCTGTGCCAGGACCATGGCCTGACCCCGGACCAGGTGGTGGCCATCGCCAACAATAACGGCGGCAAGCAG
GCGCTGGAGACGGTGCAACGGCTGTTGCCGGTGCTGTGCCAGGACCATGGCCTGACCCCGGACCAGGTGGTGGCCATCGCCAGCCACGATGGCGG
CAAGCAGGCGCTGGAGACGGTGCAGCGGCTGTTGCCGGTGCTGTGCCAGGGCCATGGCCTGACCCCGGACCAGGTGGTGGCCATCGCCAGCAATA
GTGGCGGCAAGCAGGCGCTGGAGACGGTGCAGCGGCTGTTGCCAGTGCTGTGCCAGGCCCATGGCCTGACCCCGGACAAGGTGGTGGCCATCGCC
AACAATAACGGCGGCAAGCAGGCGCTGGAGACGGTGCAGCGGCTGTTGCCGGTGCTGTGCCAGGACCATGGCCTGACCCCGGACCAGGTGGTGGC
CATCGCCAGCAATAGTGGCGGCAAGCAGGCGCTGGAGACGGTGCAGCGGCTGTTGCCGGTGCTGTGCCAGGACCATGGTCTGACCCCGGCCCAGG
TGGTGGCCATCGCCAACAATAACGGCGGCAAGCAGGCGCTGGAGACGGTGCGGCGGCTGTTGCCGGTGCTGTGCCAGGACCATGGCCTGACCCCG
GACCAGGTCGTGGCCATCGCCAGCCACGATGGCGGCAAGCAGGCGCTGGAGACGGTGCAGCGGCTGTTGCCGGTACTGTGCCAGGACCATGGCCT
GACCCCGGACCAGGTGGTGGCCATCGCCAACAATAACGGCGGCAAGCAGGCGCTGGAGACGGTGCAGCGGCTGTTGCCGGTGCTGTGCCAGAACC
ATGGCCTGACCCCGGACCAGGTCGTGGCCATCGCCAGCAATATTGGCGGCAAGCAGGCGCTGGAGACGGTGCAGCGGCTGTTGCCGGTGCTGTGC
CAGGACCATGGCCTGACCCCGGACCAGGTGGTGGCCATCGCCAGCCACGATGGCGGCAAGCAGGCGCTGGAGACGGTGCAGCGGCTGTTGCCGGT
GCTGTGCCAGGACCATGGCCTGACCCCGGCCAAGGTGGTGGCCATCGCCAACAATAACGGCGGCAAGCAGGCGCTGGAGACGGTGCGGCGGCTGT
TGCCGGTGCTGTGCCAGGACCATGGCCTGAGCCCGGACCAGGTCGTGGCCATCGCCAGCAATAGTGGCGGCAAGCAGGCGCTGGAGACGGTGCAG
CGGCTGTTGCCGGTGCTGTGCCAGGACCATGGCCTGACCCCGGCCCAGGTGGTGGCCATCGCCAGCAATGGCGGCGGCAAGCAGGCGCTGGAGAG
CATTGTTGCCCAGTTATCTCGCCCTGATCCGGCGTTGGCCGCGTTGACCAACGACCACCTCGTCGCCTTGGCCTGCCTCGGCGGACGTCCTGCCC
TGGATGCAGTGAAAAAGGGATTGCCGCACGCGCCGGAATTGATCAGAAGAATCAATCGCCGTATTCCCGAACGCACGTCCCATCGCGTTGCCGAC
TACGCGCAAGTGGTTCGCGTGCTGGAGTTTTTCCAGTGCCACTCCCACCCAGCGTACGCATTTGATGAGGCCATGACGCAGTTCGGGATGAGCAG
GAACGGGTTGTTACAGCTCTTTCGCAGAGTGGGCGTCACCGAACTCGAAGCCCGCGGTGGAACGCTCCCCCCAGCCTCGCAGCGTTGGGACCGTA
TCCTCCAGGCATCAGGGATGAAAAGGGCCAAACCGTCCCCTACTTCAGCTCAAACACCGGATCAGGCGTCTTTGCATGCATTCGCCGATTCGCTG
GAGCGTGACCTTGATGCGCCTAGCCCAATGCACGAGGGAGATCAGACAGGGGCAAGCAGCCGTAAACGGTCCCGATCGGATCGTGCTGTCACCGG
CCCCTCCGCACAGCAATCTTTCGAGGTGCGCGTTCCCGAACAGCACGATGCGCTGCATTTGCCCCTCAGCTGGAGGGTAAAACGCCCGCGTACCA
GGATCGGGGGCGGCCTCCATGATCCTGGTACGCCCATCGCTGCCGACCTGGCAGCGTCCAGCACCGTGATGTGGGAACAAGATGCGGCCCCCTTC
GCAGGGGCAGCGGATGATTTCCCGGCATTCAACGAAGAGGAGCTCGCATGGTTGATGGAGCTATTGCCTCAGTCAGGCTCAGTCGGAGGGACGAT
C
>TalBJ2 Xoo PXO83
ATGGATCCCATTCGTTCGCGCACGCCAAGTCCTGCCCGCGAGCTTCTGCCCGGACCCCAACCGGATAGGGTTCAGCCGACTGCAGATCGGGGGGG
GGCTCCGCCTGCTGGCGGCCCCCTGGATGGCTTGCCCGCTCGGCGGACGATGTCCCGGACCCGGCTGCCATCTCCCCCTGCGCCCTCGCCTGCGT
TCTCGGCGGGCAGCTTCAGCGATCTGCTCCGTCAGTTCGATCCGTCGCTTCTTGATACATCGCTTCTTGATTCGATGCCTGCCGTCGGCACGCCG
CATACAGCGGCTGCCCCAGCAGAGTGCGATGAGGTGCAATCGGGTCTGCGTGCAGCCGATGACCCGCCACCCACCGTGCGTGTCGCTGTCACTGC
CGCGCGGCCGCCGCGCGCCAAGCCGGCCCCGCGACGGCGTGCGGCGCAACCCTCCGACGCTTCGCCGGCCGCGCAGGTGGATCTACGCACGCTCG
GCTACAGTCAGCAGCAGCAAGAGAAGATCAAACCGAAGGTGCGTTCGACAGTGGCGCAGCACCACGAGGCACTGGTGGGCCATGGGTTTACACAC
GCGCACATCGTTGCGCTCAGCCAACACCCGGCAGCGTTAGGGACCGTTGCTGTCACGTATCAGGACATAATCAGGGCGTTGCCAGAGGCGACACA
CGAAGACATCGTTGGCGTCGGCAAACAGTGGTCCGGCGCACGCGCCCTGGAGGCCTTGCTCACGGAGGCGGGGGAGTTGAGAGGTCCGCCGTTAC
AGTTGGACACAGGCCAACTTCTCAAGATTGCAAAACGTGGCGGCGTGACCGCAGTGGAGGCAGTGCATGCATGGCGCAATGCACTGACGGGTGCC
CCCCTGAACCTGACCCCGGACCAAGTGGTGGCCATCGCCAGCAATATTGGCGGCAACCAGGCGCTGGAGACGGTGCAGCGGCTGTTGCCGGTGCT
GTGCCAGGCCCATGGCCTGACCCCGGACCAGGTCGTGGCCATCGCCAGCCATGGCGGCAAGCAGGCGCTGGAGACGGTGCAGCGGCTGTTGCCGG
TGCTGTGCCAGGCCCATGGCCTGACCCCGGACCATGTGGTGGCCATCGCCAGCAATATTGGCGGCAAGCAGGCGCTGGCGACGGTGCAGCGGCTG
TTGCCGGTGCTGTGCCAGGACCATGGCCTGACCCCGGACCAGGTCGTGGCCATCGCCAGCCATGGCGGCGGCAAGCAGGCGCTGGAGACGGTGCA
GCGGCTGTTGCCGGTGCTGTGCCAGGACCATGGCCTGACCCCGGACCAGGTGGTGGCCATCGCCAGCAATATTGGCGGCAAGCAGGCGCTGGAGA
CGGTGCAGCGGCTGTTGCCGGTGCTGTGCCAGGACCATGGCCTGACCCCGGACCAGGTCGTGGCCATCGCCAGCAATATTGGCGGCAAGCAGGCG
CTGGAGACGGTGCAGCGGCTGTTGCCGGTGCTGTGCCAGGACCATGGCCTGACCCCGGACCAGGTGGTGGCCATCGCCAACAATAACGGCGGCAA
GCAGGCGCTGGAGACGGTGCAACGGCTGTTGCCGGTGCTGTGCCAGACCCATGGCCTGACCCCGGACCAGGTCGTGGCCATCGCCAGCCACGATG
GCGGCAAGCAGGCGCTGGAGACGGTGCAGCGGCTGTTGCCGGTGCTGTGCCAGGACCATGGCCTGACCCCGGACCAGGTCGTGGCCATCGCCAGC
AATATTGGCGGCAAGCAGGCGCTGGCGACGGTGCAGCGGCTGTTGCCGGTGCTGTGCCAGGCCCATGGCCTGACCCCGGACCAGGTCGTGGCCAT
CGCCAGCCACGATGGCGGCAAGCAGGCGCTGGAGACGGTGCAGCGGCTGTTGCCGGTGCTGTGCCAGGACCATGGACTGACCCCGGACCAGGTGG
TGGCCATCGCCAACAATAACGGCGGCAAGCAGGCGCTGGAGACGGTGCAGCGGCTGTTGCCGGTGCTGTGCCAGGACCATGGCCTGACCCCGGCC
CAGGTGGTGGCCATCGCCAGCCATGGCGGCGGCAAGCAGGCGCTGGAGACGGTGCAGCGGCTGTTGCCGGTGCTGTGCCAGGACCATGGCCTGAC
CCCGGTCCAGGTGGTGGCCATCGCCAGCAATAGCGGCGGCAAGCAGGCGCTGGAGACGGTGCAGCGGCTGTTGCCGGTGCTGTGCCAGGACCATG
GCCTGACCCCGGTCCAGGTGGTGGCCATCGCCAGCAATGGCGGCAAGCAGGCGCTGGCGACGGTGCAGCGGCTGTTGCCGGTGCTGTGCCAGGCC
CATGGCCTGACCCCGGTCCAGGTGGTGGCCATCGCCAGCCACGATGGCGGCAAGCAGGCGCTGGAGACGGTGCAACGGCTGTTGCCGGTGCTGTG
CCAGGACCATGGCCTGACCCCAGACCAGGTGGTGGCCATCGCCAGCAATGGCGGCAAGCAGGCGCTGGAGAGCATTGTTGCCCAGTTATCTCGCC
CTGATCCGGCGTTGGCCGCGTTGACCAACGACCACCTCGTCGCCTTGGCCTGCCTCGGCGGACGTCCTGCCCTGGATGCAGTGAAAAAGGGATTG
CCGCACGCACCGGAATTGATCAGAAGAATCAATCGCCGTATTCCCGAACGCACGTCCCATCGCGTTGCCGACCTCGCGCACGTGGTGCGCGTGCT
TGGTTTTTTCCAGAGCCACTCCCACCCAGCGCAAGCATTCGATGACGCCATGACGCAGTTCGGGATGAGCAGGCACGGGTTGGCACAGCTCTTTC
GCAGAGTGGGCGTCACCGAACTCGAAGCCCGCTACGGAACGCTCCCCCCAGCCTCGCAGCGTTGGGACCGTATCCTCCAGGCATCAGGGATGAAA
AGGGCCAAACCGTCCCCTACTTCAGCTCAAACGCCGGATCAGGCGTCTTTGCATGCATTCGCCGATTCGCTGGAGCGTGACCTTGATGCGCCCAG
CCCAATGCACGAGGGAGATCAGACGCGGGCAAGCAGCCGTAAACGGTCCCGATCGGATCGTGCTGTCACCGGCCCCTCCACACAGCAATCTTTCG
AGGTGCGCGTTCCCGAACAGCAAGATGCGCTGCATTTGCCCCTCAGCTGGAGGGTAAAACGCCCGCGTACCAGGATCGGGGGCGGCCTCCCGGAT
CCTGGTACGCCCATCGCTGCCGACCTGGCAGCGTCCAGCACCGTGATGTGGGAACAAGATGCGGCCCCCTTCGCAGGGGCAGCGGATGATTTCCC
GGCATTCAACGAAGAGGAGCTCGCATGGTTGATGGAGCTATTGCCTCAGTCAGGCTCAGTCGGAGGGACGATC
>TalAP3 Xoo PXO83
ATGGATCCCATTCGTTCGCACACGCCAAGTCCTGCCCGCGAGCTTCTGCCCGGACCCCAACCGGATAGGGTTCAGCCGACTGCAGATCGGGGGGG
GGCTCCGCCTGCTGGCGGCCCCCTGGATGGCTTGCCCGCTCGGCGGACGATGTCCCGGACCCGGCTGCCATCTCCCCCTGCGCCCTCGCCTGCGT

TCTCGGCGGGCAGCTTCAGCGATCTGCTCCGTCAGTTCGATCCGTCGCTTCTTGATACATCGCTTCTTGATTCGATGCCTGCCGTCGGCACGCCG
CATACAGCGGCTGCCCCAGCAGAGTGGGATGAGGTGCAATCGGGTCTGCGTGCAGCCGATGACCCGCCACCCACCGTGCGTGTCGCTGTCACTGC
CGCGCGGCCGCCGCGCGCCAAGCCGGCCCCGCGACGGCGTGCGGCGCAACCCTCCGACGCTTCGCCGGCCGCGCAGGTGGATCTACGCACGCTCG
GCTACAGTCAGCAGCAGCAAGAGAAGATCAAACCGAAGGTGCGTTCGACAGTGGCGCAGCACCACGAGGCACTGGTGGGCCATGGGTTTACACAC
GCGCACATCGTTGCGCTCAGCCAACACCCGGCAGCGTTAGGGACCGTTGCTGTCACGTATCAGGACATAATCAGGGCGTTGCCAGAGGCGACACA
CGAAGACATCGTTGGCGTCGGCAAACAGTGGTCCGGCGCACGCGCCCTGGAGGCCTTGCTCACGGAGGCGGGGGAGTTGAGAGGTCCGCCGTTAC
AGTTGGACACAGGCCAACTTCTCAAGATTGCAAAACGTGGCGGCGTGACCGCAGTGAAGGCAGTGCATGCATGGCGCAATGCACTGACGGGTGCC
CCCCTGAACCTGACCCCGGCACAGGTGGTGGCCATCGCCAGCCACGATGGCGGCAATCAGGCGCTGGAGACGGTGCAGCGGCTGTTGCCGGTGCT
GTGCCAGGACCATGGCCTGACCCCGGCGCAGGTGGTGGCCATCGCCAGCCACGATGGCGGCAAGCAGGCTCTGGAGACGGTGCAGCGGCTGTTGC
CGGTGCTGTGCCAGGACCATGGCCTGACCCCGGACCAGGTCGTGGCCATCGCCAGCCACGATGGCGGCAAGCAGGCGCTGGAGACGGTGCAGCGG
CTGTTGCCGGTGCTGTGCCAGGACCATGGCCTGACCCCGGCGCAGGCGGTGGCCATCGCCAGCAATGGCGGCGGCAAGCAGGCGCTGGAGACGGT
GCAACGGCTGTTGCCGGTGCTGTGCCAGGACCATGGCCTGACCCCGGACCAGGTGGTGGCCATCGCCAGCAATGGCGGCAAGCAGGCGCTGGAGA
CGGTGCAGCGGCTGTTGCCGGTGCTGTGCCAGGACCATGGCCTGACCCCGGACCAGGTCGTGGCCATTGCCAGCAATAACGGCGGCAAGCAGGCG
CTGGAGACGGTGCAGCGGCTGTTGCCGGTGCTGTGCCAGGACCATGGCCTGACCCCGGACCAGGTCGTGGCCATCGCCAGCCACGATGGCGGCAA
GCAGGCGCTGGAGACGGTGCAGCGGCTGTTGCCGGTGCTGTGCCAGGACCATGGCCTGACCCCGGACCAGGTCGTGGCCATCGCCAGCCACGATG
GCGGCAAGCAGGCGCTGGAGACGGTGCAGCGGCTGTTGCCGGTGCTGTGCCAGGACCATGGCCTGACCCCGGACCAGGTGGTGGCCATCGCCAGC
AATGGCGGCAAGCAGGCGCTGGAGACGGTGCAGCGGCTGTTGCCGGTGCTGTGCCAGGACCATGGCCTGACCCTGGCGCAGGTGGTGGCCATCGC
CAGCAATATTGGCGGCAAGCAGGCGCTGGAGACGGTGCAGCGGCTGTTGCCGGTGCTGTGCCAGGACCATGGCCTGACCCCGGACCAGGTCGTGG
CCATCGCCAGTAATATTGGCGGCAAGCAGGCGCTGGAGACGGTGCAGCGGCTGTTGCCGGTGCTGTGCCAGGACCATGGCCTGACCCCGGACCAG
GTCGTGGCCATCGCCAGCAATAACGGCGGCAAGCAGGCGCTGGAGACGGTGCAGCGGCTGTTGCCGGTGCTGTGCCAGGACCATGGCCTGACCCC
GGACCAGGTCGTGGCCATCGCCAGCCACGATGGCGGCAAGCAGGCGCTGGAGACGGTGCAGCGGCTGTTGCCGGTGCTGTGCCAGGACCATGGCC
TGACCCCGGCGCAGGTGGTGGCCATCGCCAGCCATATTGGCGGCAAGCAGGCGCTGGAGACGGTGCAGCGGCTGTTGCCGGTGCTGTGCCAGGAC
CATGGCCTGACCCTGGACCAGGTGGTGGCCATTGCCAGCAATGACGGCAGCAAGCAGGCGCTGGAGACGGTGCAGCGGCTGTTGCCGGTGCTGTG
CCAGGACCATGGCCTGACCCCGGACCAGGTGGTGGCCATCGCCAGCCACGATGGCGGCAAGCAGGCGCTGGAGACGGTGCAGCGGCTGTTGCCGG
TGCTGTGCCAGGACCATGGCCTGACCCCGAACCAAGTGGTGGCCATCGCCAGCAATATTGGCGGCAAGCAGGCGCTGGAGACGGTGCAGCGGCTG
TTGCCGGTGCTGTGCCAGGCCCATGGCCTGACCCCGGACCAGGTCGTGGCCATCGCCAGCCACGATGGCGGCAAGCAGGCGCTGGAGACGGTGCA
GCGGCTGTTGCCGGTGCTGTGCCAGAACCATGGCCTGACCCCGGACCAGGTCGTGGCCATCGCCAGCAATGGCGGCGGCAAGCAGGCGCTGGAGA
CGGTGCAACGGCTGTTGCCGGTGCTGTGCCAGGACCATGGCCTGACCCCGGACCAGGTCGTGGCCATCGCCAGCAATGGCGGCGGCAAGCAGGCG
CTGGAGAGCATTGTTGCCCAGTTATCTCGCCCTGATCCGGCGTTGGCCGCGTTGACCAACGACCACCTCGTCGCCTTGGCCTGCCTCGGCGGACG
TCCTGCCCTGGATGCAGTGAAAAAGGGATTGCCGCACGCGCCGGAATTGATCAGAAGAATCAATCGCCGTATTCCCGAACGCACGTCCCATCGCG
TTGCCGACCTCGCGCACGTGGTGCGCGTGCTTGGTTTTTCCAGAGCCACTCCCACCCAGCGCAAGCATTCGATGACGCCATGACGCAGTTCGGG
ATGAGCAGGCACGGGTTGGTACAGCTCTTTCGCAGAGTGGGCGTCACCGAATTCGAAGCCCGCTGCGGAACGCTCCCCCCAGCCTCGCAGCGTTG
GGACCGTATCCTCCAGGCATCAGGGATGAAAAGGGCCAAACCGTCCCCTACTTCAGCTCAAACACCGGATCAGGCGTCTTTGCATGCATTCGCCG
ATTCGCTGGAGCGTGACCTTGATGCGCCCAGCCCAATGCACGAGGGAGATCAGACGCGGGCAAGCAACCGTAAACGGTCCCGATCGGATCGTGCT
GTCACCGGCCCCTCCACACAGCAATCTTTCGAGGTGCGCGTTCCCGAACAGCGCGATGCGCTGCATTTGCCCCTCAGCTGGAGGGTAAAACGCCC
GCGTACCAGGATCGGGGGCGGCCTCCCGGATCCTGGTACGCCCATCGCTGCCGACCTGGCAGCGTCCAGCACCGTGATGTGGGAACAAGATGCGG
CCCCCTTCGCAGGGGCAGCGGATGATTTCCCGGCATTCAACGAAGAGGAGCTCGCATGGTTGATGGAGCTATTGCCTCAGTCAGGCTCAGTCGGA
GGGACGATC
>TalAQ3 Xoo PXO83
ATGGATCCCATTCGTTCGCGCACGCCAAGTCCTGCACGCGAGCTTCTGCCCGGACCCCAACCGGATAGGGTTCAGCCGACTGCAGATCGGGGGGG
GGCTCCGCCTGCTGGCGGCCCCCTGGATGGCTTGCCCGCTCGGCGGACGATGTCCCGGACCCGGCTGCCATCTCCCCCTGCGCCCTCGCCTGCGT
TCTCGGCGGGCAGCTTCAGCGATCTGCTCCGTCAGTTCGATCCGTCGCTTCTTGATACATCGCTTCTTGATTCGATGCCTGCCGTCGGCACGCCG
CATACAGCGGCTGCCCCAGCAGAGTGGGATGAGGTGCAATCGGGTCTGCGTGCAGCCGATGACCCGCCACCCACCGTGCGTGTCGCTGTCACTGC
CGCGCGGCCGCCGCGCGCCAAGCCGGCCCCGCGACGGCGTGCGGCGCAACCCTCCGACGCTTCGCCGGCCGCGCAGGTGGATCTACGCACGCTCG
GCTACAGTCAGCAGCAGCAAGAGAAGATCAAACCGAAGGTGCGTTCGACAGTGGCGCAGCACCACGAGGCACTGGTGGGCCATGGGTTTACACAC
GCGCACATCGTTGCGCTCAGCCAACACCCGGCAGCGTTAGGGACCGTCGCTGTCACGTATCAGCACATAATCACGGCGTTGCCAGAGGCGACACA
CGAAGACATCGTTGGCGTCGGCAAACAGTGGTCCGGCGCACGCGCCCTGGAGGCCTTGTTCACGAAGGCGGGGGAGTTGAGAGGTCCGCCGTTAC
AGTTGGACACAGGCCAACTTCTCAAGATTGCAAAACGTGGCGGCGTGACCGCAGTGGAGGCAGTGCATGCATCGCGCAATGCACTGACGGGTGCC
CCCCTGAACCTGACCCCGGACCAAGTGGTGGCCATCGCCAGCCACGATGGCGGCAAGCAGGCACTGGAGACGGTGCAGCGGCTGTTGCCGGTGCT
GTGCCAGGCCCATGGCCTGACCCCGGCGCAGGTGGTGGCCATCGCCAGCCACGATGGCGGCAAGCAGGCGCTGGAGACGGTGCAGCGGCTGTTGC
CGGTGCTGTGCCAGGCCCATGGTCTGACCCCGGCGCAGGTGGTGGCCATCGCCAGCAATAACGGCGGCAAGCAGGCGCTGGAGACGGTGCAGCGG
CTGTTGCCGGTGCTGTGCCAGGCCCATGGCCTGACCCCGGACCAGGTGGTGGCCATCGCCAGCAATAACGGCGGCAAGCAGGCGCTGGAGACGGT
GCAGCGGCTGTTGCCGGTGCTGTGCCAGGCCCATGGCCTGACCCCAGACCAGGTGGTGGCCATCGCCAGCAATAGTGGCGGCAAGCAGGCGCTGG
AGACGGTGCAGCGGCTGTTGCCGGTGCTGTGCCAGGCCCATGGCCTGACCCCGGACCAGGTCGTGGCCATCGCCAGCAATGGCGGCGGCAAGCAG
GCGCTGGAGACGGTGCAGCGGCTGTTGCCGGTGCTGTGCCAGGCCCATGGCCTGACCCCGGACCAGGTGGTGGCCATCGCCAGCCACGATGGCGG
CAAGCAGGCGCTGGAGACGGTGCAGCGGCTGTTGCCGGTGCTGTGCCAGGCCCATGGCCTGACCCCGGCGCAGGTGGTGGCCATCGCCAGCAGTG
GCGGCAAGCAGGCGCTGGAGACGGTGCAGCGGCTGTTGCCGGTGCTGTGCCAGGACCATGGCCTGACCCCGGACCAGGTGGTGGCCATCGCCAGC
CATGGCGGCAGCAAGCAGGCGCTGGAGACGGTGCAGCGGCTGTTGCCGGTGCTGTGCCAGGCCCATGGCCTGACCCCGGACCAGGTGGTGGCCAT
CGCCAGCCACGATGGCGGCAAGCAGGCGCTGGAGACGGTGCAGCGGCTGTTGCCGGTGCTGTGCCAGGCCCATGGCCTGACCCCGGCGCAGGCGG
TGGCCATCGCCAGCAATGGCGGCGGCAAGCAGGCGCTGGAGACGGTGCAGCGCCTGTTGCCGGTGCTGTGCCAGGACCATGGCCTGACCCCGGAC
CAGGTCGTGGCCATCGCCAGCAATGGCGGCAAGCAGGCGCTGGAGACGGTGCAGCGCCTGTTGCCGGTGCTGTGCCAGGACCATGGCCTGACCCC
GGACCAGGTCGTGGCCATCGCCAGCCACGATGGCGGCAAGCAGGCGCTGGCGACGGTGCAGCGGCTGTTGCCGGTGCTGTGCCAGGCCCATGGCC
TGACCCCGGACCAGGTCGTGGCCATCGCCAGCCACGATGGCGGCAAGCAGGCGCTGGAGACGGTGCAGCGGCTGTTGCCGGTGCTGTGCCAGGAC
CATGGCCTGACCCCGGACCAGGTGGTGGCCATCGCCAGCCACGATGGCGGCAAGCAGGCGCTGGAGACGGTGCAGCGGCTGTTGCCGGTGCTGTG
CCAGGACCATGGCCTGACCCCGGACCAGGTGGTGGCCATCGCCAGCAATGGCGGCAAGCAGGCGCTGGAGACGGTGCAGCGGCTGTTGCCGGTGC
TGTGCCAGGACCATGGCCTGACCCCGGACCAGGTGGTGGCCATCGCCAACAATAACGGCGGCAAGCAGGCGCTGGAGACGGTGCAGCGGCTGTTG
CCGGTGCTGTGCCAGGACCATGGCCTGACCCCGGACCAGGTCGTGGCCATCGCCAGTAATCAGGTGGTGGCCATCGCCAGTAATATTGGCGGCAA
GCAGGCGCTGGAGACGGTGCAGCGGCTGTTGCCGGTGCTGTGCCAGGACCATGGCCTGACCCCGGACCAGGTCGTGGCCATCGCCAGCAATAACG
GCGGCAAGCAGGCGCTGGAGACGGTGCAGCGGCTGTTGCCGGTGCTGTGCCAGGACCATGGCCTGACCCCGGACCAGGTCGTGGCCATCGCCAGC
CACGATGGCGGCAAGCAGGCGCTGGAGACGGTGCAGCGGCTGTTGCCGGTGCTGTGCCAGGACCATGGCCTGACCCCGGCGCAGGTGGTGGCCAT
CGCCAGCCATATTGGCGGCAAGCAGGCGCTGGAGACGGTGCAGCGGCTGTTGCCGGTGCTGTGCCAGGACCATGGCCTGACCCTGGACCAGGTGG
TGGCCATTGCCAGCAATGACGGCAGCAAGCAGGCGCTGGAGACGGTGCAGCGGCTGTTGCCGGTGCTGTGCCAAGACCATGGCCTGACCCCGGAC
CAGGTCGTGGCCATCGCCAGCCACGATGGCGGCAAGCAGGCGCTGGAGACGGTGCAGCGGCTGTTGCCGGTGCTGTGCCAGGACCATGGCCTGAC
CCCGGACCAGGTCGTGGCCATCGCCAGCCATGGCGGCGGCAAGCAGGCGCTGGAGACGGTGCAACGGCTGTTGCCGGTGCTGTGCCAGGACCATG
GCCTGACCTCGGCCCAGGTCGTGGCCATCGCCAACAATAACGGCGGCAAGCAGGCGCTGGAGACGGTGCAGCGGCTGTTGCCGGTGCTGTGCCAG
GACCATGGCCTGACCCCGGACCAGGTCGTGGCCATCGCCAGCCATGGCGGCGGCAAGCAGGCGCTGGAGACGGTGCAGCGGCTGTTGCCGGTGCT
GTGCCAGGACCATGGCCTGACCCCGGACCAGGTCGTGGCCATCGCCAGCAATGGCGGCAAGCAGGCGCTGGAGAGCATTGTTGCCCAGTTATCTC

GCCCTGATCCGGCGTTGGCCGCGTTGACCAACGACCACCTCGTCGCCTTGGCCTGCCTCGGCGGACGTCCTGCCCTGGATGCAGTGAAAAAGGGA
TTGCCGCACGCGCCGGAATTGATCAGAAGAATCAATCGCCGCATTCCCGAACGCACGTCCCATCACGTTCCCGACCTCGCGCACGTGGTGCGCGT
GCTTGGTTTTTTCCAGAGCCACTCCCACCCAGCGCAAGCATTCGATGACGCCATGACGCAGTTCGGGATGAGCAGGAACGGGTTGGTACAGCTCT
TTCGCAGAGTGGGCGTCACCGAACTCGAAGCCCGCGGTGGAACGCTCCCCCCAGCCTCGCAGCGTTGGGACCGTATCCTCCAGGCATCAGGGATG
AAAAGGGCCAAACCGTCCCCTACTTCAGCTCAAACACCGGATCAGGCGTCTTTGCATGCATTCGCCGATTCGCTGGAGCGTGACCTTGATGCGCC
TAGCCCAATGCACGAGGGAGATCAGACAGGGGCAAGCAGCCGTAAACGGTCCCGATCGGATCGTGCTGTCACCGGCCCCTCCGCACAGCACTCTT
TCGAGGTGCGCGTTCCCGAACAGCACGATGCGCTGCATTTGCCCCTCAGCTGGAGGGTAAAACGCCCGCGTACCAGGATCGGGGGCGGCCTCCCG
GATCCTGGTACGCCCATCGCTGCCGACCTGGCAGCGTCCAGCACCGTGCTGTGGGAACAAGATGCGGCCCCCTTCGCAGGGGCAGCGGATGATTT
CCCGGCATTCAACGAAGAGGAGCTCGCATGGTTGATGGAGCTATTGCCTCAGTCAGGCTCAGTCGGAGGGACGATC
>TalAO3 Xoo PXO83
ATGGATCCCATTCGTTCGCGCACGCCAAGTCCTGCCCGCGAGCTTCTGCCCGGCCCCCAACCGGATAGGGTTCAGCCGACTGCAGATCGGGGGGG
GGCTCCGCCTGCTGGCGGCCCCCTGGATGGCTTGCCCGCTCGGCGGACGATGTCCCGGACCCGGCTGCCATCTCCCCCTGCGCCCTCGCCTGCGT
TCTCGGCGGGCAGCTTCAGCGATCTGCTCCGTCAGTTCGATCCGTCGCTTCTTGATACATCGCTTCTTGATTCGATGCCTGCCGTCGGCACGCCG
CATACAGCGGCTGCCCCAGCAGAGTGGGATGAGGTGCAATCGGGTCTGCGTGCAGCCGATGACCCGCCACCCACCGTGCGTGTCGCTGTCACTGC
CGCGCGGCCGCCGCGCGCCAAGCCGGCCCCGCGACGGCGTGCGGCGCAACCCTCCGACGCTTCGCCGGCCGCGCAGGTGGATCTACGCACGCTCG
GCTACAGTCAGCAGCAGCAAGAGAAGATCAAATCGAAGGTGCGTTCGACAGTGGCGCAGCACCACGAGGCACTGGTGGGCCATGGGTTTACACAC
GCGCACATCGTTGCGCTCAGCCAACACCCGGCAGCGTTAGGGACCGTCGCTGTCAAGTATCAGCACATAATCACGGCGTTGCCAGAGGCGACACA
CGAAGACATCGTTGGCGTCGGCAAACAGTGGTCCGGCGCACGCGCCCTGGAGGCCTTGCTCACGAAGGCGGGGGAGTTGAGAGGTCCGCCGTTAC
AGTTGGACACAGGCCAACTTCTCAAGATTGCAAAACGTGGCGGCGTGACCGCAGTGGAGGCAGTGCATGCATCGCGCAATGCACTGACGGGTGCC
CCCCTGAACCTGACCCCGGACCAAGTGGTGGCCATCGCCAGCAATATTGGCGGCAACCAGGCGCTGGAGACGGTGCAGCGGCTGTTGCCGGTGCT
GTGCCAGGACCATGGCCTGACCCCGGACCAAGTGGTGGCCATCGCCAACAATAACGGCGGCAAGCAGGCGCTGGAGACGGTGCAGCGGCTGTTGC
CGGTGCTGTGCCAGGCCCATGGCCTGACCCCGGACCAAGTGGTGGCCATCGCCAGCAATGGCGGCAAGCAGGCGCTGGAGACGGTGCAGCGGCTG
TTGCCGGTGCTGTGCCAGGCCCATGGCCTGACCCCGAACCAGGTCGTGGCCATCGCCAGCAATGGCGGCGGCAAGCAGGCGCTGGAGACGGTGCA
GCGGCTGTTGCCGGTGCTGTGCCAGGCCCATGGCCTGACCCAGGACCAGGTGGTGGCCATCGCCAGCAATAGTGGCGGCAAGCAGGCGCTGGAGA
CGGTGCAGCGGCTGTTGCCGGTGCTGTGCCAGGCCCATGGCCTGACCCCGGCCCAAGTGGTGGCCATCGCCAGCAATAACGGCGGCAAGCAGGCG
CTGGAGACGGTGCAGCGGCTGTTTCCGGTGCTGTGCCAGGACCATGGCCTGACCCCGGACCAGGTGGTGACCATCGCCAACAATAACGGCGGCAA
GCAGGCGCTGGAGACGGTGCAGCGGCTGTTGCCGGTGCTGTGCCAGGCCCATGGCTTGATCCCGGACCAGGTGGTGGCCATCGCCAACAATAACG
GCGGCAAGCAGGCGCTGGAGACGGTGCAGCGGCTGTTGCCGGTGCTGTGCCAGGCCCATGGCCTGACCCCGGCCCAAGTGGTGGCCATCGCCAGC
AATATTGGCGGCAAGCAGGCGCTGGAGACGGTGCAGCGGCTGTTGCCGGTGCTGTGCCGGGCCCATGGCCTGACCCCGGCCCAAGTGGTGGCCAT
CGCCAACAATAACGGCGGCAAGCAGGCGCTGGAGACGGTGCAGCGGCTGTTGCCGGTGCTGTGCCAGGCCCATGGCCTGACCCCGGATCAAGTGG
TGGCCATCGCCAGCAATATTGGCGGCAAGCAGGCGCTGGAGACGGTGCAGCGCCTGTTGCCGGTGCTGTGCCAGGACCATGGCCTGACCCCGGAC
CAGGTCGTGGCCATCGCCAGCAATGGCGGCAAGCAGGCGCTGGAGACGGTGCAGCGGCTGTTGCCGGTGCTGTGCCAGGACCATGGCCTGACCCC
GGACCAGGTGGTGGCCATCGCCAGCCACGATGGCGGCAAGCAGGCGCTGGAGACGGTGCAGCGGCTGTTGCCGGTGCTGTGCCAGGCCCATGGCC
TGACCCCGGACCAGGTCGTGGCCATCGCCAGCCACGATGGCGGCAAGCAGGCGCTGGAGACGGTGCAGCGGCTGTTGCCGGTGCTGTGCCAGGAC
CATGGCCTGACCCTGGACCAGGTGGTGGCCATCGCCAGCAATATTGGCGGCAAGCAGGCGCTGGAGACGGTGCAGCGGCTGTTGCCGGTGCTGTG
CCAGGACCATGGCCTGACCCCGGACCAGGTCGTGGCCATCGCCAGCAATGGCGGCGGCAAGCAGGCGCTGGAGACGGTGCAACGGCTGTTGCCGG
TGCTGTGCCAGGACCATGGCCTGACCCCGGACCAGGTCGTGGCCATCGCCAGCAATGGCGGCGGCAAGCAGGCGCTGGAGAGCATTGTTGCCCAG
TTATCTCGCCCTGATCCGGCGTTGGCCGCGTTGACCAACGACCACCTCGTCGCCTTGGCCTGCCTCGGCGGACGTCCTGCCCTGGATGCAGTGAA
AAAGGGATTGCCGCACGCGCCGGAATTGATCAGAAGAGTCAATAGCCGTATTGGCGAACGCACGTCCCATCGCGTTGCCGACTACGCGCAAGTGG
TTCGCGTGCTGGAGTTTTTCCAGTGCCACTCCCACCCAGCGTACGCATTTGATGAGGCCATGACGCAGTTCGGGATGAGCAGGCACGGGTTGGTA
CAGCTCTTTCGCAGAGTGGGCGTCACCGAATTCGAAGCCCGCTGCGGAACGCTCCCCCCAGCCTCGCAGCGTTGGGACCGTATCCTCCAGGCATC
AGGGATGAAAAGGGCCAAACCGTCCCCTACTTCAGCTCAAACGCCGGATCAGGCGTCTTTGCATGCATTCGCCGATTCGCTGGAGCGTGACCTTG
ATGCGCCCAGCCCAATGCACGAGGGAGATCAGACGCGGGCAAGCAGCCGTAAACGGTCCCGATCGGATCGTGCTGTCACCGGCCCCTCCGCACAG
CAATCTTTCGAGGTGCGCGTTCCCGAACAGCGCGATGCGCTGCATTTGCCCCTCAGCTGGAGGGTAAAACGCCCGCGTACCAGGATCGGGGGCGG
CCTCCCGGATCCTGGTACGCCCATCGCTGCCGACCTGGCAGCGTCCAGCACCGTGATGTGGGAACAAGATGCGGCCCCCTTCGCAGGGGCAGCGG
ATGATTTCCCGGCATTCAACGAAGAGGAGCTCGCATGGTTGATGGAGCTATTGCCTCAGTCAGGCTCAGTCGGAGGGACGATC
>TalAE4 Xoo PXO83
ATGGATCCCATTCGTTCGCGCACGCCAAGTCCTGCCCGCGAGCTTCTGCCCGGACCCCAACCGGATAGGGTTCAGCCGACTGCAGATCGGGGGGG
GGCTCCGCCTGCTGGCGGCCCCCTGGATGGCTTGCCCGCTCGGCGGACGATGTCCCGGACCCGGCTGCCATCTCCCCCTGCGCCCTCGCCTGCGT
TCTCGGCGGGCAGCTTCAGCGATCCGCTGCGTCAGTTCGATCCGTCGCTTCTTGATACATCGCTTCTTGATTCGATGCCTGCCGTCGGCACGCCG
CATACAGCGGCTGCCCCAGCAGAGTGCGATGAGGTGCAATCGGGTCTGCGTGCAGCCGATGACCCGCCACCCACCGTGCGTGTCGCTGTCACTGC
CGCGCGGCCGCCGCGCGCCAAGCCGGCCCCGCGACGGCGTGCGGCGCAACCCTCCGACGCTTCGCCGGCCGCGCAGGTGGATCTACGCACGCTCG
GCTACAGTCAACAGCAGCAAGAGAAGATGAAACCGAAGGTGCGTTCGACAGTGGCGCAGCACCACGAGGCACTGGTGGGCCATGGGTTTACACAC
GCGCACATCGTTGCGCTCAGCCAACACCCGGCAGCGTTAGGGACCGTTGCTGTCACGTATCAGGACATAATCAGGGCGTTGCCAGAGGCGACACA
CGAAGACATCGTTGGCGTCGGCAAACAGTGGTCCGGCGCACGCACCCTGGAGGCCTTGCTCACGGAGGCGGGGGAGTTGAGAGGTCCGCCGTTAC
AGTTGGACACAGGCCAACTTCTCAAGATTGCAAAACGTGGCGGCGTGACCGCAGTGGAGGCAGTGCATGCATGGCGCAATGCACTGACGGGTGCC
CCCCTGAACCTGACCCCGGACCAAGTGGTGGCCATCGCCAGCAATATTGGCGGCAAGCAGGCGCTGGAGACGGTGCAGCGCCTGTTGCCGGTGCT
GTGCCAGGACCATGGCCTGACCCCGGACCAGGTGGTGGCCATCGCCAGCAATAACGGCGGCAAGCAGGCGCTAGAGACGGTGCAGCGGCTGTTGC
CGGTGCTGTGCCAGGACCATGGCCTGACCCCGGACCAGGTCGTGGCCATCGCCAGCAATATTGGCGGCAAGCAGGCGCTGGAGACGGTGCAGCGG
CTGTTGCCGGTGCTGTGCCAGGACCATGGCCTGACCCCGGACCAGGTCGTGGCCATCGCCAGCCATGGCGGCGGCAAGCAGGCGCTGGAGACGGT
GCAGCGGCTGTTGCCGGTGCTGTGCCAGGACCATGGCCTGACCCCGGACCAGGTCGTGGCCATCGCCAGCCATGGCGGCGGCAAGCAGGCGCTGG
AGACGGTGCAGCGGCTGTTGCCGGTGCTGTGCCAGGACCATGGCCTGACCCCGGACCAGGTGGTGGCCATCGCCAACAATGTTGGCGGCAAGCAG
GCGCTGGAGACGGTGCAGCGGCTGTTGCCGGTGCTGTGCCAGGACCATGGCCTGACCCCGGACCAGGTGGTGGCCATCGCCAGCCATGGCGGCGG
CAAGCAGGCGCTGGAGACGGTGCAGCGGCTGTTGCCGGTGCTGTGCCAGGACCATGGCCTGACCCCGGACCAGGTCGTGGCCATCGCCAGCCACG
ATGGCGGCAAGCAGGCGCTGGAGACGGTGCAGCGGCTGTTGCCGGTGCTGTGCCAGGACCATGGCCTGACCCCGGACCAGGTGGTGGCCATCGCC
AGCCATGGCGGCGGCAAGCAGGCGCTGGAGACAGTGCAACGGCTGTTGCCGGTGCTGTGCCAGGACCATGGCCTGACCCCGGACCAGGTGGTGGC
CATCGCCAGCCACGATGGCGGCAAGCAGGCGCTGGAGACGGTGCAGCGGCTGTTGCCGGTGCTGTGCCAGGACCATGGCCTGACCCTGGACCAGG
TGGTGGCCATCGCCAGCCACGATGGCGGCAAGAAGGCGCTGGAGACGGTGCAGCGGCTGTTGCCGGTGCTGTGCCAGGACCATGGCCTGACCCCG
GACCAGGTGGTGGCCATCGCCAGCCACGATGGCGGCAAGCAGGCGCTGGAAACGGTGCAACGGCTGTTGCCGGTGCTGTGCCAGGACCATGGCCT
GACCCCGGACCAGGTCGTGGCCATCGCCAGCAATGGCGGCGGCAAGCAGGCGCTGGAGAGCATTGTTGCCCAGTTATCTCGCCCTGATCCGGCGT
TGGCCGCGTTGACCAACGACCACCTCGTCGCCTTGGCCTGCCTCGGCGGACGTCCTGCCCTGGATGCAGTGAAAAAGGGATTGCCGCACGCGCCG
GAATTGATCAGAAGAGTCAATAGCCGTATTGGCGAACGCACGTCCCATCGCGTTGCCGACTACGCGCAAGTGGTTCGCGTGCTGGAGTTTTTCCA
GTGCCACTCCCACCCAGCGTACGCATTTGATGAGGCCATGACGCAGTTCGGGATGAGCAGGCACGGGTTGGTACAGCTCTTTCGCAGAGTGGGCG
TCACCGAATTCGAAGCCCGCTGCGGAACGCTCCCCCCAGCCTCGCAGCGTTGGGACCGTATCCTCCAGGCATCAGGGATGAAAAGGGCCAAACCG
TCCCCTACTTCAGCTCAAACGCCGGATCAGGCGTCTTTGCATGCATTCGCCGATTCGCTGGAGCGTGACCTTGATGCGCCCAGCCCAATGCACGA
GGGAGATCAGACGCGGGCAAGCAGCCGTAAACGGTCCCGATCGGATCGTGCTGTCACCGGCCCCTCCGCACAGCAATCTTTCGAGGTGCGCGTTC
CCGAACAGCGCGATGCGCTGCATTTGCCCCTCAGCTGGAGGGTAAAACGCCCGCGTACCAGGATCGGGGGCGGCCTCCCGGATCCTGGTACGCCC

ATCGCTGCCGACCTGGCAGCGTCCAGCACCGTGATGTGGGAACAAGATGCTGCCCCCTTCGCAGGGGCAGCGGATGATTTCCCGGCATTCAACGA
AGAGGAGCTCGCATGGTTGATGGAGCTATTGCCTCAGTCAGGCTCAGTCGGAGGGACGATC
>TalAD5 Xoo PXO83
ATGGATCCCATTCGTTCGCGCACGCCAAGTCCTGCCCGCGAGCTTCTGCCCGGACCCCAACCGGATAGGGTTCAGCCGACTGCAGATCGGGGGG
GGCTCCGCCTGCTGGCGGCCCCCTGGATGGCTTGCCCGCTCGGCGGACGATGTCCCGGACCCGGCTGCCATCTCCCCCTGCGCCCTCGCCTGCGT
TCTCGGCGGGCAGCTTCAGCGATCCGCTGCGTCAGTTCGATCCGTCGCTTCTTGATACATCGCTTTTTGATTCGATGCCTGCCGTCGGCACGCCG
CATACAGCGGCTGCCCCAGCAGAGTGGGATGAGGCGCAATCGGCTCTGCGTGCAGCCGATGACCCGCCACCCACCGTGCGTGTCGCTGTCACTGC
CGCGCGGCCGCCGCGCGCCAAGCCGGCCCCGCGACGGCGTGCGGCGCAACCCTCCGACGCTTCGCCGGCCGCGCAGGTGGATCTACGCACGCTCG
GCTACAGTCAGCAGCAGCAAGAGAAGATCAAACCGAAGGTGCGTTCGACAGTGGCGCAGCACCACGAGGCACTGGTGGGCCATGGGTTTACACAC
GCGCACATCGTTGCGCTCAGCAAACACCCGGCAGCGTTAGGGACCGTTGCTGTCACGTATCAGCACATAATCACGGCGTTGCCAGAGGCGACACA
CGAAGACATCGTTGGCGTCGGCAAACAGTGGTCCGGCGCACGCGCCCTGGAGGCCTTGCTCACGGATGCGGGGGAGTTGAGAGGTCCGCCGTTAC
AGTTGGACACAGGCCAACTTCTCAAGATTGCAAAACGTGGCGGCGTGACCGCAGTGGAGGCAGTGCATGCATCGCGCAATGCACTGACGGGTGCC
CCCCTGAACCTGACCCCGGCACAGGTGGTGGCCATCGCCAGCAATAACGGCGGCAAGCAGGCGCTGGAGACGGTGCAGCGGCTGTTGCCGGTGCT
GTGCCAGGCCCATGGCCTGACCCCGGCGCAGGTGGTGGCCATCGCCAGCCACGATGGCGGCAAGCAGGCACTGGAGACGGTGCAGCGGCTGTTGC
CGGTGCTGTGCCAGGACCATGGCCTGACCCCGGACCAGGTGGTGGCCATCGCCAGCAATAGTGGCGGCAAGCAGGCGCTGGAGACGGTGCAGCGG
CTGTTGCCGGTGCTGTGCCAGGACCATGGCCTGACACCGGACCAGGTGGTGGCCATCGCCAGCAATGGCGGCGGCAAGCAGGCGCTGGAGACGGT
GCAGCGGCTGTTGCCGGTGCTGTGCCAGGACCATGGCCTGACCCCGGACCAGGTGGTGGCCATCGCCAGCCACGATGGCGGCAAGCAGGCGCTGG
AGACGGTGCAGCGGCTGTTGCCGGTGCTGTGCCAGGACCATGGCCTGACCCCGGACCAATTGGTGGCCATCGCCAACAATAACGGCGGCAAGCAG
GCGCTGGAGACGGTGCAGCGGCTGTTGCCGGTGCTGTGCCAGGACCATGGCCTGACCCCGGACCAGGTCGTGGCCATCGCCAGCAATGGCGGCAA
GCAGGCGCTGGAGACAGTGCAGCGGCTGTTGCCGGTGCTGTGCCAGGACCATGGTCTGACCCCGGACCAGGTCGTGGCCATCGCCAGCAATATTG
GCGGCAAGCAGGCGCTGGAGACGGTGCAGCGGCTGTTGCCGGTGCTGTGCCAGGCCCATGGTCTGACCCCGGCGCAGGTGGTGGCCATCGCCAGC
CACGATGGCGGCAAGCAGGCGCTGGAGACGATGCAGCGGCTGTTGCCGGTGCTGTGCCAGGCCCATGGCCTGACCCCGGCGCAGGTGGTGGCCAT
CGCCAGCAATAGCGGCGGCAAGCAGGCGCTGGAGACGGTGCAGCGGCTGTTGCCGGTGCTGTGCCAGGCCCATGGCCTGACCCCGGCGCAGGTGG
TGGCCATCGTCAGCCACGATGGCGGCAAGCAGGCGCTGGAGACGGTGCAGCGGCTGTTGCCGGTGCTGTGCCAGGCCCATGGTCTGACCCCGGAC
CAGGTGGTGGCCATCGCCAGCAATAACGGCGGCAAGCAGGCGCTGGAGACGGTGCAGCGGCTGTTGCCGGTGCTGTGCCAGGACCATGGCCTGAC
CCCGGACCAGGTGGTGGCCATCGCCAGCCACGATGGCGGCAAGCAGGCGCTGGGGACGGTGCAGCGGCTGTTGCCGGTGCTGTGCCAGGACCATG
GCCTGACCCCGGACCAGGTGGTGGCCATCGCCAGCAATAACGGCGGCAAGCAGGCGCTGGAGACGGTGCAGCGGCTGTTGCCGGTGCTGTGCCAG
GACCATGGCCTGACCCCGGACCAGGTGGTGGCCATCGCCAGCCACGATGGCGGTAAGCAGGCGCTGGAGACGGTGCAGCGGCTGTTGCCGGTGCT
GTGCCAGGCCCATGGCCTGACCCCGGCGCAGGTGGTGGCCATCGCCAGCAATAACGGCGGCAAGCAGGCGCTGGAGACGGTGCAGCGGCTGTTGC
CGGTGCTGTGCCAGGACCATGGTCTGACCCCGGACCAGGTGGTAGCCATCGCCAACAATAACGGCGGCAAGCAGGCGCTGGAGACGGTGCAGCGG
CTGTTGCCAGTGCTGTGCCAGGCCCATGGCCTGACCCCGGCGCAGGTGGTGGCCATCGCCAGCAATAACGGCAGCAAGCAGGCGCTGGAGACGGT
GCAGCGGCTGTTGCCGGTGCTGTGCCAGGCCCATGGCCTGACCCCGGCGCAGGTGGTGGCCATCGCCAGCAATAACGGCGGCAAGCAGGCGCTGG
AGACGGTGCAGCGGCTGTTGCCGGTGCTGTGCCAGGCCCATGGTCTGACCCCGGACCAGGTGGTGGCCATCGCCAGCAATAACGGCGGCAAGCAG
GCGCTGGAGACGGTGCAGCGGCTGTTGCCGGTGCTGTGCCAGGCCCATGGCCTGACCCCGGACCAGGTGGTGGCCATCGCCAGCAATAACGGCGG
CAAGCAGGCGCTGGAGACGGTGCAGCGGCTGTTGCCGGTGCTGTGCCAGGCCCATGGCCTGACCCCGGACCAGGTGGTGGCCATCGCCAGCAATA
ACGGCGGCAAGCAGGCGCTGGAGACGGTGCAGCGGCTGTTGCCGGTGCTGTGCCAGGACCATGGCCTGACCCCGGCGCAGGTGGTGGCCATCGCC
AGCCACGATGGCGGCAAGCAGGCGCTGGAGACGGTGCAGCGGCTGTTGCCGGTGCTGTGCCAGGACCATGGCCTGACCCCGGCGCAGGTGGTGGC
CATCGCCAGCAATGGCGGCGGCAAGCAGGCGCTGGAGAGCATTGTTGCCCAGTTATCTCGCCCTGATCCGGCGTTGGCCGCGTTGACCAACGACC
ACCTCGTCGCCTTGGCCTGCCTCGGCGGACGTCCTGCCCTGGATGCAGTGAAAAAAGGATTGCCGCACGCGCCGGAATTGATCAGAAGAGTCAAT
AGCCGTATTGGCGAACGCACGTCCCATCGCGTTGCCGACTACGCGCAAGTGGTTCGCGTGCTGGAGTTTTTCCAGTGCCACTCCCACCCAGCGTA
CGCATTTGATGAGGCCATGACGCAGTTCGGGATGAGCAGGAACGGGTTGTTACAGCTCTTTCGCAGAGTGGGCGTCACCGAACTCGAAGCCCGCG
GTGGAACGCTCCCCCCAGCCTCGCAGCGTTGGGACCGTATCCTCCAGGCATCAGGGATGAAAAGGGCCAAACCGTCCTGTGCTTCGGCTCAAACC
CCGGATCAGGCGTCTTTGCATGCATTCGCCGATTCGCTGGAGCGTGACCTTGATGCGCCCAGCCCAATGCACGAGGGAGATCAGACGCGGGCAAG
CAGCCGTAAACGGTCCCGATCGGATCGTGCTGTCACCGGCCCCTCCGCACAGCAATCTTTCGAGGTGCGCGTTCCCGAACAGCGCGATGCGCTGC
ATTTGCCCCTCAGCTGGAGGGTAAAACGCCCGCGTACCAGGATCGGGGGCGGCCTCCCGGATCCTGGTACGCCCATGGCTGCCGACCTGGCAGCG
TCCAGCACCGTGATGTGGGAACAAGATGCGGCCCCCTTCGCAGGGGCAGCGGATGATTTCCCGGCATTCAACGAAGAGGAGCTCGCATGGTTGAT
GGAGCTATTGCCTCAGTCAGGCTCAGTCGGAGGGACGATC
>TalAI4 Xoo PXO83 (Pseudo)
ATGGATCCCATTCGTTCGCGCACGCCAAGTCCTGCCCGCGAGCCTCTGCCCGGACCCCAACCGGATAGGGTTCAGCCGACTGCAGATCGTGGGGT
GTCTGCGCCTGCTGGCAGCCCTCTGGATGGCTTGCCCGCTCGGCGGACGGTGTCCCGGACCCGGCTGCCATCTCCCCCTGCCCCCTTGCCTGCGT
TCTCGGCGGGCAGCTCCACCGATCGGCTCCGTCCGTTCGATCCGTCGCTTCCTGATACATCGCTTTTTGATTCGATGCCTGCCGTCGGCACGCCT
CATACAGAGGCTGCCCCAGCAGACACTTCGCCGGCCGCGCAGGTGGATCTACTCACGCTCGCGACAGTGGCGCAGCACCACGAGGCACTGGTGGG
CCATGGGTTTACACACGCGCACATCGTTGCGCTCAGCCAACACCCGGCAGCGTTAGGGACCGTTGCTGTCACGTATCAAGACATAATCACGGCGT
TGCCAGAGGCGACACACGAAGACATCGTTGGCGTCGGCAAACAGTTGTCCGGCGCACGCGCCCTGGAGGCCTTGCTCACGAAGGCGGGGGAGTTG
AGAGGTCCGCCGTTACAGTTGGACACAGGCCAACTTCTCAAGATTGCAAGACGTGGCGGCGTGACCGCAGTGGAGGCAGTGCATGCATGGCGCAA
TGCACTGACGGGTGCCCCCCTGAACCTGACCCCGGACCAAGTGGTGGCCATCGCCAGCAATAGTGGCGGCAAGCAGGCGCTGGAGACGGTGCAGC
GGCTGTTGCCGGTGCTGTGCCAGGCCCATGGCCTGACCCCGGACCAGGTCGTGGCCATCGCCAGCAATGGCGGCGGCAAGCAGGCGCTGGAGACG
GTGCAGCGGCTGTTGCCGGTGCTGTGCCAGGACCATGGCCTGACCCCGGACCAGGTGGTGGCCATCGCCAGCAATGGCGGCGGCAAGCAGGCGCT
GGAGACGGTGCAGCGGCTGTTGCCGGTGCTGTGCCAGGACCATGGCCTGACCCCGGACCAGGTGGTGGCCATCGCCAGCAATGGCGGCGGCAAGC
AGGCGCTGGAGACGGTACTGTGCCAGGCCCATGGCCTGACCCCGGCGCAGGTGGTGGCCATCGCCAGCAATGGCGGCGGCAAGCAGGCGCTGGAG
ACGGTGCAGCGGCTGTTGCCGGTGCTGTGCCAGGCCCATGGCCTGACCCTGGACCAGGTCGTGGCCATTGCCAGCAATGGCGGCGGCAAGCAGGC
GCTGGAGACGGTGCAGCGGCTGTTGCCGGTGCTGTGCCAGGCCCATGGCCTGACCCCGGCGCAGGTGGTGGCCATCGCCAGCAATGGCGGCGGCA
AGCAGGCGCTGGAGACGGTGCAGCGGCTGTTGCCGGTGCTGTGCCAGGCCCATGGCCTGACCCCGGCCCAGGTGGTGGCCATCGCCAGCCACGAT
GGCGGCAAGCAGGCGCTGGAGACGGTGCAGCGGCTGTTGCCGGTGCTGTGCCAGGCCCATGGTCTGACCCTGGACCAGGTAGTGGCCATTGCCAG
CCACGATGGCGGCAAGCAGGCGCTGGAGACGGTGCAGCGGCTGTTGCCGGTGCTGTGCCAGGCCCATGGTCTGACCCTGGACCAGGTCGTGGCCA
TCGCCAGCCACGATGGCGGCAAGCAGGCGCTGGAGACGGTGCAGCGGCTGTTGCCGGTGCTGTGCCAGGACCATGGTCTGACCCCGGCGCAGGTG
GTGGCCATCGCCAGCAATAACGGCGGCAAGCAGGCGCTGGAGACGGTGCAGCGGCTGTTGCCGGTGCTGTGCCAGGACCATGGCCTGACCCCGGA
CCAGGTGGTGGCCATCGCCAGCCACGATGGCGGCAAGCAGGCGCTGGAGACGATGCAGCGGCTGTTGCCGGTGCTGTGCCAGGCCCATGGCCTGA
CCCCGGACCAGGTCGTGGCCATCGCCAGCAATGGCGGCGGCAAGCAGGCGCTGGAGACGGTGCAGCGGCTGTTGCCGGTGCTGTGCCAGGCCCAT
GGCCTGACCCCGGACCAGGTCGTGGCCATCGCCAGCCACGATGGCGGCAAGCAGGCGCTGGAGACGGTGCAGCGGCTGTTGCCGGTGCTGTGCCA
GGCCCATGGCCTGACCCCGGACCAGGTCGTGGCCATCGCCAGCCACGATGGCGGCAAGCAGGCGCTGGAGACGGTGCAACGGCTGTTGCAGGTGC
TGTGCCAGGACCATGGCCTGACCCCGGACCAGGTGGTGGCCATCGCCAGCCACGATGGCGGCAAGCAGGCGCTGGAGACGGTGCAACGGCTGTTG
CCGGTGCTGTGCCAGGACCATGGCCTGACCCCGGACCAGGTGGTGGCCATCGCCAGCCACGATGGCGGCAAGCAGGCGCTGGAGACGGTGCAGCG
GCTGTTGCCGGTGCTGTGCCAGGCCCATGGCCTGACCCCGGACCAGGTGGTGGCCATCGCCAGCCATGGCGGCAAGCAGGCGCTGGAGAGCATTG
TTGCCCAGTTATCTCGCCGTGATCCGGCGTTGGCCGCGTTGACCAACGACCAACTCGTCGCCTTGGCCTGCCTCGGCGGACGTCCTGCCCCGCAT
TCAAGGAAGAGGAAATCGCATGATTGA
>TalBA2 Xoo PXO83

ATGGATCCCATTCGTTCGCGTACGCCAAGTCCTGCCCGCGAGCTTCTGCCCGGACCCCAACCGGATAGGGTTCAGCCGACTGCAGATCGGGGGGG
GGCTCCGCCTGCTGGCGGCCCCCTGGATGGCTTGCCCGCTCGGCGGACGATGTCCCGGACCCGGCTGCCATCTCCCCCTGCGCCCTCGCCTGCGT
TCCCGGCGGGCAGCTTCAGCGATCTACTCCGTCAAGTCGATACATCGCTTCTTGATTCGATGCCTGCCGTCGGCACGCCGCATACAGCGGCTGCC
CCAGCAGAGTGGGATGAGATGCAATCGGGTCTGCGTGCAGCCGATGACCCGCCACCCACCGTGCGTGTCGCTGTCACTGCCGCGCGGCCGCCGCG
CGCCAAGCCGGCCCCGCGACGGCGTGCGGCGCAACCCTCCGACGCTTCGCCGGCCGCGCAGGTGGATCTACGCACGCTCGGCTACAGTCAGCAGC
AGCAAGAGAAGATCAAACCGAAGGTGCGTTCGACAGTGGCGCAGCACCACGAGGCACTGGTGGGCCATGGGTTTACACACGCGCACATCGTTGCG
CTCAGCCAACACCCGGCAGCGTTAGGGACCGTTGCTGTCACGTATCAGGACATAATCAGGGCGTTGCCAGAGGCGACACACGAAGACATCGTTGG
CGTCGGCAAACAGTGGTCCGGCGCACGCGCCCTGGAGGCCTTGCTCACGGAGGCGGGGGAGTTGAGAGGTCCGCCGTTACAGTTGGACACAGGCC
AACTTCTCAAGATTGCAAAACGTGGCGGCGTGACCGCAGTGGAGGCAGTGCATGCATGGCGCAATGCACTGACGGGTGCCCCCCTGAACCTGACC
CCGGACCAAGTGGTGGCCATCGCCAGCAATATTGGCGGCAAGCAGGCGCTGGAGACGGTGCAGCGCCTGTTGCCGGTGCTGTGCCAGGCCCATGG
CCTGACCCCGGACCAAGTGGTGGCCATCGCCAGCAATAGTGGCGGCAAGCAGGCGCTGGAGACGGTGCAGCGGCTGTTGCCGGTGCTGTGCCAGG
ACCATGGCCTGACCCCGGACCAGGTGGTGGCCATCGCCAGCCACGATGGCGGCAAGCAGGCGCTGGAGACGGTGCAGCGGCTGTTGCCGGTGCTG
TGCCAGGCCCATGGCCTGACCCCAGACCAGGTCGTGGCCATCGCCAGCCATGGCGGCGGCAAGCAGGCGCTGGAGACGGTGCAGCGGCTGTTGCC
GGTGCTGTGCCAGGCCCATGGCCTGACCCCGGACCAAGTGGTGGCCATCGCCAGCAATAGTGGCGGCAAGCAGGCGCTGGAGACGGTGCAGCGGC
TGTTGCCAGTGCTGTGCCAGGCCCATGGCCTGACCCCGGACCAAGTGGTGGCCATCGCCAACAATAACGGCGGCAAGCAGGCGCTGGAGACGGTG
CAGCGCCTGTTGCCGGTGCTGTGCCAGGACCATGGCCTGAGTCCGGACCAGGTGGTGGCCATCGCCAGCCACGATGGCGGCAAGCAGGCGCTGGA
GACGGTGCAGCGGCTGTTGCCGGTGCTGTGCCAGGACCATGGCCTGACCCCGGACCAGGTCGTGGCCATCGCCAGCCATGGCGGCAAGCAGGCGC
TGGAGACGGTGCAGCGCCTGTTGCCGGTGCTGTGCCAGGACCATGGCCTGACCCCGGACCAGGTGGTGGCCATCGCCAGTAATGGCGGCGGCAAG
CAGGCGCTGGAGACGGTGCAACGGCTGTTGCCGGTGCTGTGCCAGGACCATGGCCTGACCCCGGACCAGGTGGTGGCCATCGCCAACAATAACGG
CGGCAAGCAGGCGCTGGCGACGGTGCAGCGGCTGTTGCCGGTGCTGTGCCAGGCCCATGGCCTGACCCCGGACCAGGTGGTGGCCATCGCCAACA
ATAACGGCGGCAAGCAGGCGCTGGCGACGGTGCAGCGGCTGTTGCCGGTGCTGTGCCAGGCCCATGGCCTGACCCCGGACCAGGTGGTGGCCATC
GCCAGCCACGATGGCGGCAAGCAGGCGCTGGAGACGGTGCAGCGGCTGTTGCCGGTGCTGTGCCAGGCCCATGGCCTGACCCCGGACCAGGTCGT
GGCCATCGCCAGCCACGATGGCGGCAAGCAGGCGCTGGAGACGGTGCAGCGGCTGTTGCCGGTGCTGTGCCAGGCCCATGGCCTGACCCCGGACC
AGGTCGTGGCCATCGCCAGCAATGGCGGCGGCAAGCAGGCGCTGGAGACGGTGCAGCGGCTGTTGCCGGTGCTGTGCCAGGCCCATGGCCTGACC
CCGGACCAGGTCGTGGCCATCGCCAGCCACGATGGCGGCAAGCAGGCGCTGGAGACGGTGCAGCGGCTGTTGCCGGTGCTGTGCCAGGACCATGG
CCTGACCCCGGACCAGGTGGTGGCCATCGCCAGCAATGGCGGCGGCAAGCAGGCGCTGGAGAGCATTGTTGCCCAGTTATCTCGCCCTGATCCGG
CGTTGGCCGCGTTGACCAACGACCACCTCGTCGCCTTGGCCTGCCTCGGCGGACGTCCTGCCCTGGATGCAGTGAAAAAGGGATTGCCGCACGCG
CCGGAATTGATCAGAAGAATCAATCGCCGTATTCCCGAACGCACGTCCCATCGCGTTGCCGACCTCGCGCACGTGGTGCGCGTGCTTGGTTTTTT
CCAGAGCCACTCCCACCCAGCGCAAGCATTCGATGACGCCATGACGCAGTTCGGGATGAGCAGGCACGGGTTGGTACAGCTCTTTCGCAGAGTGG
GCGTCACCGAACTCGAAGCCCGCTGCGGAACGCTCCCCCCAGCCTCGCAGCGTTGGGACCGTATCCTCCAGGCATCAGGGATGAAAAGGGCCAAA
CCGTCCCCTACTTCAGCTCAAACACCGGATCAGGCGTCTTTGCATGCATTCGCCGATTCGCTGGAGCGTGACCTTGATGCGCCCAGCCCAATGCA
CGAGGGAGATCAGACGCGGGCAAGCAGCCGTAAACGGTCCCGATCGGATCGTGCTGTCACCGGCCCCTCCACACAGCAATCTTTCGAGGTGCGCG
TTCCCGAACAGCACGATGCGCTGCATTTGCCCCTCAGCTGGAGGGTAAAACGCCCGCGTACCAGGATCGGGGGCGGCCTCCCGGATCCTGGTACG
CCCATCGCTGCCGACCTGGCAGCGTCCAGCACCGTGATGTGGGAACAAGATGCGGCCCCCTTCGCAGGGGCAGCGGATGATTTCCCGGCATTCAA
CGAAGAGGAGCTCGCATGGTTGATGGAGCTATTGCCTCAGTCAGGCTCAGTTGGAGGGACGATC
>TalAA5 Xoo PXO83
ATGGATCCCATTCGTTCGCGCACGCCAAGTCCTGCCCGCGAGCTTCTGCCCGGACCCCAACCGGATAGGGTTCAGCCGACTGCAGATCGGGGGGG
GGCTCCGCCTGATGGCGGCCCCCTGGATGGCTTGCCCGCTCGGCGGACGATGTCCCGGACCCGGCTGCCATCTCCCCCTGCGCCCTCGCCTGCGT
TCTCGGCGGGCAGCTTCAGCGATCTGCTCCGTCAGTTCGATCCGTCGCTTCTTGATACATCGCTTCTTGATTCGATGCCTGCCGTCGGCACGCCG
CATACAGCGGCTGCCCCAGCAGAGTGGGATGAGGTGCAATCGGGTCTGCGTGCAGCCGATGACCCGCCACCCACCGTGCGTGTCGCTGTCACTGC
CGCGCGGCCGCCGCGCGCCAAGCCGGCCCCGCGACGGCGTGCGGCGCAACCCTCCGACGCTTCGCCGGCCGCGCAGGTGGATCTACGCACGCTCG
GCTACAGTCAGCAGCAGCAAGAGAAGATCAAACCGAAGGTGCGTTCGACAGTGGCGCAGCACCACGAGGCACTGGTGGGCCATGGGTTTACACAC
GCGCACATCGTTGCGCTCAGCCAACACCCGGCAGCGTTAGGGACCGTTGCTGTCACGTATCAGGACATAATCAGGGCGTTGCCAGAGGCGACACA
CGAAGACATCGTTGGCGTCGGCAAACAGTGGTCCGGCGCACGCGCCCTGGAGGCCTTGCTCACGGAGGCGGGGGAGTTGAGAGGTCCGCCGTTAC
AGTTGGACACAGGCCAACTTCTCAAGATTGCAAAACGTGGCGGCGTGACCGCAGTGGAGGCAGTGCATGCATGGCGCAATGCACTGACGGGTGCC
CCCCTGAACCTGACCCCGGACCAAGTGGTGGCCATCGCCAGCAATATTGGCGGCAACCAGGCGCTGGAGACGGTACAGCGGCTGTTGCCGGTGCT
GTGCCAGGCCCATGGCCTGACCCCGGACCAGGTCGTGGCCATCGCCAGCCATGGCGGCGGCAAGCAGGCGCTGGAGACGCTGCAGCGGCTGTTGC
CGGTGCTGTGCCAGGACCATGGCCTGACCCCGGACCAGGTGGTGGCCATCGCCAGCAATAGTGGCGGCAAGCAGGCGCTGGAGACGGTGCAGCGG
CTGTTGCCGGTGCTGTGCCAGGACCATGGCCTGACCCCGGACCAGGTGGTGGCCATCGCCAGCCATGGCGGCGGCAAGCAGGCGCTGGAGACGCT
GCAACGGCTGTTGCCGGTGCTGTGCCAGGACCATGGCCTGACCCCGGACCAGGTGGTGGCCATCGCCAGCCATGGCGGCGGCAAGCAGGCGCTGG
AGACGCTGCAGCGGCTGTTGCCGGTGCTGTGCCAGGCCCATGGCCTGACCCCGGCCCAAGTGGTGGCCCTCGCCAGCCACGATGGCGGCAAGCAG
GCGCTGGAGACGGTGCAGCGGCTGTTGCCGGTGCTGTGCCAGGACCATGGCCTGACCCCGGACCAGGTGGTGGCCATCGCCAGCAATAGTGGCGG
CAAGCAGGCGCTGGAGACGGTGCAGCGGCTGTTGCCGGTGCTGTGCCAGGACCATGGCCTGACCCCGGACCAGGTCGTGGCCATCGCCAGCAATG
GCGGCGGCAAGCAGGCGCTGGAGACGGTGCAACGGCTGTTGCCGGTGCTGTGCCAGGCCCATGGCCTGACCCCGGACCATGTGGTGGCCATCGCC
AGCCACGATGGCGGCAAGCAGGCGCTGGAGACGGTGCAGCGGCTGTTGCCGGTGCTGTGCCAGGACCATGGCCTGACCCCGGACCAGGTCGTGGC
CATCGCCAACAATAACGGCGGCAAGCAGGCGCTGGAGACGGTGCAACGGCTGTTGCCGGTGCTGTGCCAGGCCCATGGCCTGACCCCGGACCAGG
TCGTGGCCATCGCCAGCAATGGCGGCGGCAAGCAGGCGCTGGAGACGGTGCAACGGCTGTTGCCGGTGCTGTGCCAGGACCATGGCCTGACCCCG
GCCCAGGTGGTGGCCATCGCCAGCCATGGCGGCGGCAAGCAGGCGCTGGAGACGGTGCAGCGGCTGTTGCCGGTGCTGTGCCAGGCCCATGGCCT
GACCCCGGACCAGGTCGTGGCCATCGCCAGCAATGGCGGCGGCAAGCAGGCGCTGGAGACGGTGCAGCGGCTGTTGCCGGTGCTGTGCCAGGACC
ATGGCCTGACCCCGGACCAGGTGGTGGCCATCGCCAGCCACGATGGCGGCAAGCAGGCGCTGGAGACGGTGCAGCGGCTGTTGCCGGTGCTGTGC
CAGGACCATGGCCTGACCCCGGACCAGGTGGTGGCCATCGCCAGCCATGGCGGCGGCAAGCAGGCGCTGGAGACAGTGCAGCGGCTGTTGCCGGT
GCTGTGCCAGGACCATGGCCTGACCCCGGACCAGGTGGTGGCCATCGCCAGCCACGATGGCGGCAAGCAGGCGCTGGAGACGGTGCAGCGGCTGT
TGCCGGTGCTGTGCCAGGACCATGGCCTGACCCCGGACCAGGTCGTGGCCATCGCCAGCCACGATGGCGGCAAGCAGGCGCTGGAGACGGTGCAG
CGGCTGTTGCCGGTGCTGTGCCAGGACCATGGCCTGACCCTGGACCAGGTGGTGGCCATCGCCAGCAATATTGGCGGCAAGCAGGCGCTGGAGAC
GGTGCAGCGGCTGTTGCCGGTGCTGTGCCAGGACCATGGCCTGACCCCGGCCCAGGTGGTGGCCATCGCCAACAATAACGGCGGCAAGCAGGCGC
TGGAGACGGTGCAGCGGCTGTTGCCGGTGCTGTGCCAGGACCATGGCCTGACCCCGGACCAGGTGGTGGCCATCGCCAGCAATGGCGGCGGCAAG
CAGGCGCTGGAGAGCATTGTTGCCCAGTTATCTTGCCCTGATCCGGCGTTGGCCGCGTTGACCAACGACCACCTCGTCGCCTTGGCCTGCCTCGG
CGGACGTCCTGCCCTGGATGCAGTGAAAAAGGGATTGCCGCACGCGCCGGAATTGATCAAAAGAATCAATCGCCGCATTCCCGAACGCACGTCCC
ATCGCGTTGCCGACCTCCCCGAACGCACGTCCCATCGCGTTGCCGACCTCGCGCACGTGGTGCGCGTGCTTGGTTTTTTCCAGAGCCACTCCCAC
CCAGCGCAAGCATTCGATGACGCCATGACGCAGTTCGGGATGAGCAGGCACGGGTTGGTACAGCTCTTTCGCAGAGTGGGCGTCACCGAATTCGA
AGCCCGCTGCGGAACGCTCCCCCCAGCCTCGCAGCGTTGGGACCGTATCCTCCAGGCATCAGGGATGAAAAGGGCCAAACCGTCCCCTACTTCAG
CTCAAACACCGGATCAGGCGTCTTTGCATGCATTCGCCGATTCGCTGGAGCGTGACCTTGATGCGCCCAGCCCAATGCACGAGGGAGATCAGACG
CGGGCAAGCAGCCGTAAACGGTCCCGATCGGATCGTGCTGTCACCGGCCCCTCCGCACAGCAATCTTTCGAGGTACGCGTTCCCGAACAGCGCGA
TGCGCTGCATTTGCCCCTCAGCTGGAGGGTAAAACGCCCGCGTACCAGGATCGGGGGCGGCCTCCCGGATCCTGGTACGCCCATCGCTGCCGACC
TGGCAGCGTCCAGCACCGTGCTGTGGGAACAAGATGCGGCCCCCTTCGCAGGGGCAGCGGATGATTTCCCGGCATTCAACGAAGAGGAGCTCGCA
TGGTTGATGGAGCTATTGCCTCAGTCAGGCTCAGTCGGAGGGACGATC
>TalAR3 Xoo PXO83

ATGGATCCCATTCGTTCGCGCACGCCAAGTCCTGCCCGCGAGCTTCTGCCCGGACCCCAACCGGATAGGGTTCAGCCGACTGCAGATCGGGGGGG
GACTCCGCCTGCTGGCGGCCCCCTGGATGGCTTGCCCGTTCGGCGGACGATGTCCCGGACCCGGCTGCCATCTCCCCCTGCACCCTCGCCTGCGT
TCTCGGCGGGCAGCTTCAGCGATCTGCTCCGTCAGTTCGATCCGTCGCTTCTTGATACATCGCTTCTTGATTCGATGCCTGCCGTCGGCACGCCG
CATACAGCGGCTGCCCCAGCAGAGTGGGATGAGGTGCAATCGGGTCTGCGTGCAGCCGATGACCCGCCACCCACCGTGCGTGTCGCTGTCACTGC
CGCGCGGCCGCCGCGCGCCAAGCCGGCCCCGCGACGGCGTGCGGCGCAACCCTCCGACGCTTCGCCGGCCGCGCAGGTGGATCTACGCACGCTCG
GCTACAGTCAGCAGCAGCAAGAGAAGATCAAACCGAAGGTGCGTTCGACAGTGGCGCAGCACCACGAGGCACTGGTGGGCCATGGGTTTACACAC
GCGCACATCGTTGCGCTCAGCCAACACCCGGCAGCGTTAGGGACCGTTGCTGTCACGTATCAGGACATAATCAGGGCGTTGCCAGAGGCGACACA
CGAAGACATCGTTGGCGTCGGCAAACAGTGGTCCGGCGCACGCGCCCTGGAGGCCTTGCTCACGGAGGCGAGGGAGTTGAGAGGTCCGCCGTTAC
AGTTGGACACAGGCCAACTTCTCAAGATTGCAAAACGTGGCGGCGTGACCGCAGTGGAGGCAGTGCATGCATGGCGCAATGCACTGACGGGTGCC
CCCCTGAACCTGACCCCGGACCAAGTGGTGGCCATCGCCAGCAATATTGGCGGCAACCAGGCGCTGGAGACGGTACAGCGGCTGTTGCCGGTACT
GTGCCAGGACCATGGCCTGACCCCGGACCAGGTCGTGGCCATCGCCAGCCATGGCGGCAAGCAGGCGCTGGAGACGGTGCAGCGGCTGTTGCCGG
TGCTGTGCCAGGACCATGGCCTGACCCCGGACCAGGTGGTGGCCATCGCCAGCAATATTGGCGGCAAGCAGGCGCTGGAGACGGTGCAGCGGCTG
TTGCCGGTGCTGTGCCAGGCCCATGGCCTGACCCCGGCCCAGGTGGTGGCCATCGCCAGCAATAACGGCGGCAAGCAGGCGCTGGAGACGGTGCA
GCGGCTGTTGCCGGTGCTGTGCCAGGCCCATGGCCTGACCCCGGACCAGGTGGTGGCTATCGCCAGCAATAACGGCGGCAAGCAGGCGCTGGAGA
CGGTGCAACGGCTGTTGCCGGTGCTGTGCCAGGCCCATGGCCTGACCCCGGACCAAGTGGTGGCCATCGCCAACAATAACGGCGGCAAGCAGGCG
CTGGAGACGCTGCAGCGGCTGTTGCCGGTGCTGTGCCAGGACCATGGCCTGAGTCCGGACCAGGTGGTGGCCATCGCCAACAATAACGGCGGCAA
GCAGGCGCTGGAGACGCTGCAGCGGCTGTTGCCGGTGCTGTGCCAGACCCATGCCCTGACCCCGGACCAGGTGGTGGCCATCGCCAACAATAACG
GCGGCAAGCAGGCGCTGGAGACGGTGCAGCGGCTGTTGCCGGTGCTGTGCCAGGCCCATGGCCTGACCCCGGACCAGGTGGTGGCCATCGCCAGC
CACGATGGCGGCAAGCAGGCGCTGGAGACGGTGCAGCGGCTGTTGCCGGTGCTGTGCCAGGACCATGGCCTGACCCCGGACCAGGTGGTGGCCAT
CGCCAGCAATATTGGCGGCAAGCAGGCGCTGGAGACGGTGCAGCGGCTGTTGCCGGTGCTGTGCCAGGACCATGGCCTGACCCCGGACCAGGTCG
TGGCCATCGCCAGCAATAACGGCGGCAAGCAGGCGCTGGAGACGGTGCAGCGGCTGTTGCCGGTGCTGTGCCAGGACCATGGCCTGACCCCGGAC
CAGGTGGTGGCCATCGCCAGCCATGGCGGCGGCAAGCAGGCGCTGGAGACGGTGCAGCGGCTGTTGCCGGTGCTGTGCCAGGCCCATGGCCTGAC
CCCAGACCAGGTCGTGGCCATCGCCAGCCACGATGGCGGCAAGCAGGCGCTGGAGACGGTGCAGCGGCTGTTGCCGGTGCTGTGCCAGGCCCATG
GCCTGACCCCGAACCAGGTGGTGGCCATCGCCAGCAATATTGGCGGCAAGCAGGCGCTGGAGACGGTGCAACGGCTGTTGCCGGTGCTGTGCCAG
GACCATGGCCTGACCCCGGACCAGGTGGTGGCCATCGCCAGCAATGGCGGCAAGCAGGCGCTGGAGACGGTGCAGCGGCTGTTGCCGGTGCTGTG
CCAGGCTCATGGCCTGACCCCGGACCAAGTGGTGGCCATCGCCAGTAATAGTGGCGGCAAGCAGGCGCTGGAGACGGTGCAGCGGCTGTTGCCGG
TGCTGTGCCAGGACCATGGCCTGACCCCGAACCAAGTGGTGGCCATCGCCAGCAATATTGGCGGCAAGCAGGCGCTGGAGACGGTGCAGCGGCTG
TTGCCGGTGCTATGCCAGGACCATGGCCTGACCCCGGACCAGGTGGTGGCCATCGCCAGCAATATTGGCGGCAAGCAGGCGCTGGAGACGGTGCA
GCGGCTGTTGCCGGTGCTGTGCCAGGACCATGGCCTGACCCTGGACCAGGTGGTGGCCATCGCCAGCCACGATGGCGGCAAACAGGCGCTGGAGA
CGGTGCAACGGCTGTTGCCGGTGCTGTGCCAGGCCCATGGCCTGACCCCGTACCAGGTGGTGGCCATCGCCAGCAATGGCGGCAAGCAGGCGCTG
GAGACGGTGCAGCGGCTGTTGCCGGTGCTGTGCCAGGCCCATGGCCTGACCCCGAACCAGGTGGTGGCCATCGCCAGCAATAGTGGCGGCAAGCA
GGCGCTGGAGACGGTGCAGCGGCTGTTGCCGGTGCTGTGCCAGGACCATGGCCTGACCCCGAACCAGGTGGTGGCCATCGCCAGCAATATTGGCG
GCAAGCAGGCGCTGGAGACGGTGCAGCGGCTGTTGCCGGTGCTGTGCCAGGACCATGGCCTGACCCCGAACCAGGTGGTGGCCATCGCCAGCAAT
GGCGGCGGCAAGCAGGCGCTGGAGAGCATTGTTGCCCAGTTATCTTGCCCTGATCCGGCGTTGGCCGCGTTGACCAACGACCACCTCGTCGCCTT
GGCCTGCCTCGGCGGACGTCCTGCCCTGGATGCAGTGAAAAAGGGATTGCCGCACGCGCCGGAATTGATCAAAAGAATCAATCGCCGCATTCCCG
AACGCACGTCCCATCGCGTTCCCGACCTCGCGCACGTGGTTCGCGTGCTTGGTTTTTTCCAGAGCCACTCCCACCCAGCGCAAGCATTCGATGAC
GCCATGACGCAGTTCGGGATGAGCAGGCACGGCTTGGTACAGCTCTTTCGCAGAGTGGGCGTCACCGAATTCGAAGCCCGCTACGGAACGCTCCC
CCCAGCCTCGCAGCGTTGGGACCGTATCCTCCAGGCATCAGGGATGAAAAGGGCCAAACCGTCCCCTACTTCAGCTCAAACACCGGATCAGGCGT
CTTTGCATGCATTCGCCGATTCGCTGGAGCGTGACCTTGATGCGCTCAGCCCAATGCACGAGGGAGATCAGACGCGGGCAAGCAGCCGTAAACGG
TCCCGATCGGATCGTGCTGTCACCGGCCCCTCCGCACAGCAATCTTTCGAGGTGCGCGTTCCCGAACAGCGCGATGCGCTGCATTTGCCCCTCAG
CTGGAGGGTAAAACGCCCGCGTACCAGGATCGGGGGCGGCCTCCCGGATCCTGGTACGCCCATCGCTGCCGACCTGGCAGCGTCCAGCACCGTGA
TGTGGGAACAAGATGCGGCCCCCTTCGCAGGGGCAGCGGATGATTTCCCGGCATTCAACGAAGAGGAACTCGCATGGTTGATGGAGCTATTGCCT
CAGTCAGGCTCAGTTGGAGGGACGATC

>TalAG4 Xoo PXO83

ATGGATCCCATTCGTTCGCGCACGCCAAGTCCTGCCCGCGAGCTTCTGCCCGGACCCCAACCGGATAGGGTTCAGCCGACTGCAGATCGGGGGGG
GGCTCCGCCTGCTGGCGGCCCCCTGGATGGCTTGCCCGCTCGGCGGACGATGTCCCGGACCCGGCTGCCATCTCCCCCTGCGCCCTCGCCTGCGT
TCTCGGCGGGCAGCTTCAGCGATCTGCTCCGTCAGTTCGATCCGTCGCTTCTTGATACATCGCTTCTTGATTCGATGCCTGCCGTCGGCACGCCG
CATACAGCGGCTGCCCCAGCAGAGTGCGATGAGGTGCAATCGGGTCTGCGTGCAGCCGATGACCCGCCACCCACCGTGCGTGTCGCTGTCACTGC
CGCGCGGCCGCCGCGCGCCAAGCCGGCCCCGCGACGGCGTGCGGCGCAACCCTCCGACGCTTCGCCGGCCGCGCAGGTGGATCTACGCACGCTCG
GCTACAGTCAGCAGCAGCAAGAGAAGATCAAACCGAAGGTGCGTTCGACAGTGGCGCAGCACCACGAGGCACTGGTGGGCCATGGGTTTACACAC
GCGCACATCGTTGCGCTCAGCCAACACCCGGCAGCGTTAGGGACCGTTGCTGTCACGTATCAGGACATAATCACGGCGTTGCCAGAGGCGACACA
CGAAGACATCGTTGGCGTCGGCAAACAGTGGTCCGGCGCACGCGCCCTGGAGGCCTTGCTCACGGAGGCGAGGGAGTTGAGAGGTCCGCCGTTAC
AGTTGGACACAGGCCAACTTCTCAAGATTGCAAAACGTGGCGGCGTGACCGCAGTGGAGGCAGTGCATGCATGGCGCAATGCACTGACGGGTGCC
CCCCTGAACCTGCCCCCGGACCAAGTGGTGGCCATCGCCAGCAATATTGGCGGCAAGCAGGCGCTGGAGACGGTGCAGCGGCTGTTGCCGGTGCT
GTGCCAGGCCCATGGCCTGACCCCGGACCAGGTCGTGGCCATCGCCAGCAATGGCGGCGGCAAGCAGGCGCTGGAGACGGTGCAGCGGCTGTTGC
CGGTGCTGTGCCAGGCCCATGGCCTGACCCCGGACCAGGTCGTGGCCATCGCCAGCAATAACGGCGGCAAGCAGGCGCTGGAGACGGTGCAGCGG
CTGTTGCCGGTGCTGTGCCAGGACCATGGCCTGACCCCGGACCAGGTCGTGGCCATCGCCAGCAATGGCGGCGGCAAGCAGGCGCTGGAGACGGT
GCAGCGGCTGTTGCCGGTGCTGTGCCAGGACCATGGCCTGACCCCGGACCAAGTGGTGGCCATCGCCAACAATAAAGGCGGCAAGCAGGCGCTGG
AGACGCTGCAGCGGCTGTTGCCGGTGCTGTGCCAGGCCCATGGCCTGACCCCGGACCAGGTGGTGGCCATCGCCAGCAATGGCGGCGGCAAGCAG
GCGCTGGAGACGGTGCAACGGCTGTTGCCGGTGCTGTGCCAGGATCATGGCCTGACCCCGGCCCAGGTCGTGGCCATCGCCAGCAATATTGGCGG
CAAGCAGGCGCTGGAGACGGTGCAGCGGCTGTTGCCGGTGCTGTGCCAGGACCATGGCCTGACCCCGGACCAAGTGGTGGCCATCGCCAACAATA
ACGGCGGCAAGCAGGCGCTGGAGACGGTGCAGCGGCTGTTGCCGGTGCTGTGCCAGGACCATGGCCTGACCCCGGACCAGGTCGTGGCCATCGCC
AGCAATATTGGCGGCAAGCAGGCGCTGGAGATGGTGCAGCGGCTGTTGCCGGTGCTGTGCCAGGACCATGGCCTGACCCCGGACCAAGTGGTGGC
CATCGCCAACAATAACGGCGGCAAGCAGGCGCTGGAGACGGTGCAGCGGCTGTTGCCGGTGCTGTGCCAGGCCCATGGCCTGACCCCGGACCAGG
TCGTGGCCATCGCCAGCAATATTGGCGGCAAGCAGACGCTGGAGACGGTGCAGCGGCTGTTGCCGGTGCTGTGCCAGGACCATGGCCTAACCCCG
GACCAGGTCGTGGCCATCGCCAACAATAACGGCGGCAAGCAGGCGCTGGAGACGGTGCAGCGGCTGTTGCCGGTGCTGTGCCAGGACCATGGCCT
GACCCCGGACCAGGTCGTGGCCATCGCCAGCAATAGTGGCGGCAAGCAGGCGCTGGAGACGGTGCAGCGGCTGTTGCCGGTGCTGTGCCAGGACC
ATGGCCTGACCCCGAACCAGGTGGTGGCCATCGCCAGCAATGGCGGCGGCAAGCAGGCGCTGGAGACGGTGCAGCGGCTGTTGCCGGTGCTGTGC
CAGGACCATGGCCTGACCCCGGACCAGGTCGTGGGCATCGCCAGCAATAGTGGCGGCAAGCAGGCGCTGGAGACGGTGCAGCGGCTGTTGCCGGT
GCTGTGCCAGGACCATGGCCTGACCCCGGACCAGGTCGTGGCCATCGCCAACAATAACGGTGGCAAGCAGGCGCTGGAGACGGTGCAGCGGCTGT
TGCCGGTGCTGTGCCAGGACCATGGCCTGACCCCGGACCAGGTGGTGGCCATCGCCAGCAATATTGGCGGCAAGCAGGCGCTGGAGACGGTGCAG
CGGCTGTTGCCGGTGCTGTGCCAGGACCATGGCCTGACCCTGGACAAGGTGGTGGCCATCGCCAGCAATGGCGGCAAGCAGGCGCTGGAGACGGT
GCAGCGGCTGTTGCCGGTGCTGTGCCAGGACCATGGCCTGACCCCGGACCAGGTCGTGGCCATCGCCAGCAATAGTGGCGGCAAGCAGGCGCTGG
AGACGGTGCAGCGGCTGTTGCCGGTGCTGTGCCAGGACCATGGCCTGACCCCGGACCAGGTCGTGGCCATCGCCAGCAATGGCGGCGGCAAGCAG
GCGCTGGAGAGCATTGTTGCCCAGTTATCTCGCCCTGATCCGGCGTTGGCCGCGTTGACCAACGACCACCTCGTCGCCTTGGCCTGCCTCGGCGG
ACGTCCTGCCATGGATGCAGTGAAAAAGGGATTGCCGCACGCGCCGGAATTGATCAGAAGAGTCAATCGCCGTATTGGCGAACGCACGTCCCATC
GCGTTGCCGACTACGCGCAAGTGGTTCGCGTGCTGGAGTTTTTCCAGTGCCACTCCCACCCAGCGTACGCATTTGATGAGGCCATGACGCAGTTC

GGGATGAGCAGGAACGGGTTGGTACAGCTCTTTCGCAGAGTGGGCGTCACCGAACTCGAAGCCCGCGGTGGAACGCTCCCCCCAGCCTCGCAGCG
TTGGGACCGTATCCTCCAGGCATCAGGGATGAAAAGGGCCAAACCGTCCCCTACTTCAGCTCAAACACCGGATCAGGCGTCTTTGCATGCATTCG
CCGATTCGCTGGAGCGTGACCTTGATGCGCCTAGCCCAATGCACGAGGGAGATCAGACAGGGGCAAGCAGCCGTAAACGGTCCCGATCGGATCGT
GCTGTCACCGGCCCCTCCGCACAGCAATCTTTCGAGGTGCGCGTTCCCGAACAGCGCGATGCGCTGCATTTGCCCCTCAGCTGGAGGGTAAAACG
CCCGCGTACCAGGATCGGGGGCGGCCTCCCGGATCCTGGTACGCCCATCGCTGCCGACCTGGCAGCGTCCAGCACCGTGCTGTGGGAACAAGATG
CGGCCCCCTTCGCAGGGGCAGCGGATGATTTCCCGGCATTCAACGAAGAGGAGCTCGCATGGTTGATGGAGCTATTGCCTCAGTCAGGCTCAGTC
GGAGGGACGATC
>TalAC5 Xoo PXO83
ATGGATCCCATTCGTTCGCGCACGCCAAGTCCTGCCCGCGAGCTTCTGCCCGGACCCCAACCGGATAGGGTTCAGCCGACTGCAGATCGGGGGGG
GGCTCCGCCTGCTGGCGGCCCCCTGGATGGCTTGCCCGCTCGGCGGACGATGTCCCGGACCCGGCTGCCATCTCCCCCTGCGCCCTCGCCTGCGT
TCTCGGCGGGCAGCTTCAGCGATCTGCTCCGTCAGTTCGATCCGTCGCTTCTTGATACATCGCTTCTTGATTCGATGCCTGCCGTCGGCACGCCG
CATACAGCGGCTGCCCCAGCAGAGTGGGATGAGGTGCAATCGGGTCTGCGTGCAGCCGATGACCCGCCACCCACCGTGCGTGTCGCTGTCACTGC
CGCGCGGCCGCCGCGCGCCAAGCCGGCCCCGCGACGGCGTGCGGCGCAACCCTCCGACGCTTCGCCGGCCGCGCAGGTGGATCTACGCACGCTCG
GCTACAGTCAGCAGCAGCAAGAGAAGATCAAACCGAAGGTGCGTTCGACAGTGGCGCAGCACCACGAGGCACTGGTGGGCCATGGGTTTACACAC
GCGCACATCGTTGCGCTCAGCCAACACCCGGCAGCGTTAGGGACCGTCGCTGTCAAGTATCAGCACATAATCACGGCGTTGCCAGAGGCGACACA
CGAAGACATCGTTGGCGTCGGCAAACAGTGGTCCGGCGCACGCGCCCTGGAGGCCTTGCTCACGGAGGCGAGGGAGTTGAGAGGTCCGCCGTTAC
AGTTGGACACAGGCCAACTTCTCAAGATTGCAAAACGTGGCGGCGTGACCGCAGTGGAGGCAGTGCATGCATGGCGCAATGCACTGACGGGTGCC
CCCCTGAACCTGACCCCGGACCAAGTGGTGGCCATCGCCAGCAATATTGGCGGCAAGCAGGCGCTGGAGACGGTACAGCGGCTGTTGCCGGTGCT
GTGCCAGGACCATGGCCTGACCCCGGACCAGGTCGTGGCCATCGCCAGCCATGGCGGCGGCAAGCAGGCGCTGGAGACGGTGCAGCGGCTGTTGC
CGGTGCTGTGCCAGGACCATGGCCTGACCCCGGACCAGGTGGTGGCCATCGCCAGCAATATTGGCGGCAAGCAGGCGCTAGAGACGGTGCAGCGG
CTGTTGCCGGTGCTGTGCCAGGCCCATGGCCTGACCCCGGACCAGGTCGTGGCCATCGCCAGCAATATTGGCGGCAAGCAGGCGCTGGAGACGGT
GCAGCGGCTGTTGCCGGTGCTGTGCCAGGACCATGGCCTGACCCCGGCCCAGGTGGTGGCCATCGCCAGCAATAGTGGCGGCAAGCAGGCGCTGG
AGACGGTGCAGCGGCTGTTGCCGGTGCTGTGCCAGGACCATGGCCTGACCCCGGACCAAGTCGTGGCCATCGCCAGCCACGATGGCGGCAAGCAG
GCGCTGGAGACGGTGCAGCGGCTGTTGCCGGTGCTGTGCCAGGACCATGGCCTGACCCCGGACCAGGTCGTGGCCATCGCCAACAATAACGGCGG
CAAGCAGGCGCTGGAGACGCTGCAGCGGCTGTTGCCGGTGCTGTGCCAGGACCATGGCCTGACCCCGGACCAAGTGGTGGCCATCGCCAGCCACG
ATGGCGGCAAGCAGGCGCTGGAGACGGTGCAGCGGCTGTTGCCGGTGCTGTGCCAGGACCATGGCCTGACCCCGGACCAGGTGGTGGCCATCGCC
AGCCACGATGGCGGCAAGCAGGCGCTGGAGACGGTGCAGCGGCTGTTGCCGGTGCTGTGCCAGGACCATGGCCTGACCCCGGCCCAAGTGGTGGC
CATCGCCAGCCACGATGGCGGCAAGCAGGCGCTGGAGACGGTGCAGCGGCTGTTGCCGGTGCTGTGCCAGGACCATGGCCTGACCCCGGACCAGG
TGGTGGCCATCGCCAGCAATAGCGGCGGCAAGCAGGCGCTGGAGACGGTACAGCGGCTGTTGCCGGTGCTGTGCCAGGACCATGGACTGACCCCG
GACCAGGTCGTGGCCATCGCCAGCAATGGCGGCAAGCAGGCGCTGGAGACGGTACAGCGGCTGTTGCCGGTGCTGTGCCAGGACCATGGCCTGAC
CCCGGACCAGGTCGTGGCCATCGCCAGCAATGGCGGCAAGCAGGCGCTGGAGACGGTGCAGCGGCTGTTGCCGGTACAGCGGCTGTTGCCGGTGC
TGTGCCAGGACCATGGCCTGACCCAGGACCAGGTGGTGGCCATCGCCAGCCACGATGGCGGCAAGCAGGCGCTGGAGACGGTGCAGCGGCTGTTG
CCGGTGCTGTGCCAGGACCATGGCCTGACCCCGGACCAAGTGGTGGCCATCGCCAGCCACGATGGCGGCAAACAGGCGCTGGAGACGGTGCAGCG
GCTGTTGCCGGTGCTGTGCCAGGACCATGGCCTGACCCCGGACCAGGTGGTGGCCATCGCCAGCAATAGTGGCGGCAAGCAGGCGCTGGAGACGG
TGCAGCGGCTGTTGCCGGTGCTGTGCCAGGACCATGGCCTGACCCCGGACCAAGTGGTGGCCATCGCCAGCAATAGTGGCGGCAAGCAGGCGCTG
GAGACGGTGCAGCGGCTGTTGCCGGTGCTGTGCCAGGACCATGGCCTGACCCCGGACCAGGTGGTGGCCATCGCCAGCAATAACGGCGGCAAGCA
GGCGCTGGAGACGGTGCAGCGGCTGTTGCCGGTGCTGTGCCAGGACCATGGCCTGACCCCGGACCAGGTCGTGGCCATCGCCAACAATAACGGCG
GCAAGCAGGCGCTGGAGACGGTGCAGCGGCTGTTGCCGGTGCTGTGCCAGGACCATGGCCTGACCCCGGCGCAGGTGGTGGCCATCGCCAGCAAT
ATTGGCGGCAAGCAGGCGCTGGAGACGGTGCAGCGGCTGTTGCCGGTGCTGTGCCAGGACCATGGCCTGACCCTGGACCAGGTGGTGGCCATTGC
CAGCAATGGCGGCAGCAAACAGGCGCTAGAGACGGTGCAGCGGCTGTTGCCGGTGCTGTGCCAGGACCATGGCCTGACCCCGGACCAAGTGGTGG
CCATCGCCAACAATAACGGCGGCAAGCAGGCGCTGGAGACGGTGCAGCGGCTGTTGCCGGTGCTGTGCCAGGACCATGGCCTGACCCCGGACCAG
GTCGTGGCCATCGCCAGCAATATTGGCGGCAAGCAGGCGCTGGAGACGGTGCAGCGGCTGTTGCCGGTGCTGTGCCAGGACCATGGCCTGACCCT
GGACCAGGTGGTGGCCATCGCCAGCAATGGCGGCAAGCAGGCGCTGGAGACGGTGCAGCGGCTGTTGCCGGTGCTGTGCCAGGACCATGGCCTGA
CCCCGAACCAGGTGGTGGCCATCGCCAGCAATAGTGGCGGCAAGCAGGCGCTGGAGACGGTGCAGCGGCTGTTGCCGGTGCTGTGCCAGGACCAT
GGCCTGACCCCGAACCAGGTGGTGGCCATCGCCAGCAATGGCGGCAAGCAGGCGCTGGAGAGCATTGTTGCCCAGTTATCTCGCCCTGATCCGGC
GTTGGCCGCGTTGACCAACGACCACCTCGTCGCCTTGGCCTGCCTCGGCGGACGTCCTGCCCTGGATGCAGTGAAAAAGGGATTGCCGCACGCGC
CGGAATTGATCAGAAGAATCAATCGCCGCATTCCCGAACGCACGTCCCATCGCGTTCCCGACCTCGCGCACGTGGTTCGCGTGCTTGGTTTTTTC
CAGAGCCACTCCCACCCAGCGCAAGCATTCGATGACGCCATGACGCAGTTCGAGATGAGCAGGCACGGCTTGGTACAGCTCTTTCGCAGAGTGGG
CGTCACCGAATTCGAAGCCCGCTACGGAACGCTCCCCCCAGCCTCGCAGCGTTGGGACCGTATCCTCCAGGCATCAGGGATGAAAAGGGCCAAAC
CGTCCCCTACTTCAGCTCAAACACCGGATCAGGCGTCTTTGCATGCATTCGCCGATTCGCTGGAGCGTGACCTTGATGCGCCTAGCCCAATGCAC
GAGGGAGATCAGACAGGGGCAAGCAGCCGTAAACGGTCCCGATCGGATCGTGCTGTCACCGGCCCCTCCGCACAGCAATCTTTCGAGGTGCGCGT
TCCCGAACAGCGCGATGCGCTGCATTTGCCCCTCAGCTGGAGGGTAAAACGCCCGCGTACCAGGATCGGGGGCGGCCTCCCGGATCCTGGTACGC
CCATCGCTGCCGACCTGGCAGCGTCCAGCACCGTGATGTGGGAACAAGATGCGGCCCCCTTCGCAGGGGCAGCGGATGATTTCCCGGCATTCAAC
GAAGAGGAGCTCGCATGGTTGATGGAGCTATTGCCTCAGTCAGGCTCAGTCGGAGGGACGATC
>TalAS3 Xoo PXO83
ATGGATCCCATTCGTTCGCGCACGCCAAGTCCTGCCCGCGAGCTTCTGCCCGGACCCCAACCGGATAGGATTCAGCCGACTGCAGATCGGGGGGG
GGCTCCGCCTGCTGGCGGCCCCCTGGATGGCTTGCCCGCTCGGCGGACGATGTCCCGGACCCGGCTGCCATCTCCCCCTGCGCCCTCGCCTGCGT
TCTCGGCGGGCAGCTTCAGCGATCTGCTCCGTCAGTTCGATCCGTCGCTTCTTGATACATCGCTTCTTGATTCGATGCCTGCCGTCGGCACGCCG
CATACAGCGGCTGCCCCAGCAGAGTGCGATGAGGTGCAATCGGGTCTGCGTGCAGCCGATGACCCGCCACCCACCGTGCGTGTCGCTGTCACTGC
CGCGCGGCCGCCGCGCGCCAAGCCGGCCCCGCGACGGCGTGCGGCGCAACCCTCCGACGCTTCGCCGGCCGCGCAGGTGGATCTACGCACGCTCG
GCTACAGTCAGCAGCAGCAAGAGAAGATCAAACCGAAGGTGCGTTCGACAGTGGCGCAGCACCACGAGGCACTGGTGGGCCATGGGTTTACACAC
GCGCACATCGTTGCGCTCAGCCAACACCCGGCAGCGTTAGGGACCGTCGCTGTCAAGTATCAGCACATAATCACGGCGTTGCCAGAGGCGACACA
CGAAGACATCGTTGGCGTCGGCAAACAGTGGTCCGGCGCACGCGCCCTGGAGGCCTTGCTCACGAAGGCGGGGGAGTTGAGAGGTCCGCCGTTAC
AGTTGGACACAGGCCAACTTCTCAAGATTGCAAAACGTGGCGGCGTGACCGCAGTGGAGGCAGTGCATGCATGGCGCAATGCACTGACGGGTGCC
CCCCTGAACCTGACCCCGGACCAAGTGGTGGCCATCGCCAGCAATATTGGCGGCAAGCAGGCGCTGGAGACGGTGCAGCGGCTGTTGCCGGTGCT
GTGCCAGGACCATGGCCTGACCCCGGACCAGGTCGTGGCCATCGCCAGCCATGGCGGCGGCAAGCAGGCGCTGGAGACGGTGCAGCGGCTGTTGC
CGGTGCTGTGCCAGGACCATGGCCTGACCCCGGACCAGGTGGTGGCCATCGCCAGCAATATTGGCGGCAAGCAGGCGCTGGAGACGGTGCAGCGG
CTGTTGCCGGTGCTGTGCCAGGACCATGGCCTGACCCCGGACCAGGTGGTGGCCATCGCCAGCAATATTGGCGGCAAGCAGGCGCTGGAGACGGT
GCAGCGGCTGTTGCCGGTGCTGTGCCAGGACCATGGCCTGACCCCGGACCAGGTCGTGGCCATCGCCAGCCATGGCGGCGGCAAGCAGGCGCTGG
AGACGGTGCAACGGCTGTTGCCGGTGCTGTGCCAGGACCATGGCCTGACCCCGGACCAAGTCGTGGCCATCGCCAGGCACGATGGCGGCAAGCAG
GCGCTGGAGACGGTGCAGCGGCTGTTGCCGGTGCTGTGCCAGGCCCATGGCCTGACCCCAGACCAAGTGGTGGCCATCGCCAACAATAACGGCGG
CAAGCAGGCGCTGGAGACGGTGCAGCGGCTGTTGCCGGTGCTGTGCCAGGCCCATGGCCTGACCCCGGACCAAGTGGTGGCCATCGCCAGCCACG
ATGGCGGCAAGCAGGCGCTGGAGACGGTGCAGCGGCTGTTGCCGGTGCTGTGCCAGGCCCATGGCCTGACCCCGGACCAGGTGGTGGCCATCGCC
AGCCACGATGGCGGCAAGCAGGCGCTGGAGACGGTGCAGCGGCTGTTGCCGGTGCTGTGCCAGGACCATGGCCTGACCCCGGACCAGGTGGTGGC
CATCGCCAGCCACGATGGCGGCAAGCAGGCGTTGGAGACGGTGCAGCGGCTGTTGCCGGTGCTGTGCCAGGACCATGGCCTGACCCCGGACCAGG
TCGTGGCCATCGCCAGCAATATTGGCGGCAAGCAGGCGCTGGAGACGGTGCAGCGGCTGTTGCCGGTGCTGTGCCAGGACCATGGCCTGACCCCG
GACCAGGTGGTGGCCATCGCCAGCAATATTGGCGGCAAGCAGGCGCTGGAGACGGTACAGCGGCTGTTGCCGGTGCTGTGCCAGGACCATGGCCT

GACCCCGGACCAGGTGGTGGCCATCGCCAACAATAACGGCGGCAAGCAGGCGCTGGAGACGGTGCAGCGGCTGGTGCCGGTGCTGTGCCAGGACC
ATGGCCTGACCCAGGACCAGGTGGTGGCCATCGCCAGCAATATTGGCGGCAAGCAGGCGCTGGAGACGGTGCAGCGGCTGTTGCCGGTGCTGTAC
CAGGACCATGGCCTGACCCAGGACCAGGTGGTGGCCATCGCCAGCCACGATGGCGGCAAGCAGGCGCTGGAGACGGTGCAGCGGCTGTTGCCGGT
GCTGTGCCAGGACCATGGCCTGACCCCGGACCAAGTGGTGGCCATCGCCAGCCACGATGGCGGCAAACAGGCGCTGGAGACGGTGCAGCGGCTGT
TGCCGGTGCTGTGCCAGGAACATGGCCTGACCCCGGACCAAGTGGTGGCCATCGCCAGCCACGATGGCGGCAAGCAGGCGCTGGAGACGGTGCAG
CGGCTGTTGCCGGTGCTGTGCCAGGACCATGGCCTGACCCCGGACCAGGTGGTGGCCATCGCCAGCCATGGCGGCGGCAAGCAGGCGCTGGGGAC
GGTGCAACGGCTGTTGCCGGTGCTGTGCCAGGACCATGGCCTGACCCCGGACCAAGTGGTGGCCATCGCCAACAATAACGGCGGCAAGCAGGCGC
TGGAGACGGTGCAGCGGCTGTTGCCGGTGCTGTGCCAGGACCATGGCCTGACCCCGGACCAAGTGGTGGCCATCGCCAACAATAACGGCGGCAAG
CAGGCGCTGGAGACGGTGCAGCGGCTGTTGCCGGTGCTGTGCCAGGCCCATGGCCTGACCCCGGACCAAGTGGTGGCCATCGCCAGCCACGATGG
CGGCAAGCAGGCGCTGGAGACGGTGCAGCGGCTGTTGCCGGTGCTGTGCCAGGACCATGGCCTGACCCCGGCCCAGGTGGTGGCCATCGCCAGCA
ATAGCGGCGGCAAGCAGGCGCTGGAGACGGTGCAACGGCTGTTGCCGGTGCTGTGCCAGGACCATGGCCTGACCCCGGCCCAGGTCGTGGCCATC
GCCAACAATAACGGCGGCAAGCAGGCGCTGGAGACGGTGCAGCGGCTGTTGCCGGTGCTGTGCCAGGACCATGGCCTGACCCCGGACCAGGTCGT
GGCCATCGCCAGCCACGATGGCGGCAAGCAGGCGCTGGAGACGGTGCAGCGGCTGTTGCCGGTGCTGTGCCAGGACCATGGCCTGACCCTGGACC
AGGTGGTGGCCATCGCCAGCAATGGCGGCAAGCAGGCGCTGGAGACGGTGCAGCGGCTGTTGCCGGTGCTGTGCCAGGACCATGGCCTGACCCCG
AACCAGGTCGTGGCCATCGCCAGCAATAGTGGCGGCAAGCAGGCGCTGGAGACGGTGCAGCGGCTGTTGCCGGTGCTGTGCCAGGACCATGGCCT
GACCCCGAACCAGGTGGTGGCCATCGCCAGCAATGGCGGCAAGCAGGCGCTGGAGAGCATTGTTGCCCAGTTATCTCGCCCTGATCCGGCGTTGG
CCGCGTTGACCAACGACCACCTCGTCGCCTTGGCCTGCCTCGGCGGACGTCCTGCCCTGGATGCAGTGAAAAAGGGATTGCCGCACGCGCCGGAA
TTGATCAGAAGAATCAATCGCCGTATTCCCGAACGCACGTCCCATCGCGTTCCCGACCTCGCGCACGTGGTTCGCGTGCTTGGTTTTTTCCAGAG
CCACTCCCACCCAGCGCAAGCATTCGATGACGCCATGACGCAGTTCGGGATGAGCAGGCACGGCTTGGTACAGCTCTTTCGCAGAGTGGGCGTCA
CCGAATTCGAAGCCCGCTACGGAACGCTCCCCCCAGCCTCGCAGCGTTGGGACCGTATCCTCCAGGCATCAGGGATGAAAAGGCCAAACCGTCC
CCTACTTCAGCTCAAACACCGGATCAGGCGTCTTTGCATGCATTCGCCGATTCGCTGGAGCGTGACCTTGATGCGCCCAGCCCAATGCACGAGGG
AGATCAGACGCGGGCAAGCAGCCGTAAACGGTCCCGATCGGATCGTGCTGTCACCGACCCCTCCACACAGCAATCTTTCGAGGTGCGCGTTCCCG
AACAGCGCGATGCGCTGCATTTGCCCCTCAGCTGGAGGGTAAAACGCCCGCGTACCAGGATCGGGGGCGGCCTCCCAGATCCTGGTACGCCCATC
GCTGCCGACCTGGCAGCGTCCAGCACCGTGATGTGGGAACAAGATGCGGCCCCCTTCGCAGGGGCAGCGGATGATTTCCCGGCATTCAACGAAGA
GGAGCTCGCATGGTTGATGGAGCTATTGCCTCAGTCAGGCTCAGTCGGAGGGACGATC
